# Divergent Synthesis of 5,7-Diazaullazines Derivatives through a Combination of Cycloisomerization with Povarov or Alkyne–Carbonyl Metathesis

**DOI:** 10.3390/molecules29092159

**Published:** 2024-05-06

**Authors:** Jonas Polkaehn, Peter Ehlers, Alexander Villinger, Peter Langer

**Affiliations:** 1 Institute of Chemistry, University Rostock, Albert-Einstein-Str. 3a, 18059 Rostock, Germany; 2 Leibniz Institute for Catalysis (LIKAT), University Rostock, Albert-Einstein-Str. 29a, 18059 Rostock, Germany

**Keywords:** alkyne-carbonyl-metathesis, cycloisomerisation, polycyclic heteroaromatic hydrocarbons, Povarov reaction, ullazines

## Abstract

Ullazines and their π-expanded derivatives have gained much attention as active components in various applications, such as in organic photovoltaic cells or as photosensitizers for CO_2_ photoreduction. Here, we report the divergent synthesis of functionalized diazaullazines by means of two different domino-reactions consisting of either a Povarov/cycloisomerization or alkyne–carbonyl metathesis/cycloisomerization protocol. The corresponding quinolino-diazaullazine and benzoyl-diazaullazine derivatives were obtained in moderate to good yields. Their optical and electronic properties were studied and compared to related, literature-known compounds to obtain insights into the impact of nitrogen doping and π-expansion.

## 1. Introduction

**Figure 1:**
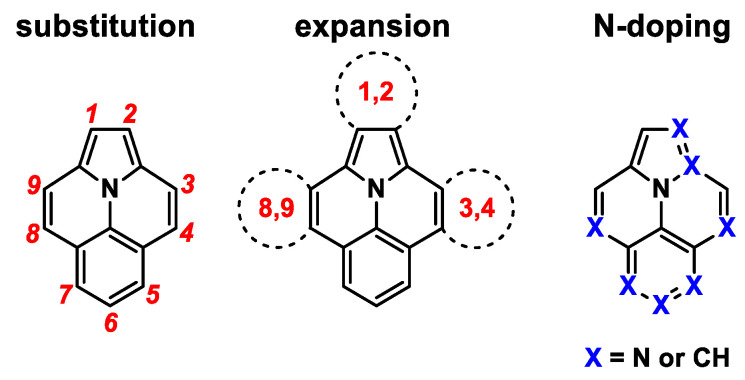
Potential modifications to ullazine.

Ullazine contains 16-π-electrons, is isoelectronic to pyrene, and is a representative of polycyclic heteroaromatic hydrocarbons (PAHs) [1]. It has been known since its first synthesis in 1983 [2], but has gained much interest for applications in organic electronics during the last decade [1,3,4,5,6,7] since Grätzel et al. disclosed its promising application in dye-sensitized solar cells in 2013 [1]. The aromatic 14-π-electron resonance structure is a key factor, consisting of an electron-accepting iminium center surrounded by an electron-donating annulene. Hence, in the following years, several approaches, such as substitution, π-extension or heteroatom doping, were employed to modify the properties of various applications. Substitutions on the ullazine core are known in each position (1–9) of the ullazine core (Figure 1) [1,2,8,9,10,11,12]. Positions 4, 5, and 7 can be addressed by electrophilic substitution, whereas position 5 is the most active of these three [1,13,14]. Functionalization on the other positions can be achieved through the employment of suitable precursors during ullazine formation, for example, by the double benzannulation of *N*-phenylpyrroles [1,2,8,9]. The expansion of the π-systems is mainly executed by 1,3-dipolar cycloaddition with azomethine ylides, which leads to symmetrical dibenzoullazines with functionalization of positions 1 and 2 [15,16,17,18,19,20]. Fused dithieno- and dipyridoullazines are obtained by the photochemical cyclization of 3,9-diarylullazines [21]. In addition to the extension of the π-system and variations in the substitution pattern, doping of the scaffold by heteroatoms has been used to alter the inherent properties of ullazines. Several examples of N-, O-, and B-doped ullazines are known in the literature [9,22,23,24,25,26,27,28,29].

**Scheme 1:**
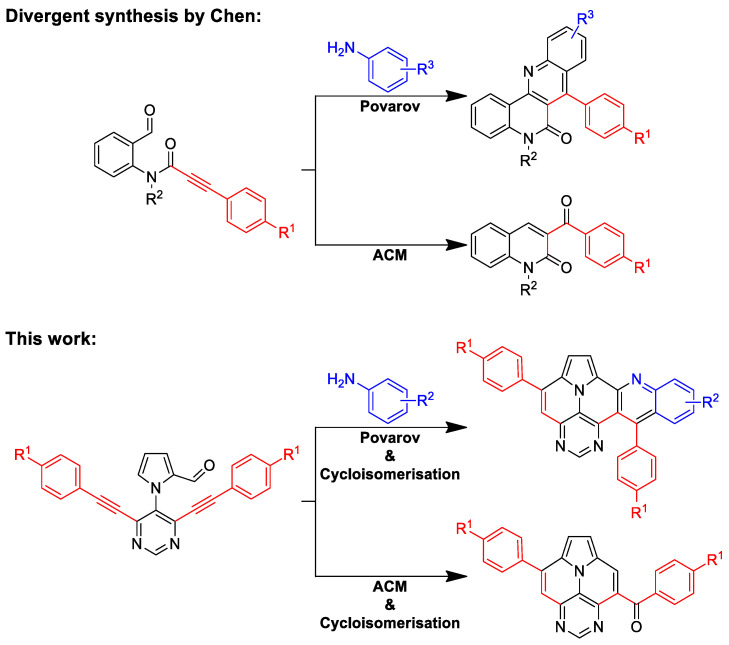
Divergent synthesis of Chen and our approach with the synthesis quinolino-diazaullazines and benzoyl-diazaullazines.

Recently, we studied the synthesis of 5,7-diazaullazines [29]. The incorporation of a pyrimidine ring into the ullazine scaffold leads to strongly altered optical properties through the stabilization of the HOMO and LUMO energies with strong intramolecular charge transfer (ICT) properties and improved quantum yields. Moreover, we reported the synthesis of quinolino-azaullazines, which show bathochromically shifted absorption and emission features compared to their azaullazine subunit or related dibenzoullazines [30]. Hence, we were interested in combining these two approaches, π-expansion and increased N-doping of the ullazine structure, to study the impact of these structural modifications on the photophysical properties. Retrosynthetic analysis revealed that respective quinolino-diazaullazines are accessible by a combination of the Povarov reaction and cycloisomerization, similarly to the synthesis of quinolino-azapyrenes and quinolino-azaullazines [30,31]. Interestingly, the same starting material might also undergo alkyne–carbonyl metathesis (ACM) followed by cycloisomerization for the construction of novel benzoyl-diazaullazine derivatives. Hence, we report a divergent synthesis of quinolino-diazaullazines and benzoyl-diazaullazine from the same precursor through careful choice of the employed reaction conditions. During our studies, the group of Chen reported a related approach for the selective synthesis of pharmaceutically relevant naphthyridinones and quinolinones via either Povarov or ACM reaction, respectively, starting from formyl-phenylpropialamide (Scheme 1) [32].

### 1.1. Synthesis

**Scheme 2:**
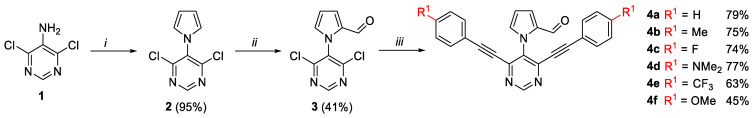
Synthesis of ACM and Povarov precursors **4a**–**f**; *i*: 2,5-dimethoxytetrahydrofuran, acetic acid, 1,2-dichloroethane, reflux, 3 h. *ii*: POCl_3_ (2.0 eq.), DMF, 100 °C, 3 h. *iii*: alkyne (3 eq.), PdCl_2_(CH_3_CN)_2_ (0.06 eq.), XPhos (0.12 eq.), CuI (0.04 eq.), HN^i^Pr_2_, 1,4-dioxane, 90 °C, 24 h.

Our synthetic methodology started with the synthesis of an eligible pyrrolopyrimidine **4**, which can undergo ACM and Povorav reactions followed by cycloisomerization. Starting from commercially available 4,6-dichloropyrimidin-5-amine (**1**), the Clausson–Kaas reaction gave pyrrolopyrimidine **2** in excellent yield. Subsequent Vilsmeier–Haak reaction proceeded selectively on the pyrrole ring, resulting in a mixture of the 2- and 3-formylpyrrole derivatives from which the respective precursor **3**, with aldehyde function in position 2, was separated in 41% yield. Finally, the desired precursors **4a–f** were obtained by two-fold Sonogashira reaction in good yield (Scheme 2) [29,30]. 

Starting material **4a** was chosen as the model substrate to study the divergent synthesis of quinolino-diazapyrene **5a** and benzoyl-diazaullazine **6a**. At first, we focused on the optimization of the Povarov reaction with subsequent cycloisomerization in a one-pot protocol, as both reactions are typically mediated by strong acids. In particular, product **5a** derives from three individual reaction steps: Schiff-base formation, Povarov reaction and cycloisomerization.

The synthesis of **5a** is initially based on the addition of aniline and FeCl_3_ [33]. FeCl_3_ has proven to be a powerful catalyst for the synthesis of PAHs via Povarov reaction [30,31,32,33]. After the Povarov reaction has ceased, *p*-TsOH, as a Brønsted acid, is added to the reaction mixture, which initiates the final ring closure through the activation of the second triple bond. As a starting point, we tested the reaction conditions that were recently employed for the synthesis of related quinolino-azaullazine derivatives, but only traces of the desired product **5a** were obtained (Table 1) [30]. Since starting material **4a** was still detected by TLC control, we decided to focus on the first reaction step—the Povarov reaction. The elevation of the reaction temperature and the amount of FeCl_3_ led to an improvement of 38% in the yield of final product **5a** using 1 eq. of FeCl_3_ at 140 °C. Interestingly, the formation of **5a** was detected by the TLC control before the addition of Brønsted acid. However, **5a** was isolated in a reduced 21% yield even when access of FeCl_3_ was employed. Hence, a strong Brønsted acid is required to drive the reaction to completion. Next, we turned our attention to the final cyclization step. The application of *p*-TsOH·H_2_O proved to be superior to the employment of methanesulfonic acid (MsOH). Different amounts of *p*-TsOH∙H_2_O and an increased reaction time improved the overall product yield to 45%, which corresponds to a theoretical yield of more than 75% for each reaction step in this one-pot process. 

In the following, we analyzed the synthesis of **6a**. It is known from the literature that both cycloisomerization and the ACM reaction are promoted efficiently by Brønsted acids (Table 2) [24,34]. Hence, we first tested similar conditions as for the Povarov but without the addition of aniline [32]. To our delight, product **6a** was isolated in 59% after 16 h. Reducing the amount of *p*-TsOH to 20 eq. and lowering the reaction temperature to 120 °C gave an improved yield of 68%, while the use of less acid or the employment of MsOH led to inferior results. Finally, we reduced the reaction time to 6 h without compromising the isolated yield.

**Scheme 3:**
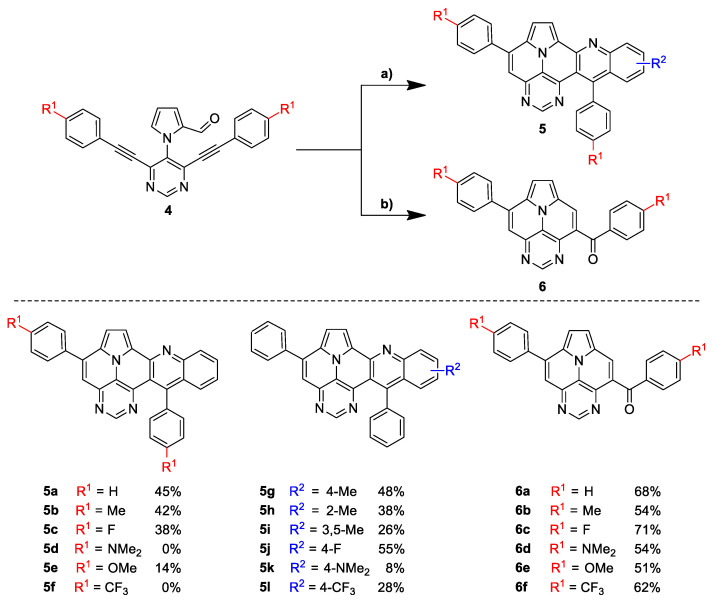
Synthesis of final products **5a**–**l** by Povarov/cycloisomerization and **6a**–**f** by ACM/cycloisomerization. (**a**) 1. FeCl_3_ (1 eq.), corresponding aniline (1.2 eq.), xylene, 140 °C, 3 h; 2. *p*-TsOH∙H_2_O (30 eq.), xylene, 140 °C, 6 h. (**b**) *p*-TsOH∙H_2_O (20 eq.), xylene, 120 °C, 6 h.

With optimized reaction conditions for both reactions, we studied the scope and limitations of our developed methodologies through an examination of different precursors, **4a**–**f** and the employed anilines (Scheme 3). Similar yields of the respective quinoline-diazaullazine (**5a**–**c**) were obtained for weak sigma donors or acceptors on the aryl alkyne moiety. However, stronger donors and acceptors led to inferior results (**5d**–**f**). One explanation for this could be the competitive reaction (ACM), as only the product **6d** was obtained during the synthesis of **5d**. In particular, **4d** did not react to product **5d,** and instead ACM product **6d** was detected as the only product. However, whether strong donors/acceptors lead to an enhancement of the ACM or to an inhibition of imine formation/[4 + 2]-cycloaddition cannot be clarified conclusively. Similar effects were observed by changing the substitution pattern of the employed aniline. While 4-methyl or 4-F substituents gave good yields (**5g**, **5j**), the yield dropped when CF_3_ or NMe_2_ groups were present. Moreover, sterical effects lead to reduced yield (**5h**, **5i**).

The ACM reaction seems to be less sensitive to functional groups and compounds **6a**–**f** were isolated in moderate to good yields independently from the substitution pattern. However, donor-substituted products (**6b**, **6d**, **6e**) were obtained in slightly lower yields. 

**Figure 2:**
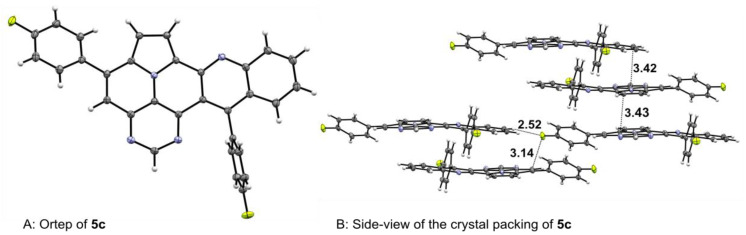
X-ray structures of **5c**.

Crystals of **5c** were grown via the slow evaporation of its chloroform solution, making them suitable for X-ray crystal structure analysis (Figure 2) [35]. The obtained crystal structure contained two co-crystallized CHCl_3_ molecules per unit cell, which were omitted for better illustration. Both *p*-tolyl residues were twisted out of plane from the core structure by dihedral angles of 40° on the diazaullazine moiety and 84° on the quinoline part. Moreover, the crystal lattice showed a slipped antiparallel π-π-stacking with a spacing of 3.43 Å and 3.41 Å between the quinoline and diazaullazine entities, respectively. Different stacks within the crystal lattice were stabilized by close F-π (3.14 Å) and F-HC (2.52 Å) contacts.

**Figure 3:**
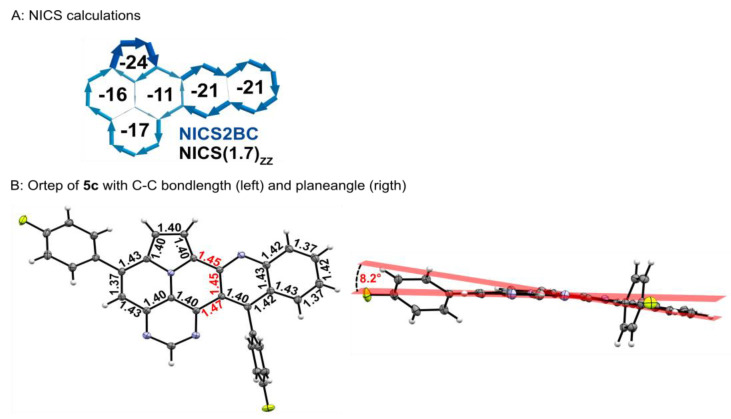
(**A**) NICS calculations for pyrimido[4′,5′,6′:9,1]pyrrolo[2′,1′,5′:4,5,6]quinolizino[3,2-*b*]quinoline: NICS2BC graphs (current was calculated from NICS(1.25)_ZZ_ strength relative to I_ref_ (ring current of benzene, 11.5 nA T^−1^)). In the center of each ring are the respective NICS(1.7)_ZZ_ values. (**B**) Ortep of **5c** with C-C bond length of the core structure (left) and plane angle between the pyrimido-indolizine and the quinoline moieties.

NICS(1.7)_ZZ_, as a criterion of the local aromaticity, and bond currents, using the BC-Wizard by Gershoni-Poranne et al., were calculated to obtain detailed insights into the aromatic behavior of **5** (Figure 3) [36,37]. A global diatropic ring current is apparent, accompanied by two local diatropic ring currents within the pyrrole and the pyrimidine rings. Furthermore, two diatropic semi-global currents are identifiable for the quinoline and the pyrimido-indolizine units. The central benzene ring connecting both moieties possesses the lowest NICS(1.7)_ZZ_ values of the entire molecule. This observation coincides with the experimentally measured bond lengths. The C-C bond lengths between the quinoline and pyrimido-quinoline moieties are the longest within the molecular scaffold (1.45–1.47 Å; marked in red), indicating a reduced delocalization of π-electrons and leading to a slight curvature between the pyrimido-indolizine and the quinoline moieties by 8.2°. Similar results were observed for the related quinolino-azapirone and quinolino-azaullazine [30,31].

### 1.2. Photophysical Properties

**Figure 4:**
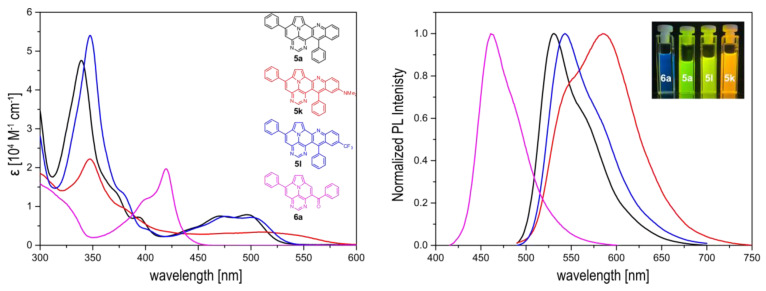
UV/Vis (**left**) and PL spectra ((**right**), λ_ex_ = 480 nm (**5a**,**k**,**l**), λ_ex_ = 400 nm (**6a**)) of the compounds shown in CH_2_Cl_2_ (c = 10^−5^ M) at 20 °C.

The optical properties were studied via steady-state absorption- and emission spectroscopy (Figure 4). We focused on products **5a**, **5k**, and **5l**, which differ from the substitution pattern directly on the polycyclic scaffold. The impact of the attached phenyl rings on the optical properties is known to be limited due to its twisted orientation, and will not be further analyzed [30,31]. The results will be compared with ACM product **6a**. The spectroscopic data are shown in Table S2.

Only slight differences between **5a** and **5l** containing an electron-withdrawing CF_3_-group are evident. Thus, both exhibit similar extinction coefficients and a fine structure of the absorption spectra, with slightly red-shifted absorption and emission maxima of **5l**. The fluorescence quantum yields of both compounds are also very similar and relatively high, at 53% and 52%, respectively [38]. Compound **5k** displays a broadened, unstructured absorption with smaller extinction coefficients over the entire spectrum, with significantly red-shifted absorption and emission maxima and noticeably reduced quantum yields (29%). These observations could indicate an ICT for compound **5k**.

To investigate its potential ICT character, we performed solvatochromic studies with **5a** and **5k** and compared the calculated dipole moments of their S_0_ and the S_1_ transition states. Only minor changes in the calculated dipole moments, as well as in the absorption and emission features, in solvents of different polarities (toluene, CH_2_Cl_2_, acetonitrile, ethanol) were determined for both compounds (Figure S1, Supplementary Materials). Hence, the occurrence of ICT properties due to the presence of the NMe_2_ group can be neglected.

In contrast, **6a** features a completely different structure of the absorption spectrum and strongly blue-shifted absorption and emission spectra with higher extinction coefficients of the lowest energy band (Figure 4). The fine structure and the location of the lowest energy band is very similar to that of the symmetrically 3,9-substituted 5,7-diazaullazines (**8**) [29]. However, **6a** has a much lower fluorescence quantum yield compared to **8**. A similar impact of benzoyl groups was previously observed for benzoyl functionalized azapyrene derivatives [39,40]. Interestingly, the quantum yield is comparable to 2-azaullazines, containing a benzoyl group and an additional CF_3_ group, whereby the emission maxima of **5a** are significantly blue-shifted by ~140 nm [41].

**Figure 5:**
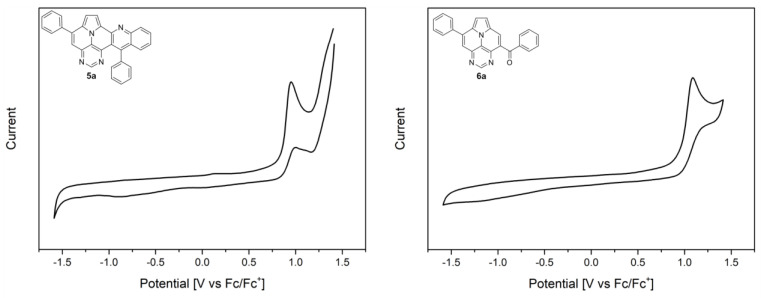
Cyclic voltammograms of **5a** and **6a**. Measured in CH_2_Cl_2_ (0.001 M) with 0.1 M n-Bu_4_NPF_6_ as a supporting electrolyte, glassy carbon working electrode, and Pt counter-electrode, with ferrocene as a standard, at a scan rate of 100 mV/s.

To gain insights into the redox properties of the different core structures, **5a** and **6a**, we performed cyclic voltammetry (CV) measurements in dichloromethane (Figure 5). Both compounds exhibit an irreversible oxidation potential, while **5a** is slightly more easily oxidized (0.95 V; onset potential of 0.85 V) than compound **6a** (1.08 V; onset potential of 0.96 V), which corresponds to the experimentally deduced HOMO energies of −5.65 eV (**5a**) and −5.76 eV (**6a**) [42]. As expected, the presence of a withdrawing benzoyl group on the ullazine scaffold leads to a lower oxidation potential compared to diazaullazine **8** [29]. Similarly, the exchange of a pyridine ring of **7** with a more electron-poor pyrimidine ring (**5a**) leads to a reduced oxidation potential (Table 3) [30]. No reduction event is observed within the analyzed potential window of dichloromethane.

**Figure 6:**
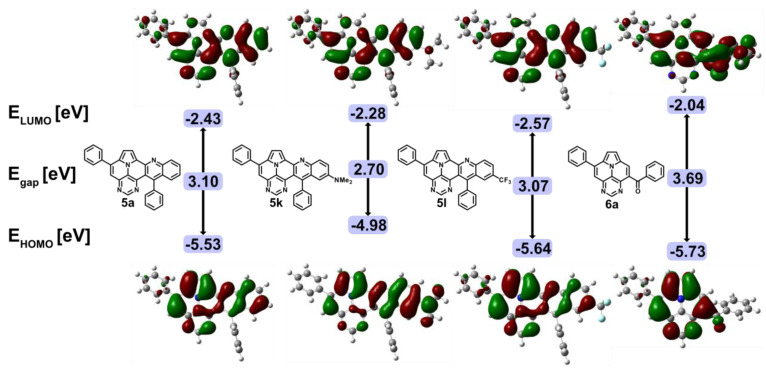
Frontier orbitals of **5a**, **5k**, **5l,** and **6a**, and energy levels calculated at the B3LYP/6-31G(d,p) level of theory within IEFPCM in CH_2_Cl_2_.

Density functional theory (DFT) calculations were performed for **5a**, **5k**, **5l,** and **6a** to obtain an improved understanding of the electronic properties and to disclose the impact of N-doping (Figure 6) [43]. The frontier orbitals of all three quinolino-diazaullazines are very similar and are mainly located on the core structures, with no contribution of the aryl substituents. Localization of the frontier orbitals, as well as the HOMO-LUMO gap, is comparable, as could be assumed from their previously discussed optical properties. The depiction of the HOMO and LUMO reveals no additional contribution by the CF_3_ group to either of the frontier orbitals. In contrast, a pertinent contribution by the NMe_2_ group of **5k** to the HOMO and, to a lesser extent, to the LUMO is apparent, leading to destabilized HOMO and LUMO energies, with a greater impact on the former. A comparison of **5a** with **7** shows the stabilization of both the HOMO and LUMO by ~0.30 eV due to the incorporation of a pyrimidine instead of a pyridine ring within the ullazine scaffold (Table 3) [30].

**6a** has an increased HOMO-LUMO gap (3.69 eV), which is due to a destabilized LUMO and a stabilized HOMO compared to product **5**. Interestingly, the HOMO–LUMO gap is exactly the same as for 3,9-substituted 5,7-diazaullazine (**8**), with both the HOMO and LUMO energies stabilized by 0.13 eV [29]. However, both compounds show different contributions to their respective frontier orbitals. While the HOMO and LUMO of **8** are mainly localized on the ullazine core structure, the strong participation of the benzoyl substituent of **6a** is observed on the LUMO.

A comparison of symmetrical (**8**) and unsymmetrical (**6a**) substituted diazaullazines, as well as π-expanded aza-(**7**) and diazaullazines (**5a**), reveal the impact of nitrogen doping, π-expansion, and the substitution pattern on the properties of these ullazine derivatives (Table 3) [29,30].

The absorption and emission properties of **5a** are comparable to those of quinolino-azaullazine (**7**) However, the installment of a pyrimidine moiety instead of a pyridine unit leads to red-shifted absorption and emission spectra. **5a** exhibits the highest quantum yields of the compared substances, which are twice as high as for related compound **7**. The HOMO and LUMO energies are stabilized by ~0.30 eV. Comparing compounds **5a** and **8** reveals significantly red-shifted absorption and emission spectra. The annulation of a quinoline moiety on the diazaullazine moiety leads to a bathochromical shift of 75 nm for the absorption and 91 nm for the emission spectrum.

The differences between **6a** and **8** are rather small. Both have similar absorption and emission spectra, as well as similar HOMO and LUMO energies, which differ by 0.13 eV. This can be explained by the fact that these properties are mainly specified by the core structure and the substituents only have a minor influence on this. However, the introduction of a benzoyl function results in a noticeable quenching of the fluorescence and thus **8** has a 3.5 times higher quantum yield than **6a**.

## 2. Conclusions

We developed a divergent synthesis of π-expanded diazaullazine (quinolino-diazaullazines) and 9-benzoyl-diazaullazines through a one-pot multi-step procedure consisting of a Povarov/cycloisomerization or ACM/cycloisomerization protocol, respectively. Moderate to good yields of the desired products were obtained and selected compounds were studied by UV/Vis, fluorescence, and cyclovoltammetric measurements, which have been underpinned by DFT calculations. A comparison with related compounds offered insights into the impact of the substitution pattern and degree of N-doping on the optical and electrochemical properties. In particular, π-expansion by the fusion of a quinoline moiety leads to bathochromically shifted absorption and emission spectra accompanied by improved quantum yields, while benzoyl substituents lead to blue-shifted absorption and emission bands and reduced quantum yields.

## 3. Materials and Methods

### 3.1. General Information

The nuclear magnetic resonance spectra (^1^H/^13^C/^19^F NMR) were obtained using a Bruker AVANCE 300 III, 250 II, or 500. Chemical shifts (δ) were calibrated with respect to residual solvent signals of deuterated solvents CDCl_3_ (δ = 7.26 ppm/77.0 ppm). Spin–spin correlation-induced multiplicities were denoted as follows: s = singlet; d = doublet; dd = double doublet; ddd = doublets of doublets; pt = pseudo triplet; m = multiplet, accompanied by their coupling constants (J). Infrared spectra (IR) were measured using attenuated total reflection (ATR) with a Nicolet 380 FT-IR spectrometer. Signal characteristics were described in terms of wavenumbers (ῦ) and absorption strengths, categorized as very strong (vs), strong (s), medium (m), or weak (w). UV/Vis spectra were acquired using a Cary 60 UV−vis spectrophotometer, and emission spectra were obtained with an Agilent Cary Eclipse fluorescence spectrophotometer. Cyclic voltammograms (CVs) were conducted at room temperature in CH_2_Cl_2_ (c = 10^−3^ M) with 0.1 M *n*-Bu_4_NPF_6_ as the supporting electrolyte, a glassy carbon working electrode, ANE2 (Ag/AgNO_3_ 0.01 M in CH_3_CN), as a reference electrode, and Pt as a counter-electrode (0.5 mm diameter platinum wire). Ferrocene (c = 10^−3^ M, in CH_3_CN) served as an external standard at a scan rate of 100 mV/s. The voltammograms were recorded on a PalmSense EmStat 3 blue potentiostat. The working electrode is a 3 mm diameter, glassy, carbon disk electrode coated with KeI-F, polished using aqueous alumina slurry (0.03 μm alumina powder) on a polishing pad. Solvents were deoxygenated by argon purging. Potentials were referenced to as Fc^+^/Fc, with a reductive scan direction starting at 1.5 V and a switching potential of −1.5 V, plotted using the IUPAC conventions. Mass spectra (MS/HRMS) were acquired using instruments coupled with preceding gas chromatography (GC) or liquid chromatography (LC). The samples were ionized either by electron impact ionization (EI) using an Agilent 6890/5973 or Agilent 7890/5977 GC-MS with a HP-5 capillary column and helium carrier gas, or by electron spray ionization (ESI) using an Agilent 1200/6210 Time-of-Flight (TOF) LC−MS. Melting points (mp) were determined using a Micro-Hot-Stage GalenTM III Cambridge Instruments without correction. X-ray single-crystal structure analysis was performed using a Bruker Apex Kappa-II CCD diffractometer.

### 3.2. Analytical Data

#### 3.2.1. General Procedure A for the Synthesis of 5,13-Diphenylpyrimido[4′,5′,6′:9,1]pyrrolo[2′,1′,5′:4,5,6]quinolizino[3,2-*b*]quinoline (**5a**–**l**)

A total of 100 mg of **4a**–**f**, 1.2 eq. of the corresponding aniline and 1 eq. FeCl_3_ were suspended in 4 mL xylene and stirred at 140 °C for 3 h. Subsequently, 20 eq. *p*-TsOH∙H_2_O were added to the reaction mixture and the solution was stirred at 140 °C for another 6 h. The reaction was quenched with NaHCO_3_-solution, extracted three times with 50 mL CH_2_Cl_2_ and dried over Na_2_SO_4_. The solvent was distilled in vacuo and the residue was purified by column chromatography (CH_2_Cl_2_/EtOAc) to yield the desired products (**5a**–**l**).

*5,13-diphenylpyrimido[4′,5′,6′:9,1]pyrrolo[2′,1′,5′:4,5,6]quinolizino[3,2-b]quinoline* (**5a**)

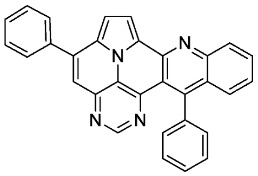

According to general procedure A, the title compound **5a** was obtained as an orange solid in 45% yield (54 mg, 0.121 mmol). *R*_f_ 0.50 (CH_2_Cl_2_/EtOAc 10:1). Mp. 284–286 °C. ^1^H NMR (500 MHz, CDCl_3_) δ = 8.83 (s, 1H), 8.31–8.27 (m, 1H), 8.24 (d, *J* = 4.3 Hz, 1H), 7.89–7.86 (m, 2H), 7.84 (ddd, *J* = 8.3 Hz, *J* = 6.6 Hz, *J* = 1.4 Hz, 1H), 7.66 (dd, *J* = 8.6 Hz, *J* = 1.4 Hz, 1H), 7.63–7.54 (m, 7H), 7.46 (ddd, *J* = 8.3 Hz, *J* = 6.7 Hz, *J* = 1.2 Hz, 1H), 7.42 (d, *J* = 4.3 Hz, 1H), 7.40–7.37 (m, 2H). ^13^C NMR (126 MHz, CDCl_3_) δ = 153.4, 150.2, 149.6, 146.9, 146.8, 144.5, 140.9, 138.7, 137.3, 131.5, 129.4, 129.1, 128.9, 128.7, 128.2, 127.9, 127.6, 127.5, 127.2, 126.3, 126.1, 122.1, 117.5, 116.8, 112.2, 109.1. IR (ATR, cm^−1^): ṽ = 1605 (s), 1578 (m), 1500 (m), 1449 (m), 1405 (m), 863 (m), 777 (s), 734 (s), 705 (vs), 612 (s), 554 (s). MS (EI, 70 eV): *m*/*z* (%) = 446 (86, M^+^), 445 (92), 444 (28), 382 (56), 381 (100), 380 (89), 379 (28), 354 (26), 223 (49), 222 (52), 208 (31). HRMS (ESI-TOF): calculated for C_31_H_19_N_4_ ([M + H]^+^) 447.1609, found 447.1602.
*5,13-di-p-tolylpyrimido[4′,5′,6′:9,1]pyrrolo[2′,1′,5′:4,5,6]quinolizino[3,2-b]quinoline* (**5b**)

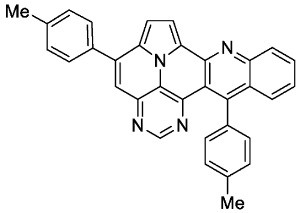

According to general procedure A, the title compound **5b** was obtained as an orange solid in 42% yield (50 mg, 0.105 mmol). *R*_f_ 0.78 (CH_2_Cl_2_/EtOAc 15:1). Mp. 303–306 °C. ^1^H NMR (250 MHz, CDCl_3_) δ = 8.83 (s, 1H), 8.22–8.16 (m, 1H), 8.13 (d, *J* = 4.3 Hz, 1H), 7.79–7.69 (m, 3H), 7.64 (ddd, *J* = 8.7 Hz, *J* = 1.5 Hz, *J* = 0.7 Hz, 1H), 7.49 (s, 1H), 7.44–7.35 (m, 5H), 7.33 (d, *J* = 4.3 Hz, 1H), 7.32–7.21 (m, 2H), 2.60 (s, 3H), 2.48 (s, 3H).^13^C NMR (63 MHz, CDCl_3_) δ = 153.3, 150.4, 149.4, 146.8, 146.7, 144.4, 140.7, 139.5, 137.0, 135.6, 134.4, 131.3, 129.7, 128.8, 128.8, 128.6, 128.5, 127.9, 127.4, 127.3, 126.1, 125.8, 121.9, 117.0, 116.7, 112.0, 108.9, 21.5, 21.4. IR (ATR, cm^−1^): ṽ = 1603 (s),1498 (m), 820 (s), 771 (m), 748 (vs), 734 (s), 725 (s), 612 (s), 556 (s). MS (EI, 70 eV): *m*/*z* (%) = 474 (92, M^+^), 473 (100), 472 (7), 238 (6), 237 (28), 236 (10), 230 (21), 229 (13), 228 (7), 222 (7), 215 (7). HRMS (ESI-TOF): calculated for C_33_H_23_N_4_ ([M + H]^+^) 475.1923, found 475.1926.
*5,13-bis(4-fluorophenyl)pyrimido[4′,5′,6′:9,1]pyrrolo[2′,1′,5′:4,5,6]quinolizino[3,2-b]quinoline* (**5c**)

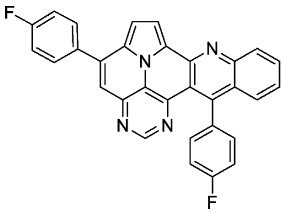

According to general procedure A, the title compound **5c** was obtained as an orange solid in 38% yield (44 mg, 0.091 mmol). *R*_f_ 0.78 (CH_2_Cl_2_/EtOAc 15:1). Mp. 318–320 °C. ^1^H NMR (500 MHz, CDCl_3_) δ = 8.86 (s, 1H), 8.32 (d, *J* = 8.5 Hz, 1H), 8.27 (d, *J* = 4.3 Hz, 1H), 7.89–7.84 (m, 3H), 7.68–7.65 (m, 1H), 7.54 (s, 1H), 7.50 (ddd, *J* = 8.3 Hz, *J* = 6.6 Hz, *J* = 1.2 Hz, 1H), 7.38 (d, *J* = 4.3 Hz, 1H), 7.36–7.28 (m, 6H). ^13^C NMR (126 MHz, CDCl_3_) δ = 163.5 (d, *J* = 250.0 Hz), 162.6 (d, *J* = 246.3 Hz), 153.4, 149.5, 149.3, 146.8, 146.7, 144.4, 139.9, 134.3 (d, *J* = 3.6 Hz), 133.3 (d, *J* = 3.2 Hz), 131.7, 130.4 (d, *J* = 8.7 Hz), 130.4 (d, *J* = 8.6 Hz),129.0, 127.6, 127.5, 127.2, 126.3, 126.3, 122.1, 117.5, 116.9, 116.2 (d, *J* = 21.8 Hz), 115.3 (d, *J* = 21.6 Hz), 112.4, 109.0. ^19^F NMR (471 MHz, CDCl_3_) δ = −111.4, −114.7. IR (ATR, cm^−1^): ṽ = 1603 (vs),1502 (vs), 1228 (vs), 1158 (s), 835 (vs), 802 (s), 769 (s), 748 (s), 736 (s), 610 (s), 563 (vs). MS (EI, 70 eV): *m*/*z* (%) = 482 (67, M^+^), 481 (100), 480 (42), 453 (13), 241 (27), 240 (71), 231 (16), 226 (37), 225 (32), 217 (16), 216 (17), 213 (14). HRMS (ESI-TOF): calculated for C_31_H_17_F_2_N_4_ ([M + H]^+^) 483.1421, found 483.1425.
*5,13-bis(4-methoxyphenyl)pyrimido[4′,5′,6′:9,1]pyrrolo[2′,1′,5′:4,5,6]quinolizino[3,2-b]quinoline* (**5e**)

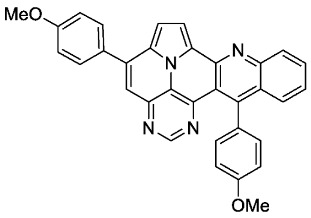

According to general procedure A, the title compound **5e** was obtained as an orange solid in 14% yield (16 mg, 0.032 mmol). *R*_f_ 0.85 (CH_2_Cl_2_/EtOAc 15:1). Mp. 298–301 °C. ^1^H NMR (250 MHz, CDCl_3_/TFA) δ = 9.05 (d, *J* = 4.9 Hz, 1H), 8.92 (s, 1H), 8.61 (d, *J* = 8.6 Hz, 1H), 8.44 (ddd, *J* = 8.6 Hz, *J* = 6.9 Hz, *J* = 1.4 Hz, 1H), 8.31–8.18 (m, 3H), 8.03–7.90 (m, 3H), 7.39–7.21 (m, 6H), 4.06 (s, 3H), 4.01 (s, 3H). ^13^C NMR (63 MHz, CDCl_3_/TFA) δ = 166.0, 163.3, 161.4, 148.6, 146.7, 145.3, 140.6, 140.3, 138.4, 136.1, 131.4, 131.3, 130.8, 130.1, 129.6, 128.1, 127.0, 126.6, 119.9, 119.7, 119.4, 118.4, 116.0, 115.9, 115.1, 111.1, 55.8, 55.7. IR (ATR, cm^−1^): ṽ = 1601 (s),1498 (s), 1243 (vs), 1175 (vs), 1158 (s), 1024 (vs), 835 (s), 785 (s), 762 (vs), 736 (s), 573 (s), 558 (vs). MS (EI, 70 eV): *m*/*z* (%) = 506 (100, M^+^), 505 (99), 463 (5) 462 (13), 420 (4), 419 (8), 254 (7), 253 (19), 232 (8), 231 (5), 210 (9), 197 (5), 196 (5). HRMS (ESI-TOF): calculated for C_33_H_23_N_4_O_2_ ([M + H]^+^) 507.1821, found 507.1828.
*11-methyl-5,13-diphenylpyrimido[4′,5′,6′:9,1]pyrrolo[2′,1′,5′:4,5,6]quinolizino[3,2-b]quinoline* (**5g**)

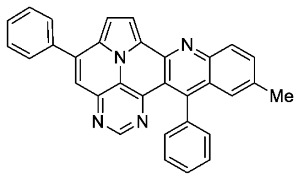

According to general procedure A, the title compound **5g** was obtained as an orange solid in 48% yield (59 mg, 0.128 mmol). *R*_f_ 0.75 (CH_2_Cl_2_/EtOAc 15:1).Mp. 319–322 °C. ^1^H NMR (500 MHz, CDCl_3_) δ = 8.80 (s, 1H), 8.17 (d, *J* = 4.3 Hz, 1H), 8.14 (d, *J* = 8.6 Hz, 1H), 7.87–7.84 (m, 2H), 7.64–7.54 (m, 7H), 7.53 (s, 1H), 7.40–7.36 (m, 4H), 2.44 (s, 3H). ^13^C NMR (126 MHz, CDCl_3_) δ = 153.3, 149.2, 148.2, 146.7, 143.7, 140.7, 138.8, 137.3, 136.1, 134.0, 129.4, 129.0, 128.7, 128.6, 128.6, 128.1, 127.4, 127.3, 127.1, 126.2, 126.2, 122.0, 117.2, 116.7, 111.8, 109.0, 22.0. IR (ATR, cm^−1^): ṽ = 1607 (s),1554 (m), 1492 (m), 1449 (m), 1403 (m), 1325 (m), 814 (m), 777 (vs), 754 (m), 703 (vs), 596 (m), 556 (s). MS (EI, 70 eV): *m*/*z* (%) = 460 (91, M^+^), 459 (100), 458 (11), 457 (7), 230 (28), 229 (29), 228 (15), 222 (18), 215 (8), 214 (13), 208 (7). HRMS (ESI-TOF): calculated for C_32_H_21_N_4_ ([M + H]^+^) 461.1766, found 461.1771.
*9-methyl-5,13-diphenylpyrimido[4′,5′,6′:9,1]pyrrolo[2′,1′,5′:4,5,6]quinolizino[3,2-b]quinoline* (**5h**)

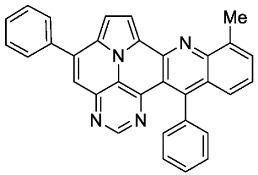

According to general procedure A, the title compound **5h** was obtained as an orange solid in 38% yield (34 mg, 0.074 mmol). *R*_f_ 0.80 (CH_2_Cl_2_/EtOAc 15:1). Mp. 256–259 °C. ^1^H NMR (300 MHz, CDCl_3_) δ = 8.83 (s, 1H), 8.26 (d, *J* = 4.3 Hz, 1H), 7.92–7.85 (m, 2H), 7.69 (ddd, *J* = 6.8 Hz, *J* = 1.5 Hz, *J* = 1.0 Hz, 1H), 7.65–7.55 (m, 7H), 7.50 (ddd, *J* = 8.7 Hz, *J* = 1.5 Hz, *J* = 0.7 Hz, 1H), 7.43 (d, *J* = 4.3 Hz, 1H), 7.38–7.32 (m, 3H), 3.0 (s, 3H). ^13^C NMR (75 MHz, CDCl_3_) δ = 153.4, 150.1, 148.8, 146.9, 146.8, 143.4, 140.9, 139.1, 137.4, 136.9, 131.3, 129.4, 129.1, 128.7, 128.7, 128.1, 127.4, 127.1, 126.9, 125.8, 125.8, 122.2, 117.2, 116.4, 111.8, 109.0, 18.2. IR (ATR, cm^−1^): ṽ = 1601 (m),1552 (m), 1447 (m), 1428 (s), 1339 (m), 1323 (m), 781 (s), 762 (vs), 701 (vs), 604 (m), 567 (m), 556 (s). MS (EI, 70 eV): *m*/*z* (%) = 460 (81, M^+^), 459 (100), 458 (6), 457 (6), 445 (7), 230 (16), 229 (18), 228 (8), 222 (10), 214 (8). HRMS (ESI-TOF): calculated for C_32_H_21_N_4_ ([M + H]^+^) 461.1766, found 461.1775.
*10,12-dimethyl-5,13-diphenylpyrimido[4′,5′,6′:9,1]pyrrolo[2′,1′,5′:4,5,6]quinolizino[3,2-b]quinoline* (**5i**)

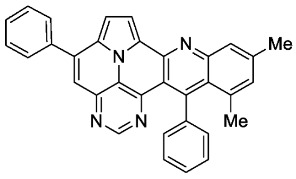

According to general procedure A, the title compound **5i** was obtained as an orange solid in 26% yield (34 mg, 0.072 mmol). *R*_f_ 0.82 (CH_2_Cl_2_/EtOAc 15:1). Mp. 317–320 °C. ^1^H NMR (500 MHz, CDCl_3_/TFA) δ = 9.12 (d, *J* = 4.9 Hz, 1H), 8.84 (s, 1H), 8.36 (s, 1H), 8.25 (s, 1H), 8.16 (d, *J* = 4.9 Hz, 1H), 7.95–7.87 (m, 2H), 7.76–7.64 (m, 6H), 7.58 (s, 1H), 7.40–7.35 (m, 2H), 2.77 (s, 3H), 2.15 (s, 3H). ^13^C NMR (126 MHz, CDCl_3_/TFA) δ = 165.2, 154.1, 147.5, 147.4, 145.6, 142.6, 142.0, 138.9, 137.5, 136.7, 135.2, 134.2, 132.1, 130.9, 130.4, 130.0, 129.4, 129.3, 127.1, 125.2, 121.3, 119.5, 119.2, 118.1, 117.3, 115.7, 112.8, 25.2, 22.8. IR (ATR, cm^−1^): ṽ = 1605 (s),1578 (m), 1550 (m), 1504 (m), 1451 (s), 1325 (m), 857 (m), 777 (s), 725 (s), 703 (vs), 558 (m). MS (EI, 70 eV): *m*/*z* (%) = 474 (89, M^+^), 473 (100), 471 (5), 470 (5), 399 (7), 237 (10), 236 (6), 229 (15), 228 (12), 214 (6). HRMS (ESI-TOF): calculated for C_33_H_22_N_4_ ([M + H]^+^) 475.1923, found 475.1934.
*11-fluoro-5,13-diphenylpyrimido[4′,5′,6′:9,1]pyrrolo[2′,1′,5′:4,5,6]quinolizino[3,2-b]quinoline* (**5j**)

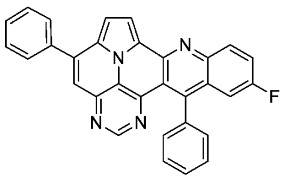

According to general procedure A, the title compound **5j** was obtained as an orange solid in 55% yield (69 mg, 0.148 mmol). *R*_f_ 0.79 (CH_2_Cl_2_/EtOAc 15:1). Mp. 348–350 °C. ^1^H NMR (500 MHz, CDCl_3_) δ = 8.85 (s, 1H), 8.32 (dd, *J* = 9.3 Hz, *J* = 5.5 Hz, 1H), 8.24 (d, *J* = 4.3 Hz, 1H), 7.91–7.86 (m, 2H), 7.67–7.55 (m, 8H), 7.44 (d, *J* = 4.3 Hz, 1H), 7.38–7.35 (m, 2H), 7.26 (dd, *J* = 10.3 Hz, *J* = 2.8 Hz, 1H). ^13^C NMR (126 MHz, CDCl_3_) δ = 160.1 (d, *J* = 248.7 Hz), 153.5, 149.5, 149.5, 147.0, 146.8, 146.4, 144.1 (d, *J* = 1.9 Hz), 141.1, 138.3, 137.3, 131.5 (d, *J* = 8.9 Hz), 129.5, 129.1, 128.7, 128.5, 128.4, 127.9 (d, *J* = 9.5 Hz), 127.8, 127.6, 126.2, 122.4 (d, *J* = 26.2 Hz), 117.5, 117.2, 112.0, 110.7 (d, *J* = 23.6 Hz), 109.2. ^19^F NMR (471 MHz, CDCl_3_) δ = −111.7. IR (ATR, cm^−1^): ṽ = 1488 (s),1175 (s), 830 (s), 777 (vs), 725 (s), 705 (vs), 593 (s), 552 (s), 474 (s), 460 (s), 443 (s). MS (EI, 70 eV): *m*/*z* (%) = 464 (89, M^+^), 463 (100), 462 (25), 435 (12), 232 (41), 231 (42), 230 (10), 218 (11), 217 (19). HRMS (ESI-TOF): calculated for C_31_H_18_FN_4_ ([M + H]^+^) 465.1516, found 465.1524.
*N,N-dimethyl-5,13-diphenylpyrimido[4′,5′,6′:9,1]pyrrolo[2′,1′,5′:4,5,6]quinolizino[3,2-b]quinolin-11-amine* (**5k**)

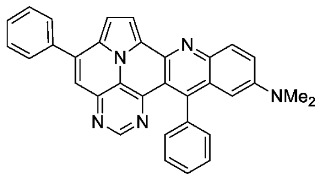

According to general procedure A, the title compound **5k** was obtained as an orange solid in 8% yield (11 mg, 0.021 mmol). *R*_f_ 0.82 (CH_2_Cl_2_/EtOAc 15:1). Mp. 130–134 °C. ^1^H NMR (500 MHz, CDCl_3_) δ = 8.81 (s, 1H), 8.25 (s, 2H), 7.91–7.88 (m, 2H), 7.62–7.57 (m, 5H), 7.57–7.55 (m, 3H), 7.45 (d, *J* = 4.3 Hz, 1H), 7.41–7.35 (m, 2H), 6.55 (d, *J* = 2.8 Hz, 1H), 2.96 (s, 6H). ^13^C NMR (126 MHz, CDCl_3_/TFA) δ = 148.3, 146.8, 146.7, 145.8, 138.9, 137.4, 134.9, 134.2, 134.0, 132.2, 130.8, 130.3, 130.1, 130.0, 129.8, 129.6, 129.3, 126.8, 122.4, 121.5, 120.4, 118.8, 117.8, 116.6, 112.0, 42.9. IR (ATR, cm^−1^): ṽ = 2920 (s),1607 (s), 1492 (s), 1449 (s), 1323 (s), 1123 (s), 775 (s), 699 (vs), 591 (s), 554 (s).MS (EI, 70 eV): *m*/*z* (%) = 489 (40, M^+^), 488 (24), 207 (14), 57 (25), 55 (20), 44 (100), 43 (28), 41 (23). HRMS (ESI-TOF): calculated for C_33_H_24_N_5_ ([M + H]^+^) 490.3032, found 490.2031.
*5,13-diphenyl-11-(trifluoromethyl)pyrimido[4′,5′,6′:9,1]pyrrolo[2′,1′,5′:4,5,6]quinolizino[3,2-b]quinoline* (**5l**)

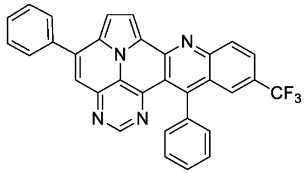

According to general procedure A, the title compound **5l** was obtained as an orange solid in 28% yield (38 mg, 0.075 mmol). *R*_f_ 0.85 (CH_2_Cl_2_/EtOAc 15:1). Mp. 311–313 °C. ^1^H NMR (500 MHz, CDCl_3_/TFA) δ = 9.10 (d, *J* = 4.9 Hz, 1H), 8.96 (s, 1H), 8.77 (d, *J* = 8.9 Hz, 1H), 8.56 (dd, *J* = 9.1 Hz, *J* = 1.8 Hz, 1H), 8.37 (s, 1H), 8.31 (s, 1H), 8.28 (d, *J* = 4.9 Hz, 1H), 7.95–7.89 (m, 2H), 7.86–7.82 (m, 1H), 7.81–7.71 (m, 5H), 7.41–7.37 (m, 2H). ^13^C NMR (126 MHz, CDCl_3_/TFA) δ = 167.4, 149.3, 147.4, 145.8, 141.4, 138.6, 137.3, 135.9 (q, *J* = 3.1 Hz), 133.8, 133.8, 132.7, 132.2, 132.1 (q, *J* = 34.7 Hz), 131.2, 130.3, 129.8, 129.4, 128.2 (q, *J* = 4.1 Hz), 127.1, 127.0, 122.4 (q, *J* = 273.1 Hz), 121.9, 121.5, 120.6, 119.7, 118.8, 116.9, 113.0. ^19^F NMR (471 MHz, CDCl_3_/TFA) δ = −63.8. IR (ATR, cm^−1^): ṽ = 1609 (m), 1311 (s), 1298 (s), 1117 (vs), 1067 (s), 985 (m), 837 (s), 779 (s), 701 (vs), 589 (s), 556 (s). MS (EI, 70 eV): *m*/*z* (%) = 514 (86, M^+^), 513 (100), 512 (5), 485 (5), 445 (4), 257 (11), 256 (14), 223 (4), 222 (13), 221 (4), 208 (5), 207 (4). HRMS (ESI-TOF): calculated for C_32_H_18_F_3_N_4_ ([M + H]^+^) 515.1483, found 515.1495.

#### 3.2.2. General Procedure B for the Synthesis of Aryl(8-arylpyrimido[4,5,6-*ij*]pyrrolo[2,1,5-*de*]quinolizin-4-yl)methanone (**6a**–**f**)

In a pressure tube, 100 mg of **4a**–**f** and 20 eq. of *p*-TsOH∙H_2_O were dissolved in 4 mL of xylene. The pressure tube was sealed with a Teflon cap and the solution was stirred for 6 h at 120 °C. The reaction mixture was cooled to room temperature, quenched with saturated NaHCO_3_ solution and extracted three times with 50 mL CH_2_Cl_2_. The combined organic phases were dried over Na_2_SO_4_; the solvent was distilled off in vacuo. The residue was purified by column chromatography (heptane/EtOAc) to yield the desired products (**6a–f**).

*Phenyl(8-phenylpyrimido[4,5,6-ij]pyrrolo[2,1,5-de]quinolizin-4-yl)methanone* (**6a**)

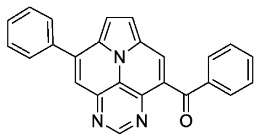

According to general procedure B, the title compound **6a** was obtained as a yellow solid in 68% yield (68 mg, 0.182 mmol). *R*_f_ 0.48 (heptane/EtOAc 1:2). Mp. 266–269 °C. ^1^H NMR (250 MHz, CDCl_3_) δ = 9.14 (s, 1H), 8.52 (s, 1H), 7.99–7.94 (m, 2H), 7.93 (s, 1H), 7.90–7.84 (m, 2H), 7.75–7.69 (m, 2H), 7.67–7.57 (m, 4H), 7.52–7.44 (m, 2H). ^13^C NMR (63 MHz, CDCl_3_) δ = 194.4, 155.1, 147.2, 144.9, 140.6, 137.2, 136.9, 133.5, 130.1, 129.6, 129.2, 128.9, 128.6, 128.1, 127.7, 126.3, 126.3, 124.7, 119.8, 113.9, 113.2. IR (ATR, cm^−1^): ṽ = 1644 (m), 1609 (s), 1455 (s), 1261 (s), 1238 (s), 1045 (s), 795 (s), 779 (s), 684 (vs), 637 (vs), 563 (vs). MS (EI, 70 eV): *m*/*z* (%) = 373 (16, M^+^), 372 (29), 346 (7), 345 (28), 344 (100), 316 (5), 240 (10), 187 (16), 172 (8). HRMS (ESI-TOF): calculated for C_25_H_15_N_3_O ([M + H]^+^) 374.1293, found 374.1294.
*p-tolyl(8-(p-tolyl)pyrimido[4,5,6-ij]pyrrolo[2,1,5-de]quinolizin-4-yl)methanone* (**6b**)

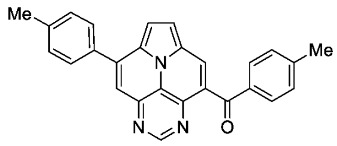

According to general procedure B, the title compound **6b** was obtained as a yellow solid in 54% yield (54 mg, 0.135 mmol). *R*_f_ 0.52 (heptane/EtOAc 1:2). Mp. 258–261 °C. ^1^H NMR (300 MHz, CDCl_3_) δ = 9.12 (s, 1H), 8.47 (s, 1H), 7.88 (s, 1H), 7.86 (d, *J* = 8.3 Hz, 2H), 7.76 (d, *J* = 8.1 Hz, 2H), 7.73–7.66 (m, 2H), 7.45–7.39 (m, 2H), 7.30–7.21 (m, 2H), 2.50 (s, 3H), 2.43 (s, 3H). ^13^C NMR (75 MHz, CDCl_3_) δ = 194.0, 155.1, 147.2, 144.8, 144.5, 140.5, 139.8, 134.6, 134.0, 130.3, 129.9, 129.2, 128.8, 128.4, 127.6, 126.2, 125.9, 124.6, 119.4, 113.6, 113.1, 21.7, 21.3. IR (ATR, cm^−1^): ṽ = 1607 (s), 1457 (s), 1344 (vs), 1267 (s), 1251 (s), 1043 (s), 903 (s), 824 (s), 777 (vs), 756 (s), 723 (s), 563 (vs). MS (EI, 70 eV): *m*/*z* (%) = 401 (19, M^+^), 400 (28), 373 (31), 372 (100), 371 (9), 201 (12), 186 (8), 179 (6), 178 (11), 91 (7). HRMS (ESI-TOF): calculated for C_27_H_19_N_3_O ([M + H]^+^) 402.1606, found 402.1602.
*(4-fluorophenyl)(8-(4-fluorophenyl)pyrimido[4,5,6-ij]pyrrolo[2,1,5-de]quinolizin-4-yl)methanone* (**6c**)

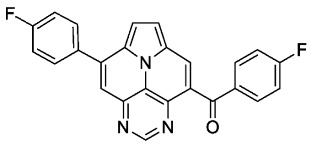

According to general procedure B, the title compound **6c** was obtained as a yellow solid in 71% yield (71 mg, 0.173 mmol). *R*_f_ 0.48 (heptane/EtOAc 1:2). Mp. 317–320 °C. ^1^H NMR (500 MHz, CDCl_3_/TFA) δ = 9.25 (s, 1H), 9.17 (s, 1H), 8.63 (s, 1H), 8.41 (d, *J* = 5.0 Hz, 1H), 8.35 (d, *J* = 5.0 Hz, 1H), 8.00–7.87 (m, 4H), 7.44 (pt, *J* = 8.3 Hz, 2H), 7.30 (pt, *J* = 8.3 Hz, 2H). ^13^C NMR (126 MHz, CDCl_3_/TFA) δ = 193.9, 166.6 (d, *J* = 258.6 Hz), 164.9 (d, *J* = 254.6 Hz), 146.3, 144.6, 143.6, 136.7, 132.8 (d, *J* = 9.7 Hz), 131.9 (d, *J* = 8.9 Hz), 131.7, 130.6, 130.4, 130.3, 129.9, 122.9, 121.9, 121.2, 118.2, 117.5 (d, *J* = 22.1 Hz), 116.7 (d, *J* = 22.3 Hz). ^19^F NMR (471 MHz, CDCl_3_/TFA) δ = −101.5, −107.3. IR (ATR, cm^−1^): ṽ = 1609 (s), 1599 (s), 1589 (s), 1508 (s), 1453 (s), 1267 (s), 1243 (vs), 1232 (s), 1158 (s), 847 (s), 837 (s), 783 (vs), 563 (s). MS (EI, 70 eV): *m*/*z* (%) = 409 (14, M^+^), 408 (17), 382 (4), 381 (27), 380 (100), 379 (7), 259 (4), 258 (10), 205 (7), 190 (4), 95 (9). HRMS (ESI-TOF): calculated for C_25_H_14_F_2_N_3_O ([M + H]^+^) 410.1105, found 410.1113.
*(4-(dimethylamino)phenyl)(8-(4-(dimethylamino)phenyl)pyrimido[4,5,6-ij]pyrrolo[2,1,5-de]quinolizin-4-yl)methanone* (**6d**)

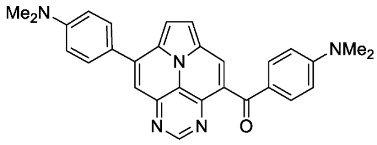

According to general procedure B, the title compound **6d** was obtained as a yellow solid in 54% yield (54 mg, 0.118 mmol). *R*_f_ 0.29 (EtOAc). Mp. 255–258 °C. ^1^H NMR (500 MHz, CDCl_3_) δ = 9.11 (s, 1H), 8.39 (s, 1H), 7.91–7.75 (m, 6H), 7.63 (d, *J* = 4.6 Hz, 1H), 6.94–6.88 (m, 2H), 6.67–6.61 (m, 2H), 3.09 (s, 6H), 3.07 (s, 6H). ^13^C NMR (126 MHz, CDCl_3_) δ = 192.2, 155.0, 153.9, 151.2, 147.2, 144.6, 140.8, 132.7, 130.0, 129.7, 127.4, 126.4, 124.8, 124.7, 124.3, 124.3, 117.5, 113.1, 112.8, 112.4, 110.6, 40.3, 40.0. IR (ATR, cm^−1^): ṽ = 1597 (vs), 1523 (s), 1346 (s), 1284 (s), 1271 (s), 1261 (s), 1189 (s), 1179 (s), 1168 (s), 818 (s), 779 (s), 771 (s), 560 (s). MS (EI, 70 eV): *m*/*z* (%) = 459 (53, M^+^), 458 (39), 445 (22), 444 (28), 431 (34), 430 (100), 416 (35), 414 (16), 230 (18), 215 (13). HRMS (ESI-TOF): calculated for C_29_H_26_N_5_O ([M + H]^+^) 460.2137, found 460.2148.
*(4-methoxyphenyl)(8-(4-methoxyphenyl)pyrimido[4,5,6-ij]pyrrolo[2,1,5-de]quinolizin-4-yl)methanone* (**6e**)

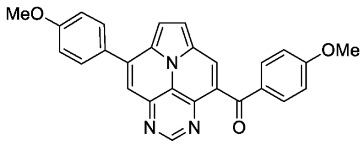

According to general procedure B, the title compound **6e** was obtained as a yellow solid in 51% yield (51 mg, 0.118 mmol). *R*_f_ 0.29 (heptane/EtOAc 1:3). Mp. 265–267 °C. ^1^H NMR (300 MHz, CDCl_3_) δ = 9.27 (s, 1H), 9.14 (s, 1H), 8.64 (s, 1H), 8.46–8.40 (m, 2H), 7.96–7.84 (m, 4H), 7.26 (d, *J* = 8.9 Hz, 2H), 7.10 (d, *J* = 8.3 Hz, 2H), 3.99 (s, 3H), 3.95 (s, 3H). ^13^C NMR (75 MHz, CDCl_3_) δ = 194.3, 165.4, 162.7, 146.3, 145.8, 143.0, 135.7, 133.1, 131.8, 131.7, 130.5, 130.1, 127.8, 126.6, 123.1, 122.9, 122.0, 121.1, 118.1, 115.8, 114.9, 55.8, 55.7. IR (ATR, cm^−1^): ṽ = 1597 (s), 1455 (s), 1253 (vs), 1166 (vs), 1041 (s), 1020 (s), 832 (s), 775 (s), 604 (s), 575 (s), 560 (s), 536 (s). MS (EI, 70 eV): *m*/*z* (%) = 433 (16, M^+^), 432 (20), 405 (26), 404 (100), 389 (11), 361 (13), 217 (26), 180 (9), 265 (7). HRMS (ESI-TOF): calculated for C_27_H_20_N_3_O_3_ ([M + H]^+^) 434.1505, found 434.1518.
*(4-(trifluoromethyl)phenyl)(8-(4-(trifluoromethyl)phenyl)pyrimido[4,5,6-ij]pyrrolo[2,1,5-de]quinolizin-4-yl)methanone* (**6f**)

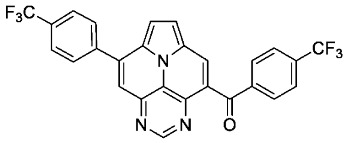

According to general procedure B, the title compound **6f** was obtained as a yellow solid in 62% yield (62 mg, 0.121 mmol). *R*_f_ 0.52 (heptane/EtOAc 1:2). Mp. 309–311 °C. ^1^H NMR (500 MHz, CDCl_3_/TFA) δ = 9.23 (s, 1H), 9.18 (s, 1H), 8.66 (s, 1H), 8.41 (d, *J* = 5.0 Hz, 1H), 8.30 (d, *J* = 5.0 Hz, 1H), 8.04 (d, *J* = 8.1 Hz, 2H), 8.02–7.94 (m, 4H), 7.85 (d, *J* = 7.9 Hz, 2H). ^13^C NMR (126 MHz, CDCl_3_) δ = 194.3, 146.2, 144.1, 143.8, 138.8, 137.6, 137.0, 135.3 (q, *J* = 33.0 Hz), 133.4 (q, *J* = 33.3 Hz), 131.7, 130.9, 130.2, 130.0, 129.9, 126.9 (q, *J* = 3.8 Hz), 126.2 (q, *J* = 3.8 Hz), 123.6, 123.5 (q, *J* = 272.7 Hz), 123.3, 123.3 (q, *J* = 272.9 Hz), 122.6, 120.8, 117.8. ^19^F NMR (471 MHz, CDCl_3_) δ = −63.2, −63.5. IR (ATR, cm^−1^): ṽ = 1325 (vs), 1166 (s), 1109 (vs), 1067 (s), 1057 (s), 1049 (s), 1018 (s), 845 (m), 787 (s), 670 (m), 563 (m). MS (EI, 70 eV): *m*/*z* (%) = 509 (12, M^+^), 508 (20), 482 (5), 481 (28), 480 (100), 255 (7), 240 (8), 145 (9). HRMS (ESI-TOF): calculated for C_27_H_14_F_6_N_3_O ([M + H]^+^) 510.1041, found 510.1055.

## Figures and Tables

**Table 1:** Optimization of one-pot reaction for the synthesis of **5a** consisting of the Schiff base formation, Povarov reaction and cycloisomerization.

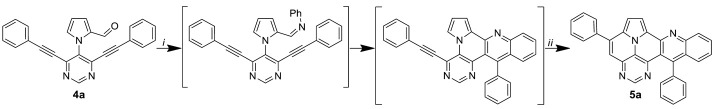							
Entry	eq. FeCl_3_	Acid (eq.)	Solvent	*T* [°C]	*t*_1_ [h]	*t*_2_ [h]	Yield ^[a]^ [%]
1	0.1	*p*-TsOH∙H_2_O (30)	toluene	100	3	2	traces
2	0.3	*p*-TsOH∙H_2_O (30)	xylene	140	3	2	29
3	0.6	*p*-TsOH∙H_2_O (30)	xylene	140	3	2	34
4	1	*p*-TsOH∙H_2_O (30)	xylene	140	3	2	38
5	4	-	xylene	140	3	-	21
6	1	*p*-TsOH∙H_2_O (10)	xylene	140	3	2	23
7	1	*p*-TsOH∙H_2_O (20)	xylene	140	3	2	27
**8**	**1**	***p*-TsOH∙H_2_O (30)**	**xylene**	**140**	**3**	**6**	**45**
9	1	*p*-TsOH∙H_2_O (30)	xylene	140	3	16	45
10	1	*p*-TsOH∙H_2_O (40)	xylene	140	3	6	43
11	1	MsOH (30)	xylene	140	3	6	40

*i*: aniline (1.2 eq.), FeCl_3_, *T*, *t*_1_. *ii*: acid, *T*, *t*_2_. ^[a]^ isolated yield (bold highlighted line indicates used conditions for scope analysis).

**Table 2:** Optimization of cycloisomerization and ACM reaction for **6a**.

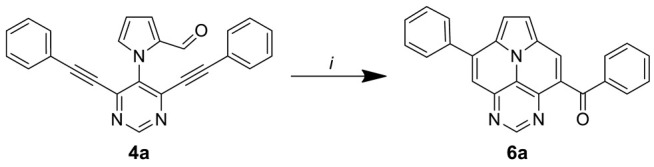					
Entry	Acid (eq.)	Solvent	*T* [°C]	*t* [h]	Yield ^[a]^ [%]
1	*p*-TsOH∙H_2_O (30)	-	120	16	57
2	*p*-TsOH∙H_2_O (30)	xylene	120	16	68
3	*p*-TsOH∙H_2_O (30)	xylene	140	16	59
4	*p*-TsOH∙H_2_O (20)	xylene	120	16	67
5	*p*-TsOH∙H_2_O (10)	xylene	120	16	53
6	MsOH (20)	xylene	120	16	27
**7**	***p*-TsOH∙H_2_O (20)**	**xylene**	**120**	**6**	**68**

*i*: acid, solvent, *T*, *t*. ^[a]^ isolated yield (bold highlighted line indicates used conditions for scope analysis).

**Table 3:** Comparison of properties with related molecular structures.

	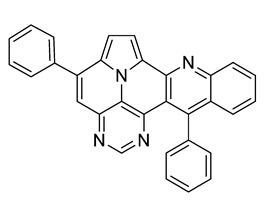	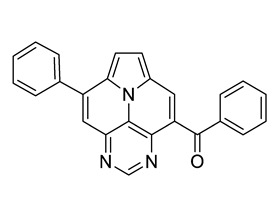	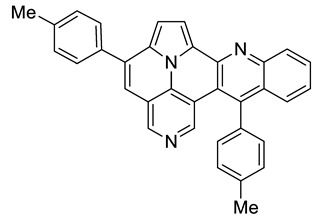	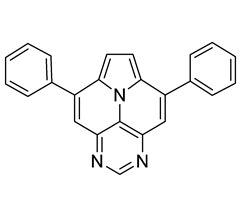
	5a	6a	7 [30]	8 [29]
λ_1,abs_ [nm]	497	420	471	422
ε_λ1_ [10^4^ L·mol^−1^cm^−1^]	0.8	2.0	1.1	1.7
λ_1,em_ [nm]	530	462	515	439
Φ	0.52	0.10	0.26	0.35
E_Ox._ [V vs. Fc/Fc^+^]	0.95	1.08	0.72	1.00
E_HOMO/CV_ [eV]	−5.65	−5.76	−5.41	−5.69
E_HOMO/DFT_ [eV]	−5.53	−5.73	−5.22	−5.60
E_LUMO/DFT_ [eV]	−2.43	−2.04	−2.09	−1.90
E_gap_ [eV]	3.10	3.69	3.13	3.69

## Data Availability

The data underlying this study are available in this article and its Supplementary Materials.

## Supplementary Materials

The following supporting information can be downloaded at: https://www.mdpi.com/article/10.3390/molecules29092159/s1, containing analytical data of starting materials, X-ray chrystallograpfic, UV-Vis data (solvatochromism), Cartesian Coordinates from DFT calculations and NMR-spectra of final products. Reference [44] is cited in the Supplementary Materials.
