# Supplementary material for: Divergent Synthesis of 5,7-Diazaullazines Derivatives through a Combination of Cycloisomerization with Povarov or Alkyne–Carbonyl Metathesis
Source: Molecules. 2024 May 6;29(9):2159. doi: 10.3390/molecules29092159 (PMC11085690; doi:10.3390/molecules29092159)
Supplement: Supplementary file 1 [file molecules-29-02159-s001.zip › molecules-2901208-supplementary.pdf]

Supplementary Materials

Divergent Synthesis of 5,7-Diazaullazines Derivatives Through a Combination of Cycloisomerization with Povarov or Alkyne–Carbonyl Metathesis

Jonas Polkaehn [1], Peter Ehlers [1,*], Alexander Villinger [1] and Peter Langer [1,2,*]

[1] Institute of Chemistry, University Rostock, Albert-Einstein-Str. 3a, 18059 Rostock, Germany.

E-mail: peter.langer@uni-rostock.de; Fax: +49 381 498 6412; Tel: +49 381 498 6410

[2] Leibniz Institute for Catalysis (LIKAT) at the University Rostock, Albert-Einstein-Str. 29a, 18059 Rostock, Germany.

Table of Contents

Analytical Data 2

X-Ray 5

UV-vis-Data 6

Solvatochromism 6

DFT Calculations 8

Cartesian coordinates of the optimized ground-states (S_0) and excited-states (S_1) 8

1H-, 13C- and 19F-NMR Spectra 19

REFERENCES 46

Analytical Data

4,6-dichloro-5-(1*H*-pyrrol-1-yl)pyrimidine (2)

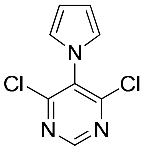

4,6-dichloropyrimidin-5-amine (**1**, 5.00 g, 30.49 mmol) was dissolved in 25 ml of acetic acid and 25 ml of 1,2-dichloroethane. Then 2,5-dimethoxy-tetrahydrofuran (1.05 eq.) was added. The solution was heated for 3 h under reflux. After cooling to room temperature, the reaction solution was extracted three times with dichloromethane. The combined organic phases were dried over Na_2SO_4, the solvent was distilled off in vacuo, and the residue was purified by column chromatography (heptane/EtOAc 5:1) to give **2** as a colorless solid in 95% yield (6.20 g, 28.97 mmol). The NMR data agree with previously published NMR data [1]. ^{1}H NMR (300 MHz, $CDCl_3$) δ = 8.79 (s, 1H), 6.75 – 6.72 (m, 2H), 6.45 – 6.43 (m, 2H). ^{13}C NMR (75 MHz, $CDCl_3$) δ = 160.2, 156.3, 132.7, 121.5, 111.0.

1-(4,6-dichloropyrimidin-5-yl)-1*H*-pyrrole-2-carbaldehyde (3)

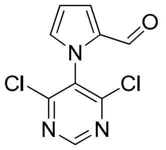

4,6-dichloro-5-(1*H*-pyrrol-1-yl)pyrimidine (**2**, 1.00 g, 4.67 mmol) was suspended in 5 ml DMF in a Schlenk flask under an argon atmosphere. 2 eq. $POCl_3$ were added dropwise at 0° C. The solution was stirred at 100° C for 3 h, cooled to room temperature, neutralized with saturated $NaHCO_3$-solution and extracted with DCM. The combined organic phases were dried with Na_2SO_4, the solvent was removed in vacuo and the residue was purified by column chromatography to give **3** as a colorless solid in 41% yield (460 mg, 1.90 mmol). Mp. 86-88 °C. ^{1}H NMR (250 MHz, $CDCl_3$) δ = 9.58 (d, J = 1.1 Hz, 1H), 8.83 (s, 1H), 7.19 (dd, J = 4.0 Hz, J = 1.5 Hz, 1H), 6.94 (ddd, J = 2.7 Hz, J = 1.5 Hz, J = 1.1 Hz, 1H), 6.58 (dd, J = 4.0 Hz, J = 2.8 Hz, 1H, CH). ^{13}C NMR (63 MHz, $CDCl_3$) δ = 178.6, 160.1, 156.8, 132.1, 131.9, 129.8, 124.4, 112.8. IR (ATR, cm^{-1}): ṽ = 1653 (s), 1517 (s), 1469 (s), 1416 (s), 1383 (s), 1362 (s), 1348 (s), 1084 (m), 1030 (m), 810 (s), 742 (vs). MS (EI, 70 eV): m/z (%) = 241 (6, M^+), 240 (3), 209 (3), 208 (32), 207 (11), 206 (100), 124 (4), 93 (4), 93 (10), 65 (3), 39 (3). HRMS (ESI-TOF): calculated for $C_9H_6Cl_2N_3O$ ($[M+H]^+$) 241.9888, found 241.9892.

General Procedure C for the synthesis of 1-(4,6-bis(arylethynyl)pyrimidin-5-yl)-1*H*-pyrrole-2-carbaldehyde (4a-f)

In a pressure tube, 250 mg (1.04 mmol) of 1-(4,6-dichloropyrimidin-5-yl)-1*H*-pyrrole-2-carbaldehyde (**3**), 0.06 eq. $PdCl_2(CH_3CN)_2$, 0.04 eq. CuI, 0.12 eq. XPhos were dissolved in 1 ml HN^iPr_2 and 2 mL of 1,4-dioxane under argon counter current. Then, 3 eq. of the respective alkyne was added to the solution with stirring. The pressure tube was sealed with a Teflon cap and the solution was stirred for 24 h at 90 °C. The reaction mixture was cooled to room temperature, quenched with distilled water and extracted three times with DCM. The combined organic phases were dried over Na_2SO_4, the solvent was distilled off in vacuo, and the residue was purified by column chromatography (heptane/EtOAc) to give the desired products (**4a-f**).

1-(4,6-bis(phenylethynyl)pyrimidin-5-yl)-1*H*-pyrrole-2-carbaldehyde (4a)

According to general procedure C, the title compound **4a** was obtained as a yellow-orange solid in 79% yield (306 mg, 0.82 mmol). R_f 0.33 (heptane/EtOAc 2:1). Mp. 121-124 °C. ^{1}H NMR (300 MHz, $CDCl_3$) δ = 9.62 (d,

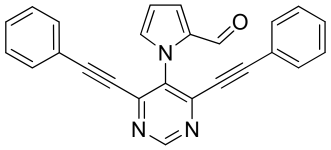

J = 0.9 Hz, 1H), 9.18 (s, 1H), 7.43 – 7.30 (m, 10H), 7.28 (dd, J = 4.0 Hz, J = 1.6 Hz, 1H), 7.18 (ddd, J = 2.6 Hz, J = 1.6 Hz, J = 1.0 Hz, 1H), 6.64 (dd, J = 4.0 Hz, J = 2.7 Hz, 1H). ^{13}C NMR (75 MHz, $CDCl_3$) δ = 178.3, 157.9, 148.9, 136.9, 133.1, 132.5, 131.1, 130.4, 128.5, 123.3, 120.5, 111.7, 99.4, 83.3. IR (ATR, cm^{-1}): ṽ = 2211 (m), 1659 (s), 1543 (s), 1469 (s), 1420 (s), 1401 (s), 1360 (m), 964 (m), 746 (vs), 684 (s), 530 (s). MS (EI, 70 eV): m/z (%) = 373 (8, M^+), 372 (30), 346 (14), 345 (70), 344 (100), 343 (17), 342 (9), 318 (9), 317 (11), 316 (16), 315 (6), 172 (7), 158 (7). HRMS (ESI-TOF): calculated for $C_{25}H_{16}N_3O$ ($[M+H]^+$) 374.1293, found 374.1291.

1-(4,6-bis(*p*-tolylethynyl)pyrimidin-5-yl)-1*H*-pyrrole-2-carbaldehyde (4b)

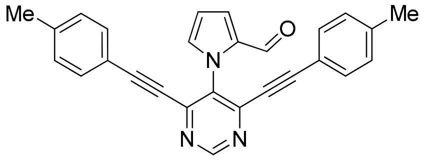

According to general procedure C, the title compound **4b** was obtained as a yellow-orange solid in 75% yield (312 mg, 0.78 mmol). R_f 0.41 (heptane/EtOAc 2:1). Mp. 127-129°C. ^{1}H NMR (250 MHz, $CDCl_3$) δ = 9.62 – 9.59 (m, 1H), 9.15 (s, 1H), 7.26 (dd, J = 3.5 Hz, J = 2.0 Hz, 1H), 7.22 (d, J = 8.1 Hz, 4H), 7.18 – 7.16 (m, 1H), 7.12 (d, J = 8.2 Hz, 4H), 6.62 (dd, J = 4.0 Hz, J = 2.7 Hz, 1H), 2.35 (s, 6H). ^{13}C NMR (63 MHz, $CDCl_3$) δ = 178.2, 157.9, 149.0, 141.0, 136.6, 133.1, 132.5, 131.1, 129.3, 123.1, 117.5, 111.6, 100.0, 83.1, 21.7. IR (ATR, cm^{-1}): ṽ = 2211 (s),1663 (s), 1541 (s), 1469 (s), 1422 (s), 1403 (s), 814 (s), 760 (vs), 742 (s), 530 (s). MS (EI, 70 eV): m/z (%) = 401 (8, M^+), 400 (25), 374 (21), 373 (85), 372 (100), 371 (9), 357 (11), 356 (8), 178 (12), 164 (8). HRMS (ESI-TOF): calculated for $C_{27}H_{20}N_3O$ ($[M+H]^+$) 402.1606, found 402.1613.

1-(4,6-bis((4-fluorophenyl)ethynyl)pyrimidin-5-yl)-1*H*-pyrrole-2-carbaldehyde (4c)

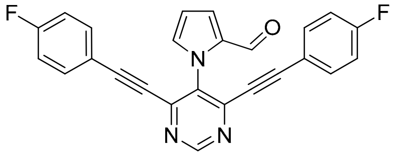

According to general procedure C, the title compound **4c** was obtained as a yellow-orange solid in 74% yield (315 mg, 0.77 mmol). R_f 0.25 (heptane/EtOAc 3:1). Mp. 161-165°C. ^{1}H NMR (300 MHz, $CDCl_3$) δ = 9.61 (d, J = 1.0 Hz, 1H), 9.16 (s, 1H), 7.34 – 7.28 (m, 4H), 7.28 – 7.26 (m, 1H), 7.17 (ddd, J = 2.6 Hz, J = 1.6 Hz, J = 1.0 Hz, 1H), 7.05 – 6.97 (m, 4H), 6.63 (dd, J = 3.9 Hz, J = 2.7 Hz, 1H). ^{13}C NMR (75 MHz, $CDCl_3$) δ = 178.2, 163.7 (d, J = 253.5 Hz), 157.9, 148.8, 136.7, 134.7 (d, ^3J = 8.9 Hz), 133.1, 131.0, 123.2, 116.6 (d, J = 3.5 Hz), 116.0 (d, J = 22.4 Hz), 111.7, 98.3, 83.1 (d, J = 1.6 Hz). ^{19}F NMR (282 MHz, $CDCl_3$) δ = -106.6. IR (ATR, cm^{-1}): ṽ = 2209 (s),1663 (s), 1541 (s), 1529 (s), 1506 (vs), 1224 (s), 1216 (s), 1154 (s), 966 (s), 830 (vs), 762 (vs), 501 (vs). MS (EI, 70 eV): m/z (%) = 409 (7, M^+), 408 (27), 382 (18), 381 (84), 380 (100), 379 (16), 378 (7), 354 (10), 353 (13), 352 (18), 176 (7), 157 (8), 144 (8). HRMS (ESI-TOF): calculated for $C_{25}H_{14}F_2N_3O$ ($[M+H]^+$) 410.1105, found 410.1103.

1-(4,6-Bis((4-(dimethylamino)phenyl)ethinyl)pyrimidin-5-yl)-1*H*-pyrrol-2-carbaldehyd (4d)

According to general procedure C, the title compound **4d** was obtained as a yellow-orange solid in 77% yield (367 mg, 0.80 mmol). R_f 0.41 (heptane/EtOAc 1:1). Mp. 137-140 °C. ^{1}H NMR (300 MHz, $CDCl_3$) δ = 9.58 (d, J = 0.8 Hz, 1H), 9.03 (s, 1H), 7.26 (dd, J = 4.0 Hz, J = 1.6 Hz, 1H), 7.20 – 7.14 (m, 5H), 6.60 (dd, J = 4.0 Hz,

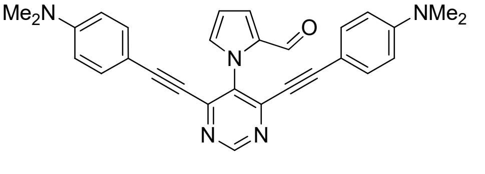

J = 2.7 Hz, 1H), 6.58 – 6.53 (m, 4H), 2.99 (s, 12H). ^{13}C NMR (75 MHz, $CDCl_3$) δ = 178.2, 157.8, 151.3, 149.1, 134.9, 134.1, 133.1, 131.1, 122.0, 111.4, 111.2, 106.7, 102.8, 83.2, 40.0. IR (ATR, cm^{-1}): ṽ = 2174 (m),1665 (s), 1601 (s), 1521 (vs), 1356 (vs), 1160 (s), 814 (vs), 758 (vs), 746 (vs), 519 (vs). MS (EI, 70 eV): m/z (%) = 459 (17, M$^+$), 432 (14), 431 (37), 430 (32), 281 (39), 215 (21), 209 (13), 208 (19), 207 (100). HRMS (ESI-TOF): calculated for $C_{29}H_{26}N_5O$ ([M+H]$^+$) 460.2137, found 460.2145.

1-(4,6-bis((4-methoxyphenyl)ethynyl)pyrimidin-5-yl)-1*H*-pyrrole-2-carbaldehyde (4e)

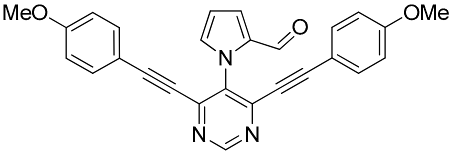

According to general procedure C, the title compound **4e** was obtained as a yellow-orange solid in 77% yield (347 mg, 0.80 mmol). R_f 0.36 (heptane/EtOAc 1:1). Mp. 152-154 °C. ^{1}H NMR (300 MHz, $CDCl_3$) δ = 9.60 (d, J = 0.9 Hz, 1H), 9.11 (s, 1H), 7.31 – 7.20 (m, 5H), 7.16 (ddd, J = 2.6 Hz, J = 1.6 Hz, J = 0.9 Hz, 1H), 6.85 – 6.80 (m, 4H), 6.62 (dd, J = 3.9 Hz, J = 2.7 Hz, 1H), 3.80 (s, 6H). ^{13}C NMR (75 MHz, $CDCl_3$) δ = 178.2, 161.3, 157.8, 149.0, 136.1, 134.3, 133.1, 131.1, 122.8, 114.2, 112.5, 111.5, 100.3, 82.9, 55.3. IR (ATR, cm^{-1}): ṽ = 2201 (s),1661 (s), 1541 (s), 1506 (s), 1465 (s), 1253 (vs), 1170 (s), 1160 (s), 1028 (s), 826 (vs), 760 (vs), 746 (vs), 538 (vs). MS (EI, 70 eV): m/z (%) = 433 (5, M$^+$), 432 (16), 406 (27), 405 (100), 404 (62), 390 (8), 389 (7), 362 (5), 361 (6), 319 (5), 318 (5), 159 (6). HRMS (ESI-TOF): calculated for $C_{27}H_{20}N_3O_3$ ([M+H]$^+$) 434.1505, found 434.1509.

1-(4,6-bis((4-(trifluoromethyl)phenyl)ethynyl)pyrimidin-5-yl)-1*H*-pyrrole-2-carbaldehyde (4f)

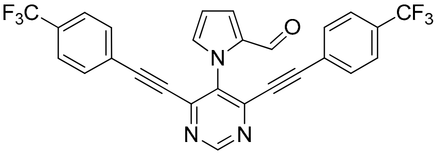

According to general procedure C, the title compound **4e** was obtained as a yellow-orange solid in 63% yield (336 mg, 0.66 mmol). R_f 0.41 (heptane/EtOAc 3:1). Mp. 140-143 °C. ^{1}H NMR (300 MHz, $CDCl_3$) δ = 9.63 (d, J = 1.0 Hz, 1H), 9.23 (s, 1H), 7.61 – 7.56 (m, 4H), 7.46 – 7.41 (m, 4H), 7.28 (dd, J = 4.0 Hz, J = 1.6 Hz, 1H), 7.19 (ddd, J = 2.6 Hz, ^4J = 1.6 Hz, J = 1.0 Hz, 1H), 6.66 (dd, J = 4.0 Hz, J = 2.7 Hz, 1H). ^{13}C NMR (75 MHz, $CDCl_3$) δ = 178.3, 158.0, 148.6, 137.5, 133.1, 132.7, 132.0 (q, J = 32.9 Hz, C), 131.0, 125.5 (q, J = 3.8 Hz), 124.2 (q, J = 1.7 Hz), 123.6, 123.5 (q, J = 272.6 Hz), 111.9, 97.2, 84.8. ^{19}F NMR (282 MHz, $CDCl_3$) δ = -63.18. IR (ATR, cm^{-1}): ṽ = 2219 (m),1665 (s), 1539 (m), 1319 (vs), 1164 (s), 1121 (vs), 1105 (s), 1063 (vs), 1016 (s), 839 (s), 758 (s), 744 (m), 723 (m). MS (EI, 70 eV): m/z (%) = 509 (13, M$^+$), 508 (37), 482 (25), 481 (100), 480 (98), 479 (6), 454 (8), 412 (6), 411 (8). HRMS (ESI-TOF): calculated for $C_{27}H_{14}F_6N_3O$ ([M+H]$^+$) 510.1041, found 510.1049.

X-Ray

*Table S1: 5,13-bis(4-fluorophenyl)pyrimido[4',5',6':9,1]pyrrolo[2',1',5':4,5,6]quinolizino[3,2-b]quinoline (**5d**)*

Chem. Formula	$C_{31}H_{16}N_4F_2$ + 2 $CHCl_3$
Form. Wght [g mol^{-1}]	721.21
color	orange
Crsyt. system	triclinic
Space group (Hall group)	P -1 (-P 1)
a [Å]	10.2831(8)
b [Å]	12.1814(10)
c [Å]	12.2969(11)
α [°]	88.868(3)
β [°]	78.979(3)
γ [°]	86.263(3)
V [Å^3]	1508.7(2)
Z	2
N_{ref}	10029
θ_{max} [°]	31.499
h,k,l_{max}	15,17,18
ρ_x[g cm^{-3}]	1.588
μ [mm^{-1}]	0.615
$\lambda_{MoK\backslash\alpha}$ [A]	0.71073
T [K]	123
F(000)	728.0
N_{par}	438
R	0.0357(8508)
wR_2	0.1020(10029)
S	1.047

UV-vis-Data

*Table S2: detailed Spectroscopic Data of **5a**, **5k**, **5l** and **6a** in DCM (c = 10^{-5} M) at 20 °C*

	**5a**	**5k**	**5l**	**6a**
$\lambda_{1,abs}$ [nm]	497	516	503	420
$\varepsilon_{\lambda 1}$	0.8	0.3	0.7	2.0
$\lambda_{2,abs}$ [nm]	479	378[b]	475	399[b]
$\varepsilon_{\lambda 2}$	0.8	1.0	0.7	1.2
$\lambda_{3,abs}$ [nm]	395[b]	347	402[b]	
$\varepsilon_{\lambda 3}$	0.7	2.2	0.4	
$\lambda_{4,abs}$ [nm]	370[b]		378[b]	
$\varepsilon_{\lambda 4}$	1.4		1.4	
$\lambda_{5,abs}$ [nm]	339		348	
$\varepsilon_{\lambda 5}$	4.8		5.4	
$\lambda_{1,em}$ [nm]	530	548[b]	542	462
$\Lambda_{2,em}$ [nm]		585		
$E_g^{opt,c}$[eV]	2.47	2.42	2.43	2.85
Φ	0.52[d]	0.29[d]	0.53[d]	0.10[e]

[a] 10^4 L·mol^{-1}cm^{-1}; [b] indicated as shoulder; [c] determined from the intersection of the normalized absorption and emission spectra; [d] Fluorescence standard: rhodamine 6G in EtOH (Φ = 0.94) [2].; [e] Fluorescence standard: coumarin 153 in EtOH (Φ = 0.38) [2].

Solvatochromism

Although there is hardly any difference in the absorption spectra for the two compounds in the different solvents, **5a** nevertheless displays a decrease in the fine structure with an increased polarity. In the emission, stronger influences are perceptible. In addition, both compounds possess a different behavior, **5a** displays a change in structure, while in toluene and DCM an emission peak with a shoulder can be seen, in acetonitrile and ethanol this shoulder disappears. Furthermore, a slight redshift is apparent. **5k**, on the other hand, exhibits a stronger broadening of the emission with higher polarity. Interestingly, the maximum of the emission is red shifted from toluene to DCM, but blue shifted in acetonitrile and even more in ethanol. In addition, we determined the quantum yields in the different solvents. For **5a**, there is hardly any change in the quantum yields (52%-56%). For **5c**, however, the quantum yield decreases with increasing polarity of the solvent. In toluene the yield is 32%, in DCM at 29% while in acetonitrile and ethanol it is only 20%. The data are specified in Table S3.

Furthermore, we calculated the dipole moments of the transition state. Both compounds show only a slight increase. However, **5k** (s_0 = 5.29 D; s_1 = 6.06 D) already has a higher dipole moment in the ground state than **5a** (s_0 = 1.54 D; s_1 = 2.00 D). All in all, both molecules exhibit only a very weak ICT character, which is somewhat reinforced by the NMe_2-group in **5k**.

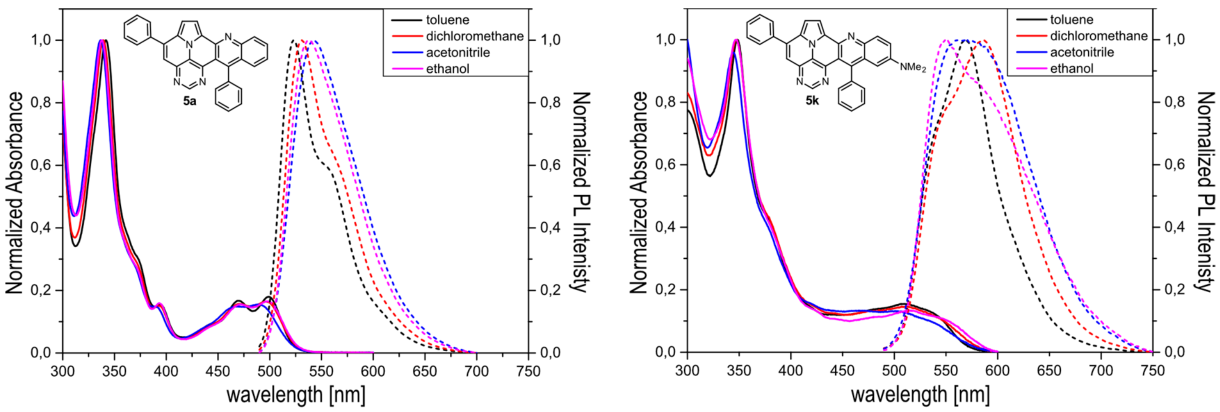

*Figure S1: solvatochromism studies of **5a** and **5k***

*Table S3: Quantuum yields and $\lambda_{max,em}$ of solvatochromism measurements for **5a** and **5k***

	toluene		DCM		Acetonitrile		Ethanol	
	Φ[a]	$\lambda_{max,em}$ [nm]	Φ[a]	$\lambda_{max\ em}$ [nm]	Φ[a]	$\lambda_{max,em}$ [nm]	Φ[a]	$\lambda_{max,em}$ [nm]
**5a**	0.56	523	0.52	530	0.54	542	0.54	537
**5k**	0.32	568	0.29	585	0.20	568	0.20	551

[a:] Fluorescence standard: rhodamine 6G in EtOH (Φ = 0.94) [2].

DFT Calculations

Density functional theory (DFT) and time-dependent density functional theory (TD-DFT) calculations were performed with Gaussian09 [3]. The ground and excited state structures were optimized using the B3LYP, functional and the 6-31G(d,p) basis set. The solvent effects have been considered by using the integral equation formalism variant (IEFPCM) model. NICS2BC were calculated with the B3LYP functional coupled with Grimme's D3 empirical dispersion correction and 6-311G(d,p) basis set. Nucleus independent chemical shifts (NICS) were calculated using the gauge including atomic orbitals (GIAO) method at the same level of theory. The bond current maps were generated using the BC-Wizard [4].

Cartesian coordinates of the optimized ground-states (S_0) and excited-states (S_1)

Table S4: ***S_0:*** *5,13-diphenylpyrimido[4',5',6':9,1]pyrrolo[2',1',5':4,5,6]quinolizino[3,2-b]quinoline (**5a**)*

E = -1411.23 Hartree

Symbol	X	Y	Z
C	-0.059922	-3.234937	-0.035355
N	-0.965593	-2.247304	-0.037085
C	-0.505065	-0.983914	-0.025585
C	0.888569	-0.787045	-0.018922
C	1.772528	-1.886134	-0.023464
N	1.269907	-3.143559	-0.027696
H	-0.467822	-4.244025	-0.042406
N	1.417293	0.473829	-0.018065
C	0.669648	1.627403	-0.060577
C	2.780482	0.761862	-0.022667
C	1.576269	2.692654	-0.099220
C	2.876209	2.162599	-0.080393
H	1.300187	3.735523	-0.140507
H	3.797665	2.723571	-0.113521
C	3.181031	-1.630382	-0.011412
H	3.845739	-2.485829	0.021322
C	3.690027	-0.346206	-0.003881
C	-1.388420	0.196649	-0.030008
C	-0.768070	1.511729	-0.063018
C	-2.789834	0.141084	-0.010993
C	-3.523784	1.364074	-0.041444
C	-2.797733	2.601269	-0.087235
N	-1.447639	2.653203	-0.092912

C	-3.576425	-1.131983	0.054926
C	-3.916436	-1.685164	1.296317
C	-4.058222	-1.732003	-1.115653
C	-4.700439	-2.837254	1.363778
H	-3.559857	-1.216158	2.208595
C	-4.841520	-2.884588	-1.046211
H	-3.811449	-1.299977	-2.080969
C	-5.162008	-3.442781	0.193084
H	-4.952823	-3.259603	2.332064
H	-5.203506	-3.344253	-1.961260
H	-5.772692	-4.339147	0.246422
C	-4.948960	1.416136	-0.025885
C	-5.611145	2.620554	-0.060074
H	-6.696348	2.641213	-0.048660
C	-4.887372	3.840450	-0.109603
H	-5.426081	4.782810	-0.136243
C	-3.513933	3.831456	-0.122131
H	-2.935543	4.748704	-0.157267
H	-5.509027	0.489758	0.012072
C	5.155752	-0.110746	0.019066
C	6.006638	-0.877918	-0.794691
C	5.724648	0.854861	0.867324
C	7.386661	-0.683293	-0.762975
H	5.579530	-1.614087	-1.468512
C	7.105771	1.044667	0.900233
H	5.085210	1.438572	1.521040
C	7.940990	0.278685	0.084316
H	8.028191	-1.279240	-1.405076
H	7.529401	1.787956	1.568876
H	9.015853	0.430338	0.108454

*Table S5: **S_0:** N,N-dimethyl-5,13-diphenylpyrimido[4',5',6':9,1]pyrrolo[2',1',5':4,5,6]quinolizino[3,2-b]quinolin-11-amine (**5k**)*

E = -1545.21 Hartree

Symbol	X	Y	Z
C	-1.036960	3.385873	0.039147
N	-0.028573	2.504594	0.031724

C	-0.346929	1.196629	0.017231
C	-1.710647	0.848344	0.012565
C	-2.710428	1.844030	0.012884
N	-2.349242	3.149348	0.029729
H	-0.742452	4.433803	0.053330
N	-2.098696	-0.463204	-0.002046
C	-1.228578	-1.526126	-0.044022
C	-3.424451	-0.899013	-0.020900
C	-2.012700	-2.684282	-0.097086
C	-3.364003	-2.300541	-0.087989
H	-1.623723	-3.690416	-0.141828
H	-4.217298	-2.959933	-0.133974
C	-4.082296	1.435750	0.007235
H	-4.837114	2.212814	0.041442
C	-4.447772	0.102804	-0.002538
C	0.662413	0.122274	0.000811
C	0.187714	-1.250371	-0.035709
C	2.049375	0.335104	0.011904
C	2.921698	-0.797882	-0.011646
C	2.325142	-2.102758	-0.052257
N	0.993760	-2.308188	-0.063606
C	2.688076	1.689355	0.047790
C	3.013784	2.288943	1.271579
C	3.055593	2.330427	-1.142792
C	3.665775	3.522260	1.303447
H	2.746922	1.791460	2.199424
C	3.706864	3.564077	-1.110112
H	2.821379	1.865026	-2.095767
C	4.010660	4.165909	0.112974
H	3.905530	3.979237	2.259280
H	3.978571	4.053661	-2.040913
H	4.517880	5.125872	0.138338
C	4.334684	-0.681647	0.000782
C	5.166906	-1.804155	-0.020503
C	4.546420	-3.101064	-0.069503
H	5.161002	-3.992136	-0.095965
C	3.185387	-3.236735	-0.082887

H	2.724573	-4.218578	-0.118240
H	4.760898	0.309893	0.027187
C	-5.879566	-0.291025	0.002357
C	-6.800409	0.381646	-0.818719
C	-6.349277	-1.317162	0.840131
C	-8.150977	0.036152	-0.804824
H	-6.449226	1.164097	-1.484222
C	-7.701372	-1.658171	0.855384
H	-5.657258	-1.830164	1.499566
C	-8.606336	-0.985170	0.031969
H	-8.846430	0.561257	-1.452662
H	-8.048216	-2.446912	1.516224
H	-9.658173	-1.254410	0.042166
N	6.539437	-1.694202	0.008388
C	7.151242	-0.374478	-0.005354
H	8.234730	-0.479586	0.040066
H	6.897575	0.189176	-0.914168
H	6.832261	0.220700	0.859114
C	7.385617	-2.873122	-0.122639
H	7.236369	-3.391016	-1.079697
H	8.429384	-2.565964	-0.066528
H	7.208113	-3.591559	0.686245

*Table S6: **S_0:** 5,13-diphenyl-11-(trifluoromethyl)pyrimido[4',5',6':9,1]pyrrolo[2',1',5':4,5,6]quinolizino[3,2-b]quinoline (**5l**)*

E = -1748.27 Hartree

Symbol	X	Y	Z
C	-1.316856	3.420121	0.019843
N	-0.289011	2.559949	0.013280
C	-0.580028	1.247730	0.005826
C	-1.934769	0.868203	0.004661
C	-2.955901	1.841288	0.004123
N	-2.622959	3.153426	0.015494
H	-1.045212	4.473867	0.028333
N	-2.291914	-0.451188	-0.006504
C	-1.397712	-1.496488	-0.050181
C	-3.603650	-0.916491	-0.021423

C	-2.156113	-2.672105	-0.100331
C	-3.513683	-2.317853	-0.087566
H	-1.745202	-3.669435	-0.145412
H	-4.353014	-2.994879	-0.130641
C	-4.318296	1.401443	0.003541
H	-5.090798	2.160895	0.037166
C	-4.652625	0.061257	-0.001744
C	0.451440	0.193292	-0.008623
C	0.010107	-1.192451	-0.045786
C	1.832068	0.434777	0.004167
C	2.718626	-0.683077	-0.024843
C	2.164046	-2.004011	-0.067898
N	0.835528	-2.235377	-0.076382
C	2.446333	1.799605	0.050568
C	2.740144	2.400516	1.281412
C	2.816581	2.448077	-1.134701
C	3.369175	3.645138	1.324172
H	2.469023	1.896249	2.204141
C	3.444187	3.693388	-1.089048
H	2.605112	1.980640	-2.091810
C	3.719067	4.296988	0.139777
H	3.588005	4.103641	2.284030
H	3.721271	4.189533	-2.014502
H	4.209023	5.265403	0.174440
C	4.135363	-0.541010	-0.011600
C	4.949510	-1.647599	-0.042831
C	4.398646	-2.958569	-0.088523
H	5.059632	-3.818733	-0.113538
C	3.039702	-3.128820	-0.100391
H	2.591470	-4.115728	-0.133832
H	4.569342	0.449162	0.022759
C	-6.073733	-0.368124	0.008111
C	-7.011896	0.276602	-0.815678
C	-6.515646	-1.400293	0.853548
C	-8.353290	-0.102458	-0.796939
H	-6.681662	1.062884	-1.487344
C	-7.858875	-1.774181	0.873810

H	-5.810458	-1.891660	1.515572
C	-8.781462	-1.129126	0.047590
H	-9.062694	0.400744	-1.446917
H	-8.184984	-2.566693	1.540560
H	-9.826232	-1.424277	0.061570
C	6.444599	-1.506842	-0.026364
F	7.009477	-2.086111	-1.114427
F	6.849852	-0.220255	-0.002999
F	6.989063	-2.118395	1.054552

*Table S7: **S_0:** phenyl(8-phenylpyrimido[4,5,6-ij]pyrrolo[2,1,5-de]quinolizin-4-yl)methanone (**6a**)*

E = -1201.23 Hartree

Symbol	X	Y	Z
C	0.385146	3.444816	0.640676
N	-0.926172	3.186838	0.688766
C	-1.290390	1.922348	0.367277
C	-0.293231	0.989557	0.012396
C	1.066299	1.367941	-0.037175
N	1.395333	2.634407	0.302128
H	0.673181	4.458723	0.913639
N	-0.666118	-0.283220	-0.291237
C	-1.970149	-0.752218	-0.296078
C	0.226899	-1.277339	-0.670486
C	-1.892041	-2.101453	-0.712469
C	-0.552283	-2.422407	-0.935433
H	-2.737470	-2.760666	-0.838456
H	-0.161605	-3.376433	-1.259365
C	2.030284	0.355668	-0.416655
C	1.590908	-0.927109	-0.708112
C	-2.998792	0.171693	0.067194
C	-2.647350	1.474325	0.384192
H	-3.408226	2.185015	0.686128
C	-4.418663	-0.261002	0.102232
C	-5.420576	0.562198	-0.438906
C	-4.793310	-1.479223	0.694377
C	-6.759188	0.175716	-0.392854

H	-5.144027	1.497676	-0.915257
C	-6.133488	-1.861621	0.742435
H	-4.036582	-2.114139	1.143135
C	-7.120176	-1.037503	0.197395
H	-7.519382	0.820158	-0.823759
H	-6.406696	-2.801491	1.212487
H	-8.162852	-1.338175	0.232673
H	2.304679	-1.688744	-1.002533
C	3.474561	0.711584	-0.624386
8	3.772331	1.718452	-1.257912
C	4.538050	-0.193615	-0.084652
C	5.828085	-0.096553	-0.633550
C	4.310202	-1.084496	0.976464
C	6.862889	-0.888000	-0.146056
H	5.995666	0.604328	-1.444189
C	5.352640	-1.867606	1.473103
H	3.326144	-1.150638	1.427489
C	6.626573	-1.775441	0.909589
H	7.853943	-0.814811	-0.583350
H	5.170434	-2.546114	2.300703
H	7.435465	-2.390031	1.293236

*Table S8: **S_0:** pyrimido[4',5',6':9,1]pyrrolo[2',1',5':4,5,6]quinolizino[3,2-b]quinoline*

E = -949.34 Hartree

Symbol	X	Y	Z
C	0.821375	1.305621	-0.000047
C	1.952610	0.480296	-0.000080
C	3.242269	1.044415	-0.000139
N	1.806653	-0.879840	-0.000057
C	0.605821	-1.555064	-0.000001
C	2.876026	-1.768427	-0.000089
C	0.916013	-2.918237	0.000007
C	2.313185	-3.052699	-0.000054
H	0.181176	-3.706451	0.000048
H	2.872392	-3.974991	-0.000069
C	4.367839	0.153128	-0.000172

H	5.358267	0.588931	-0.000218
C	4.178399	-1.204271	-0.000148
C	-0.603735	-0.771955	0.000035
C	-0.494879	0.670764	0.000012
H	5.026928	-1.877584	-0.000174
C	-2.906125	0.757547	0.000103
C	-2.904558	-0.679447	0.000121
C	-5.329057	-0.657327	0.000211
H	-6.274859	-1.187468	0.000253
C	-5.329351	0.762223	0.000192
H	-6.272487	1.296108	0.000221
N	-1.767375	-1.409215	0.000088
C	-1.662187	1.409857	0.000048
C	-4.145174	1.453129	0.000140
H	-4.134623	2.537898	0.000126
H	-1.599674	2.492304	0.000031
C	-4.152073	-1.361362	0.000177
H	-4.131332	-2.444175	0.000190
C	2.239017	3.090116	-0.000126
H	2.349640	4.170391	-0.000144
N	0.977294	2.637239	-0.000070
N	3.370867	2.388273	-0.000162

*Table S9: **S_1:** 5,13-diphenylpyrimido[4',5',6':9,1]pyrrolo[2',1',5':4,5,6]quinolizino[3,2-b]quinoline (**5a**)*

E = -1411.16 Hartree

Symbol	X	Y	Z
C	-0.049461	-3.262482	-0.123383
N	-0.959016	-2.278473	-0.104108
C	-0.509464	-0.994037	-0.058933
C	0.897089	-0.817512	-0.045358
C	1.779603	-1.906019	-0.071062
N	1.283732	-3.182302	-0.106433
H	-0.458711	-4.270976	-0.158399
N	1.432059	0.436697	-0.020204
C	0.664836	1.585533	-0.053857
C	2.781570	0.735087	-0.016627

C	1.573468	2.680286	-0.076968
C	2.858875	2.173546	-0.059465
H	1.279054	3.718715	-0.110879
H	3.777778	2.740793	-0.087881
C	3.178933	-1.639309	-0.048887
H	3.851353	-2.491345	-0.036615
C	3.705717	-0.337945	-0.011086
C	-1.387439	0.166125	-0.039280
C	-0.767360	1.477715	-0.066290
C	-2.802470	0.131642	-0.014681
C	-3.523320	1.380147	-0.047850
C	-2.785313	2.614307	-0.097464
N	-1.419810	2.638743	-0.098313
C	-3.607189	-1.125478	0.074662
C	-3.879399	-1.708957	1.321663
C	-4.173539	-1.699160	-1.073981
C	-4.669535	-2.858002	1.415611
H	-3.459721	-1.265252	2.221422
C	-4.963041	-2.848987	-0.983523
H	-3.982842	-1.248769	-2.045253
C	-5.210794	-3.435339	0.262144
H	-4.862535	-3.301246	2.389680
H	-5.384531	-3.285787	-1.885770
H	-5.824472	-4.329854	0.333826
C	-4.935465	1.462750	-0.025341
C	-5.592546	2.694158	-0.064185
H	-6.679069	2.718789	-0.047040
C	-4.864299	3.893428	-0.123128
H	-5.381599	4.848281	-0.153848
C	-3.472817	3.848756	-0.137100
H	-2.879802	4.758404	-0.175771
H	-5.519998	0.550555	0.021823
C	5.166994	-0.104247	0.027893
C	6.027956	-0.864511	-0.786465
C	5.728428	0.857037	0.889799
C	7.408194	-0.662648	-0.745414
H	5.612552	-1.599113	-1.471049

C	7.109726	1.054526	0.930939
H	5.086578	1.428202	1.554582
C	7.954635	0.298078	0.112303
H	8.056197	-1.252052	-1.388558
H	7.526002	1.793518	1.610344
H	9.029570	0.453561	0.144676

Table S10: ***S_1:*** *N,N-dimethyl-5,13-diphenylpyrimido[4',5',6':9,1]pyrrolo[2',1',5':4,5,6]quinolizino[3,2-b]quinolin-11-amine (**5k**)*

E = -1545.15 Hartree

Symbol	X	Y	Z
C	-1.044098	3.407220	-0.094817
N	-0.031823	2.536333	-0.081978
C	-0.346353	1.205015	-0.046364
C	-1.718674	0.869049	-0.034195
C	-2.721383	1.855321	-0.052674
N	-2.362286	3.178727	-0.079119
H	-0.752672	4.457016	-0.122747
N	-2.107089	-0.440029	-0.014355
C	-1.214719	-1.500264	-0.052515
C	-3.418669	-0.887870	-0.010379
C	-1.999087	-2.675865	-0.078361
C	-3.342896	-2.308138	-0.055745
H	-1.601289	-3.680146	-0.113400
H	-4.191485	-2.976437	-0.080061
C	-4.082553	1.436715	-0.032896
H	-4.843867	2.210717	-0.014326
C	-4.457421	0.093386	-0.003451
C	0.656948	0.148900	-0.034527
C	0.187337	-1.233436	-0.064619
C	2.053583	0.350526	-0.015615
C	2.915675	-0.805606	-0.046390
C	2.328779	-2.112454	-0.099911
N	0.989314	-2.306718	-0.103552
C	2.706581	1.693964	0.059740
C	2.879161	2.336253	1.295638
C	3.231962	2.299776	-1.092214

C	3.534697	3.567436	1.374814
H	2.485623	1.873290	2.197304
C	3.883620	3.534432	-1.016423
H	3.113871	1.808891	-2.055385
C	4.035857	4.174189	0.217855
H	3.652612	4.053647	2.340322
H	4.272071	3.994768	-1.921774
H	4.542661	5.134022	0.278517
C	4.318026	-0.706053	-0.009154
C	5.161796	-1.850029	-0.042609
C	4.556419	-3.143687	-0.114535
H	5.161167	-4.040626	-0.146085
C	3.182337	-3.249604	-0.138683
H	2.711164	-4.227587	-0.185601
H	4.759687	0.278403	0.050860
C	-5.884848	-0.302776	0.022920
C	-6.824777	0.367283	-0.784505
C	-6.346286	-1.330903	0.867805
C	-8.176658	0.020907	-0.749199
H	-6.489122	1.150822	-1.458564
C	-7.698906	-1.675806	0.902840
H	-5.648098	-1.842584	1.523866
C	-8.620947	-1.003564	0.093729
H	-8.882092	0.547125	-1.387299
H	-8.033432	-2.465879	1.570439
H	-9.673157	-1.274230	0.120262
N	6.521384	-1.705970	-0.003053
C	7.141457	-0.382316	0.065409
H	8.223855	-0.499757	0.076319
H	6.867387	0.226118	-0.803828
H	6.840435	0.147084	0.976612
C	7.386161	-2.883806	-0.026629
H	7.240444	-3.457629	-0.949618
H	8.425853	-2.565058	0.023675
H	7.179158	-3.537546	0.828854

1H-, 13C- and 19F-NMR Spectra

1-(4,6-dichloropyrimidin-5-yl)-1*H*-pyrrole-2-carbaldehyde (3)

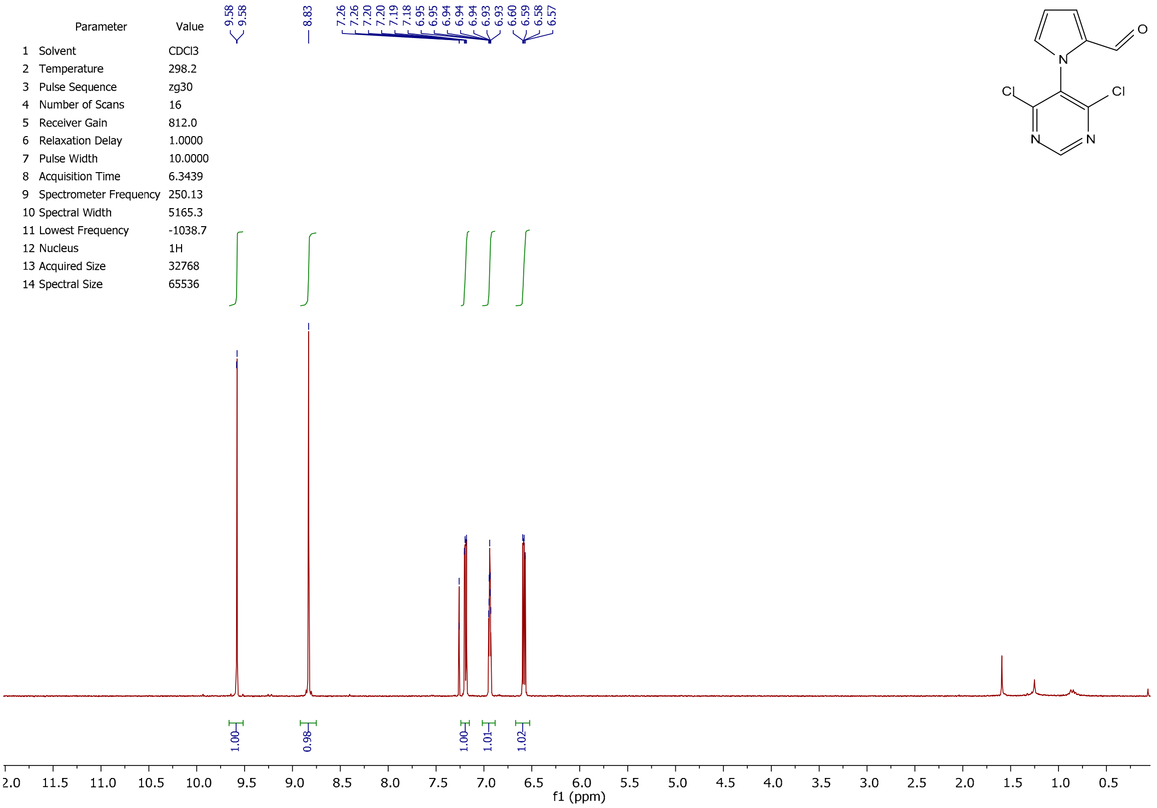

	Parameter	Value
1	Solvent	CDCl3
2	Temperature	298.2
3	Pulse Sequence	zg30
4	Number of Scans	16
5	Receiver Gain	812.0
6	Relaxation Delay	1.0000
7	Pulse Width	10.0000
8	Acquisition Time	6.3439
9	Spectrometer Frequency	250.13
10	Spectral Width	5165.3
11	Lowest Frequency	-1038.7
12	Nucleus	1H
13	Acquired Size	32768
14	Spectral Size	65536

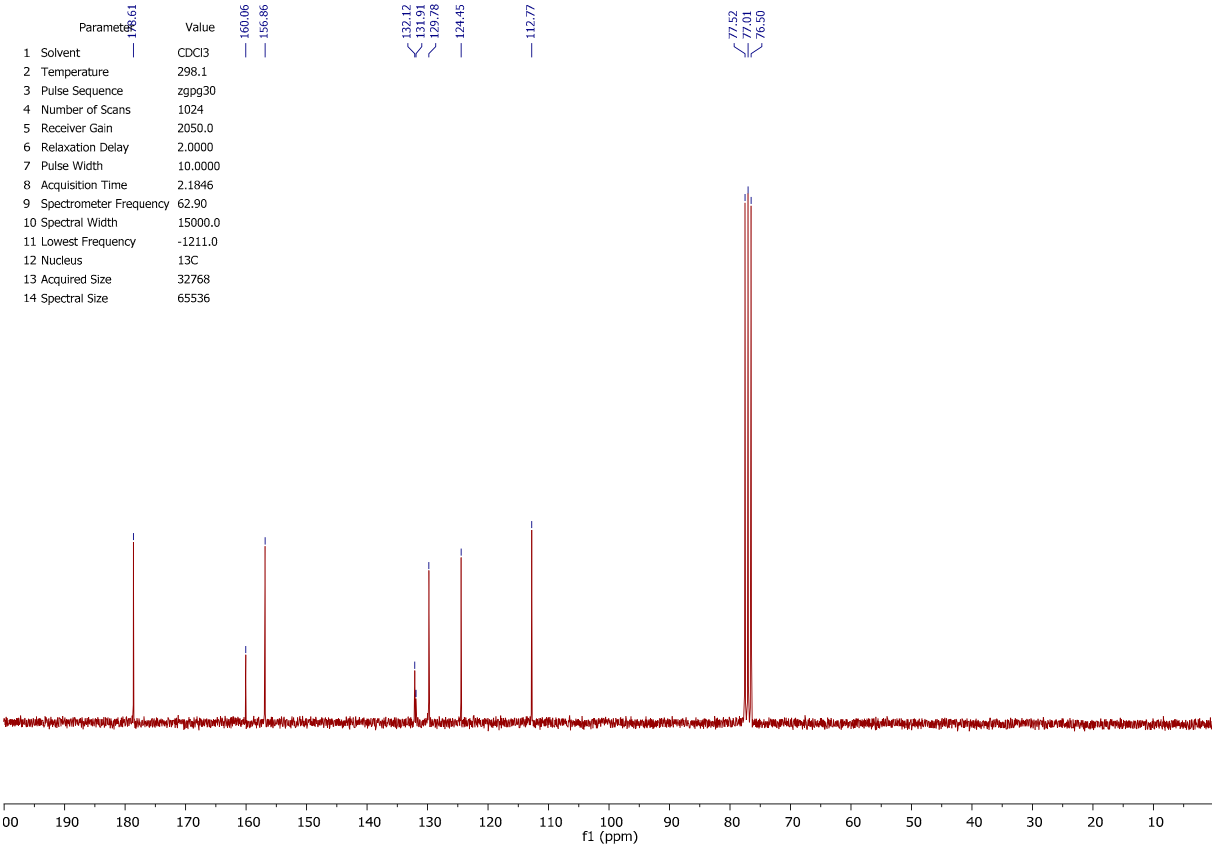

	Parameter	Value
1	Solvent	CDCl3
2	Temperature	298.1
3	Pulse Sequence	zgpg30
4	Number of Scans	1024
5	Receiver Gain	2050.0
6	Relaxation Delay	2.0000
7	Pulse Width	10.0000
8	Acquisition Time	2.1846
9	Spectrometer Frequency	62.90
10	Spectral Width	15000.0
11	Lowest Frequency	-1211.0
12	Nucleus	13C
13	Acquired Size	32768
14	Spectral Size	65536

1-(4,6-bis(phenylethynyl)pyrimidin-5-yl)-1*H*-pyrrole-2-carbaldehyde (4a)

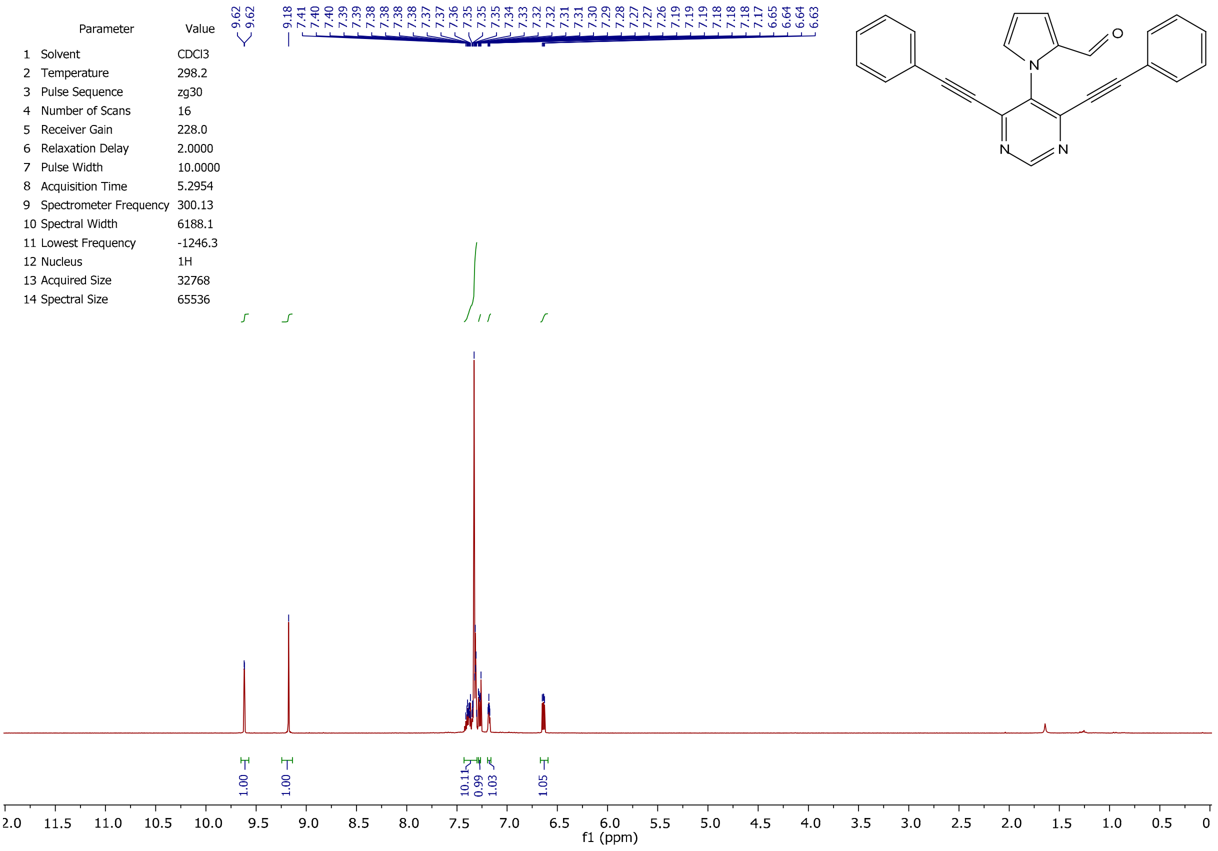

	Parameter	Value
1	Solvent	CDCl3
2	Temperature	298.2
3	Pulse Sequence	zg30
4	Number of Scans	16
5	Receiver Gain	228.0
6	Relaxation Delay	2.0000
7	Pulse Width	10.0000
8	Acquisition Time	5.2954
9	Spectrometer Frequency	300.13
10	Spectral Width	6188.1
11	Lowest Frequency	-1246.3
12	Nucleus	1H
13	Acquired Size	32768
14	Spectral Size	65536

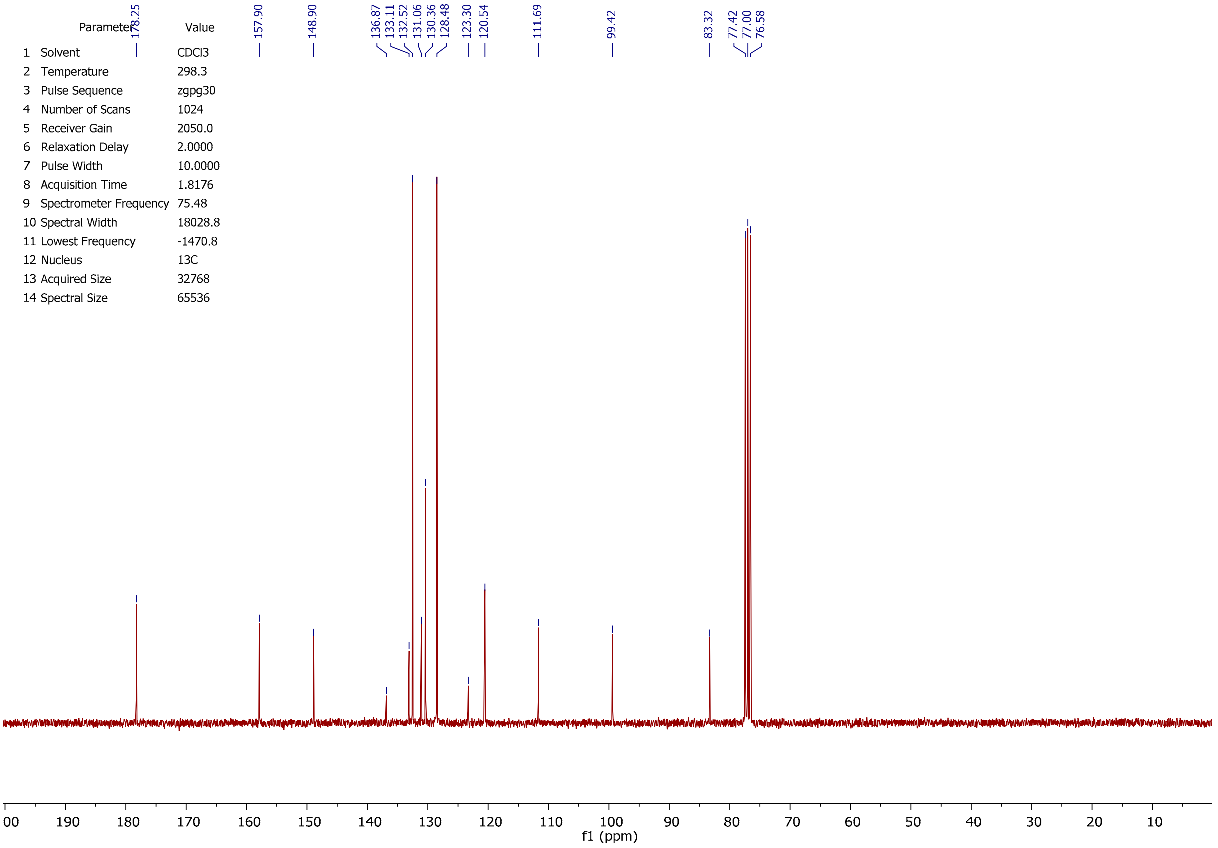

	Parameter	Value
1	Solvent	CDCl3
2	Temperature	298.3
3	Pulse Sequence	zgpg30
4	Number of Scans	1024
5	Receiver Gain	2050.0
6	Relaxation Delay	2.0000
7	Pulse Width	10.0000
8	Acquisition Time	1.8176
9	Spectrometer Frequency	75.48
10	Spectral Width	18028.8
11	Lowest Frequency	-1470.8
12	Nucleus	13C
13	Acquired Size	32768
14	Spectral Size	65536

1-(4,6-bis(*p*-tolylethynyl)pyrimidin-5-yl)-1*H*-pyrrole-2-carbaldehyde (4b)

Parameter	Value
1 Solvent	CDCl3
2 Temperature	298.2
3 Pulse Sequence	zg30
4 Number of Scans	16
5 Receiver Gain	362.0
6 Relaxation Delay	1.0000
7 Pulse Width	10.0000
8 Acquisition Time	6.3439
9 Spectrometer Frequency	250.13
10 Spectral Width	5165.3
11 Lowest Frequency	-1038.2
12 Nucleus	1H
13 Acquired Size	32768
14 Spectral Size	65536

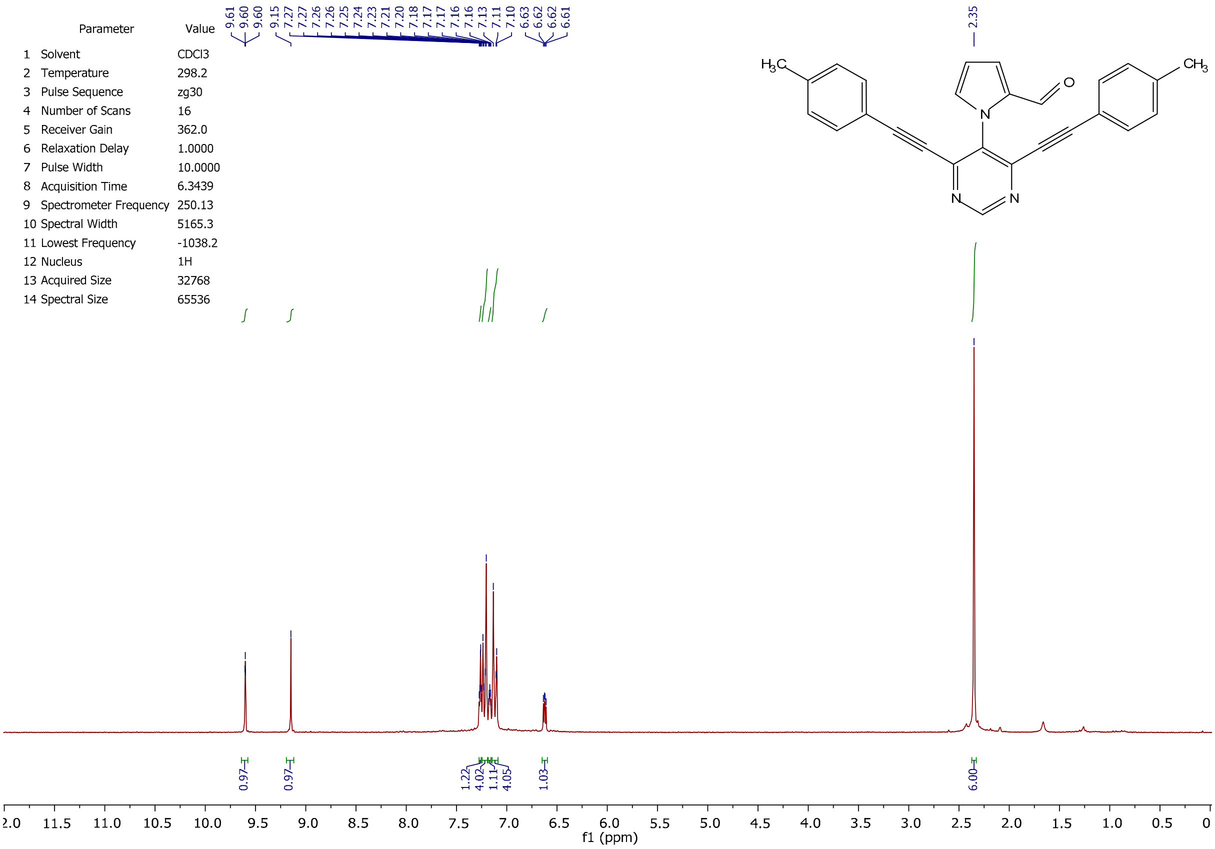

Parameter	Value
1 Solvent	CDCl3
2 Temperature	298.2
3 Pulse Sequence	zgpg30
4 Number of Scans	1024
5 Receiver Gain	2050.0
6 Relaxation Delay	2.0000
7 Pulse Width	10.0000
8 Acquisition Time	2.1846
9 Spectrometer Frequency	62.90
10 Spectral Width	15000.0
11 Lowest Frequency	-1211.0
12 Nucleus	13C
13 Acquired Size	32768
14 Spectral Size	65536

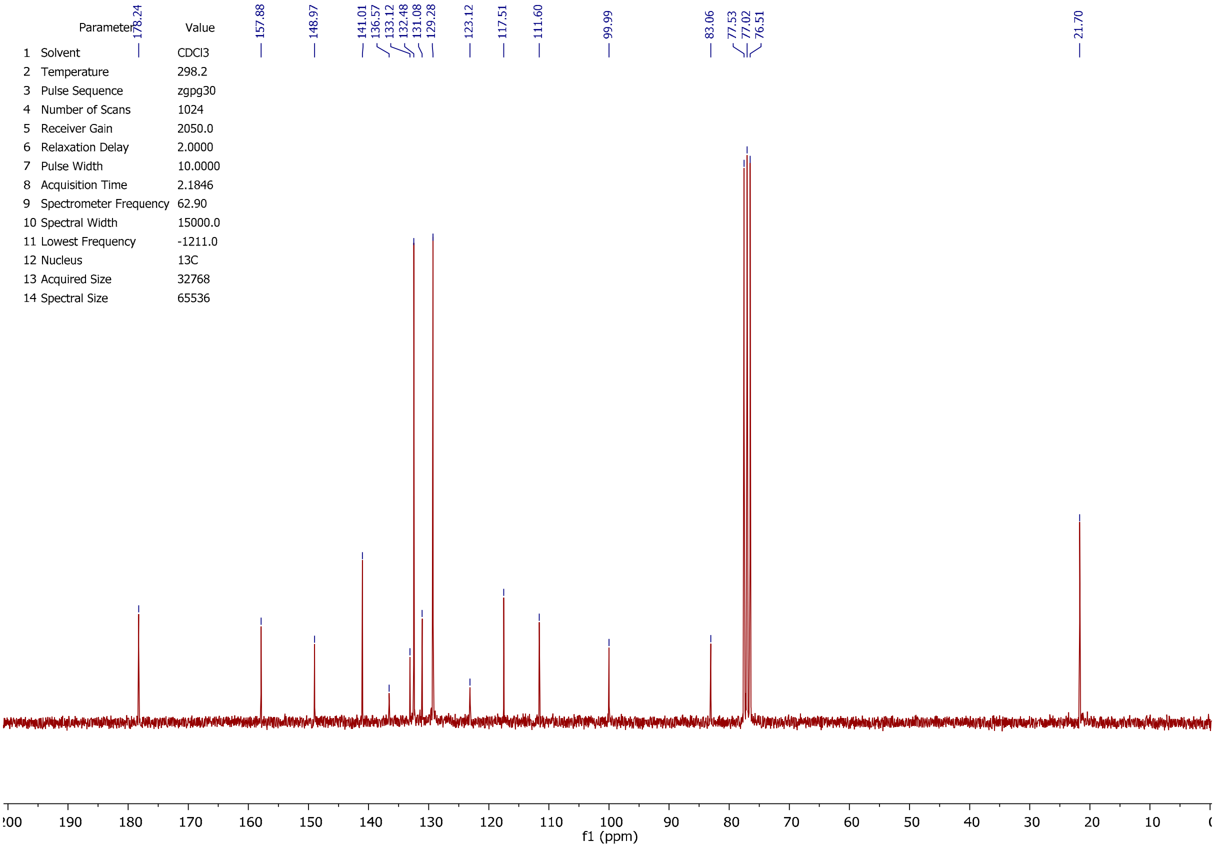

1-(4,6-bis((4-fluorophenyl)ethynyl)pyrimidin-5-yl)-1*H*-pyrrole-2-carbaldehyde (4c)

Parameter	Value
1 Solvent	CDCl3
2 Temperature	298.2
3 Pulse Sequence	zg30
4 Number of Scans	16
5 Receiver Gain	181.0
6 Relaxation Delay	2.0000
7 Pulse Width	10.0000
8 Acquisition Time	5.2954
9 Spectrometer Frequency	300.13
10 Spectral Width	6188.1
11 Lowest Frequency	-1246.4
12 Nucleus	1H
13 Acquired Size	32768
14 Spectral Size	65536

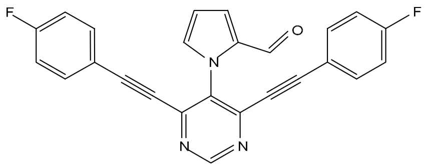

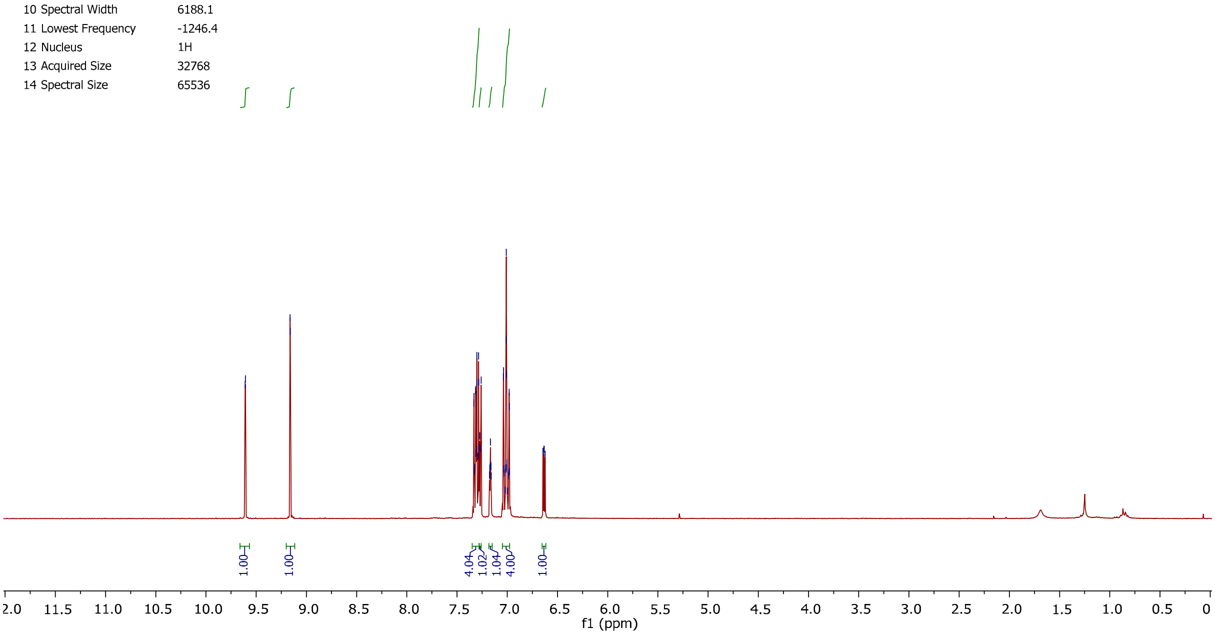

Parameter	Value
1 Solvent	CDCl3
2 Temperature	298.9
3 Pulse Sequence	zgpg30
4 Number of Scans	1024
5 Receiver Gain	2050.0
6 Relaxation Delay	2.0000
7 Pulse Width	10.0000
8 Acquisition Time	1.8176
9 Spectrometer Frequency	75.48
10 Spectral Width	18028.8
11 Lowest Frequency	-1470.9
12 Nucleus	13C
13 Acquired Size	32768
14 Spectral Size	65536

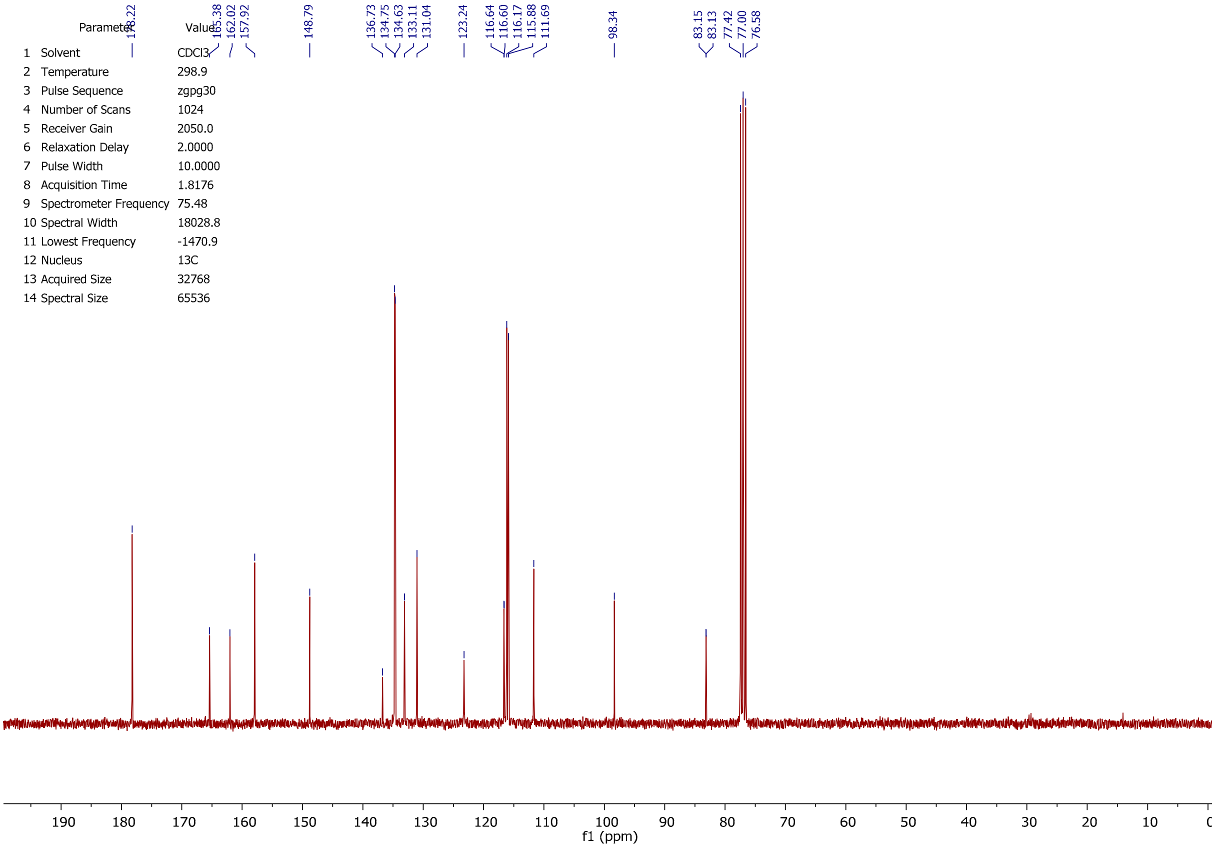

	Parameter	Value
1	Solvent	CDCl3
2	Temperature	298.2
3	Pulse Sequence	zgfhigqn
4	Number of Scans	64
5	Receiver Gain	2050.0
6	Relaxation Delay	1.0000
7	Pulse Width	10.0000
8	Acquisition Time	0.9787
9	Spectrometer Frequency	282.38
10	Spectral Width	66964.3
11	Lowest Frequency	-61722.4
12	Nucleus	19F
13	Acquired Size	65536
14	Spectral Size	131072

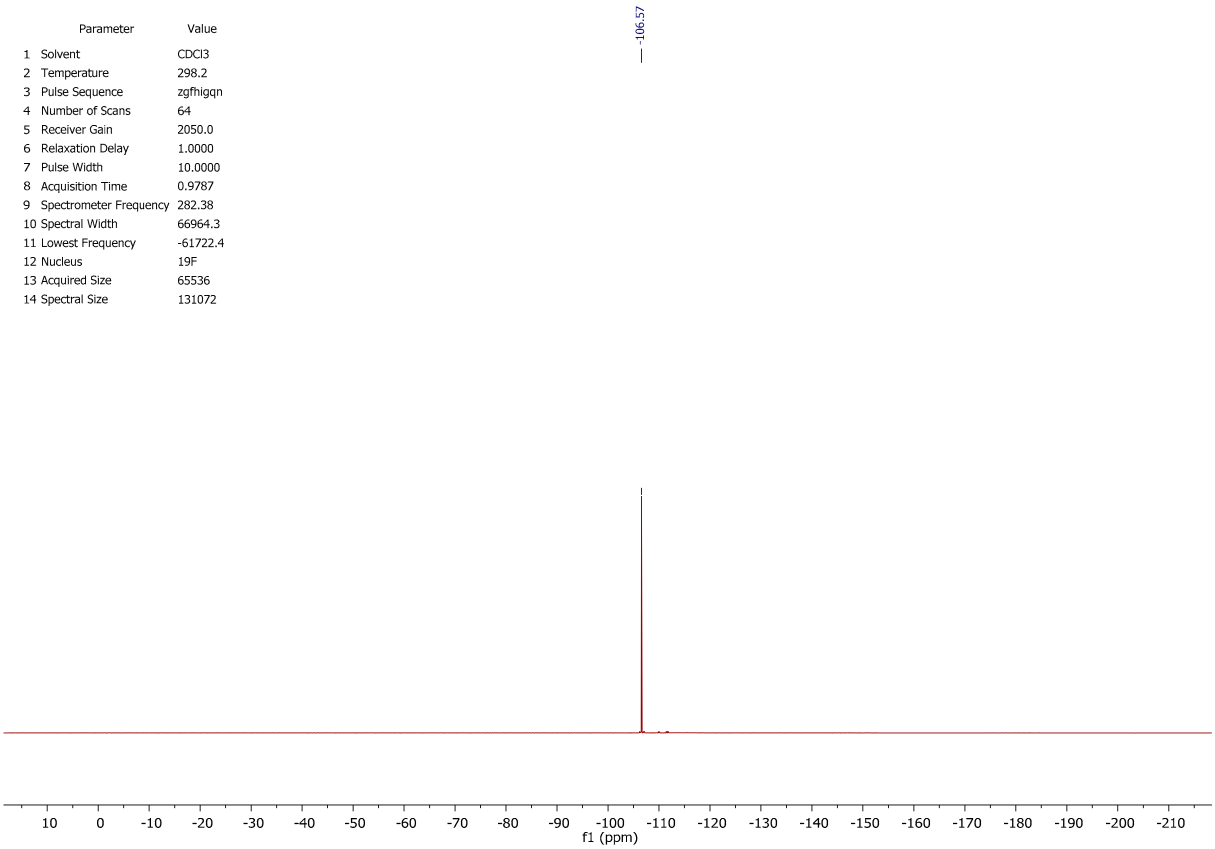

1-(4,6-Bis((4-(dimethylamino)phenyl)ethinyl)pyrimidin-5-yl)-1*H*-pyrrol-2-carbaldehyd (4d)

	Parameter	Value
1	Solvent	CDCl3
2	Temperature	298.2
3	Pulse Sequence	zg30
4	Number of Scans	16
5	Receiver Gain	181.0
6	Relaxation Delay	2.0000
7	Pulse Width	10.0000
8	Acquisition Time	5.2954
9	Spectrometer Frequency	300.13
10	Spectral Width	6188.1
11	Lowest Frequency	-1246.6
12	Nucleus	1H
13	Acquired Size	32768
14	Spectral Size	65536

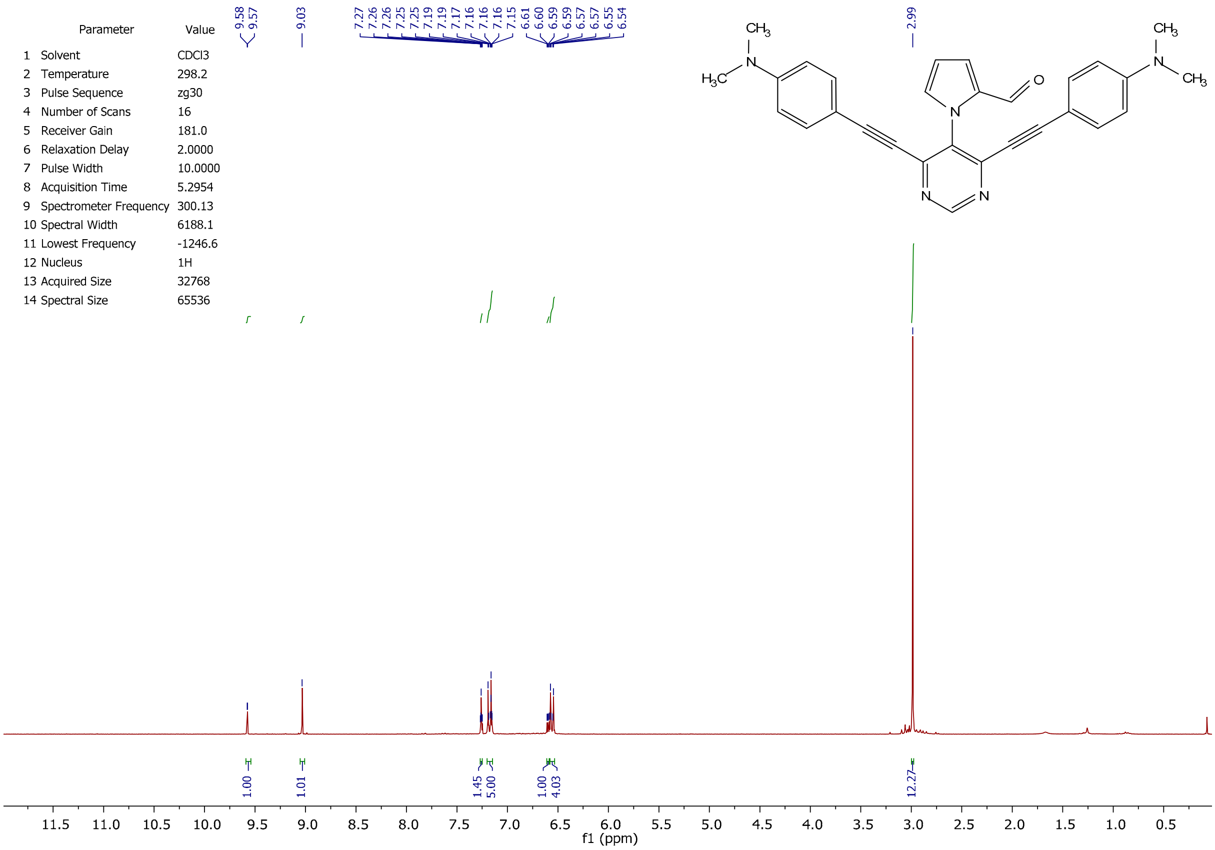

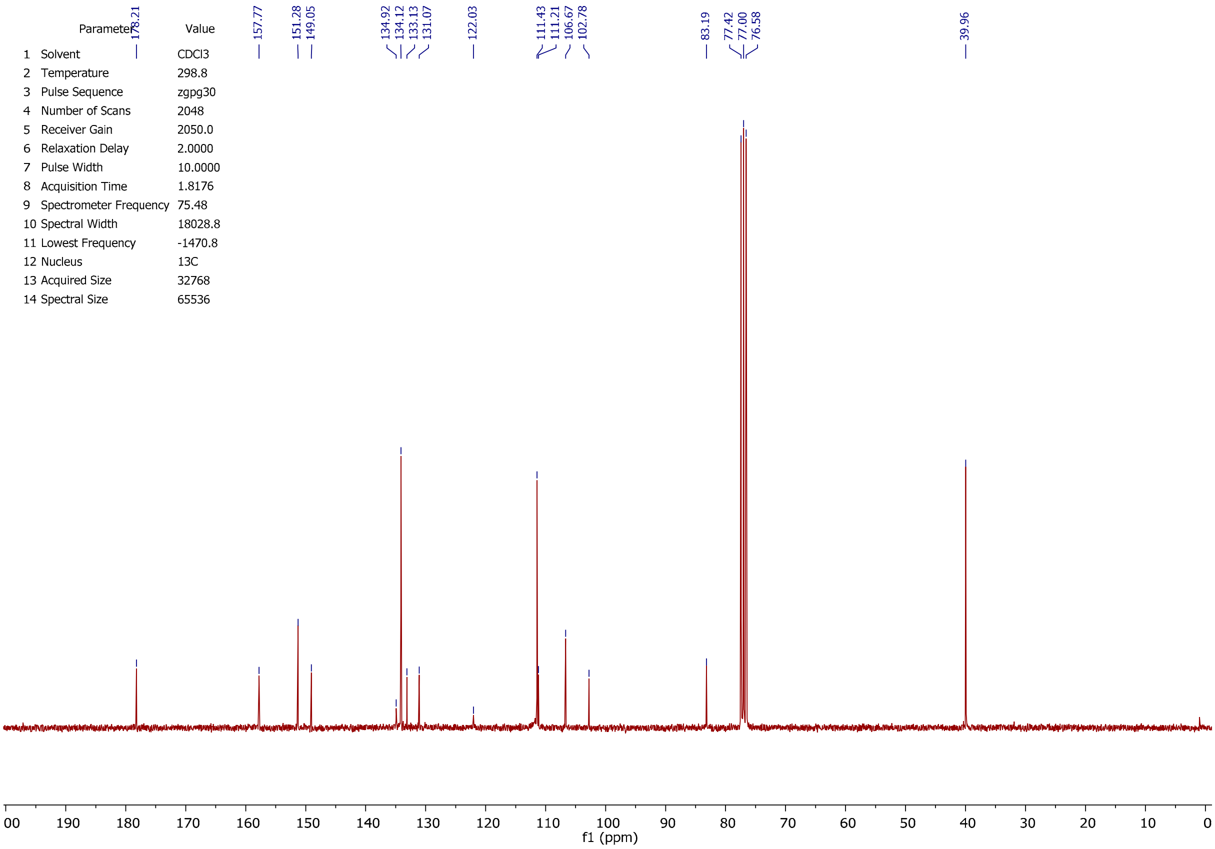

	Parameter	Value
1	Solvent	CDCl3
2	Temperature	298.8
3	Pulse Sequence	zgpg30
4	Number of Scans	2048
5	Receiver Gain	2050.0
6	Relaxation Delay	2.0000
7	Pulse Width	10.0000
8	Acquisition Time	1.8176
9	Spectrometer Frequency	75.48
10	Spectral Width	18028.8
11	Lowest Frequency	-1470.8
12	Nucleus	13C
13	Acquired Size	32768
14	Spectral Size	65536

1-(4,6-bis((4-methoxyphenyl)ethynyl)pyrimidin-5-yl)-1*H*-pyrrole-2-carbaldehyde (4e)

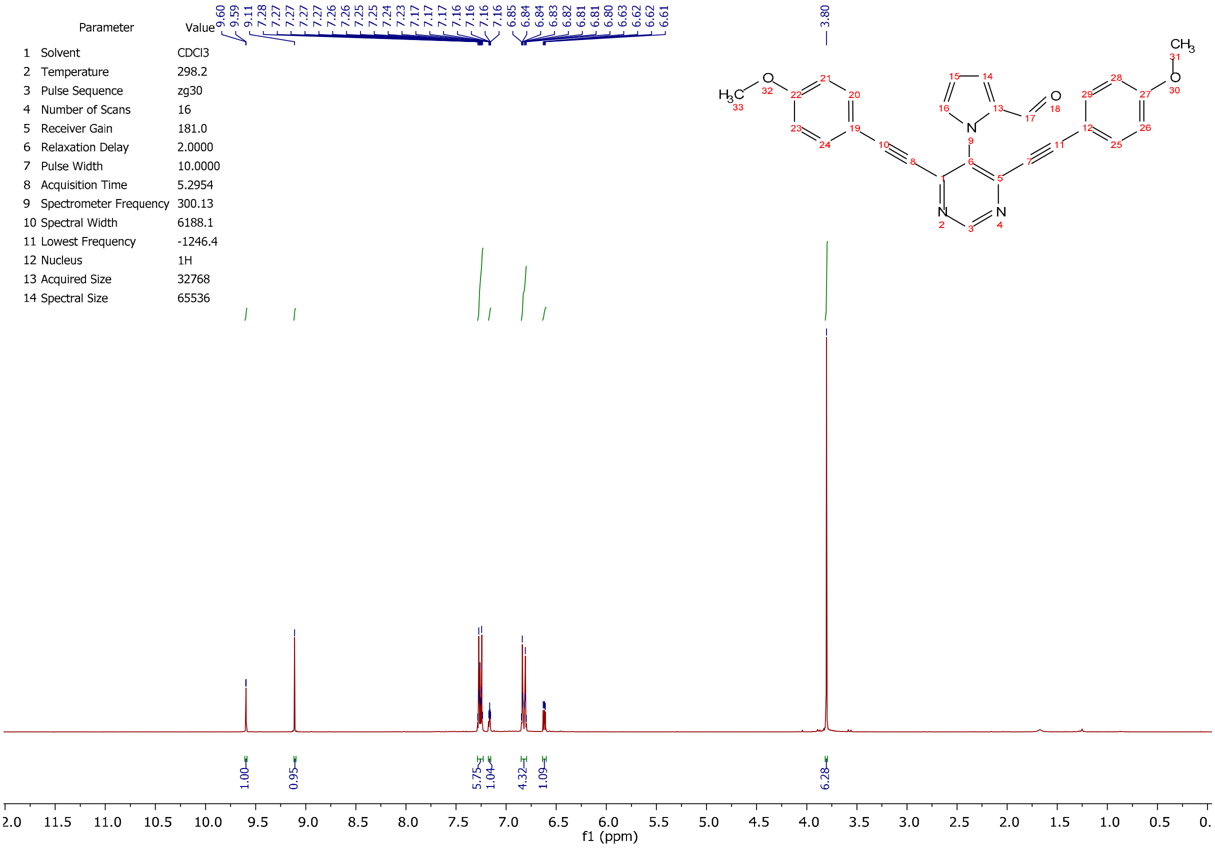

	Parameter	Value
1	Solvent	CDCl3
2	Temperature	298.2
3	Pulse Sequence	zg30
4	Number of Scans	16
5	Receiver Gain	181.0
6	Relaxation Delay	2.0000
7	Pulse Width	10.0000
8	Acquisition Time	5.2954
9	Spectrometer Frequency	300.13
10	Spectral Width	6188.1
11	Lowest Frequency	-1246.4
12	Nucleus	1H
13	Acquired Size	32768
14	Spectral Size	65536

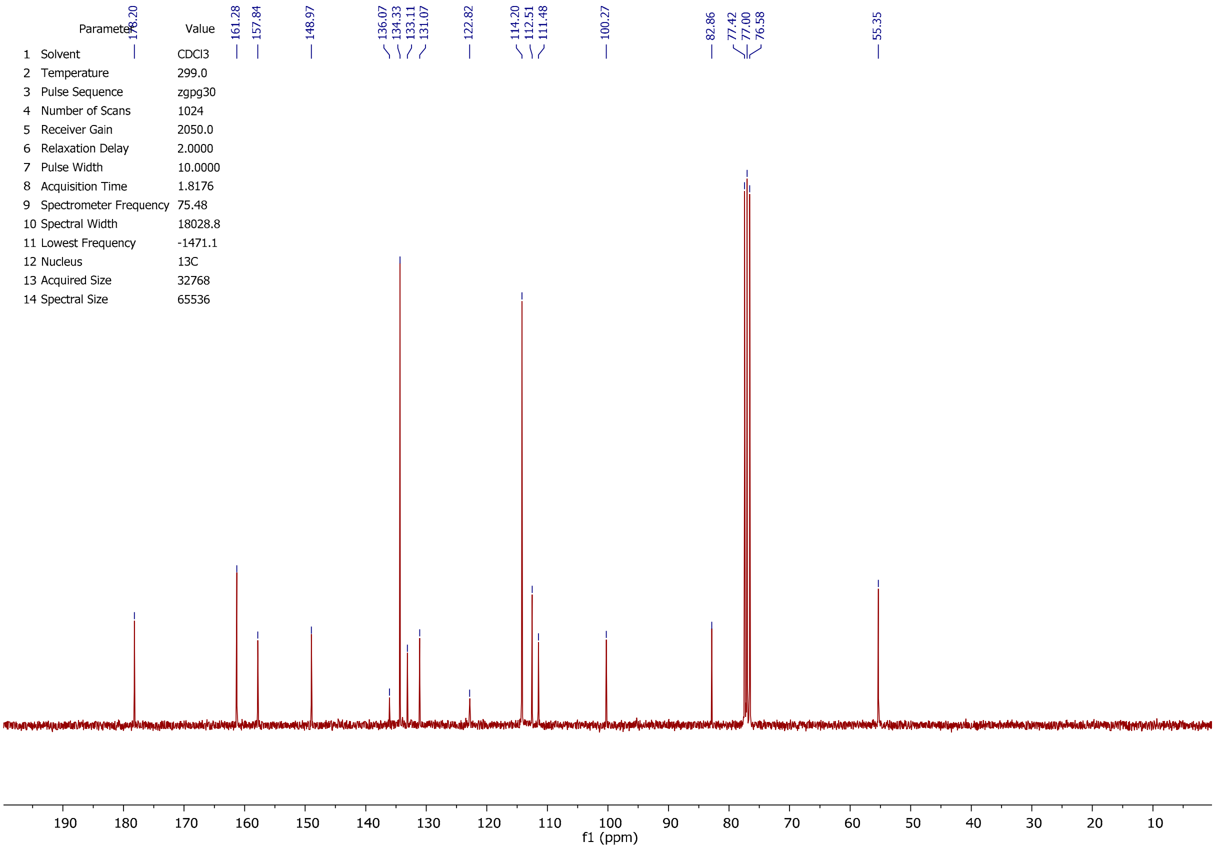

	Parameter	Value
1	Solvent	CDCl3
2	Temperature	299.0
3	Pulse Sequence	zgpg30
4	Number of Scans	1024
5	Receiver Gain	2050.0
6	Relaxation Delay	2.0000
7	Pulse Width	10.0000
8	Acquisition Time	1.8176
9	Spectrometer Frequency	75.48
10	Spectral Width	18028.8
11	Lowest Frequency	-1471.1
12	Nucleus	13C
13	Acquired Size	32768
14	Spectral Size	65536

1-(4,6-bis((4-(trifluoromethyl)phenyl)ethynyl)pyrimidin-5-yl)-1*H*-pyrrole-2-carbaldehyde (4f)

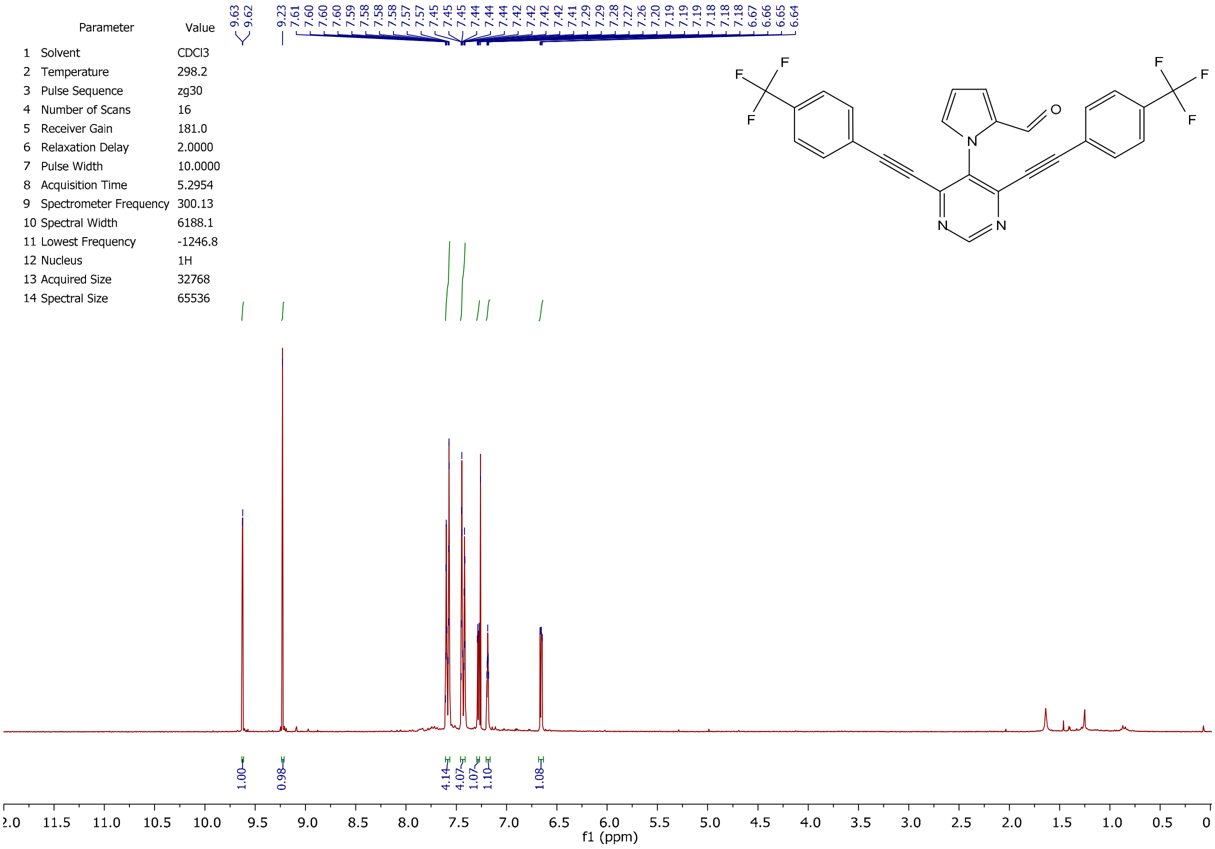

	Parameter	Value
1	Solvent	CDCl3
2	Temperature	298.2
3	Pulse Sequence	zg30
4	Number of Scans	16
5	Receiver Gain	181.0
6	Relaxation Delay	2.0000
7	Pulse Width	10.0000
8	Acquisition Time	5.2954
9	Spectrometer Frequency	300.13
10	Spectral Width	6188.1
11	Lowest Frequency	-1246.8
12	Nucleus	1H
13	Acquired Size	32768
14	Spectral Size	65536

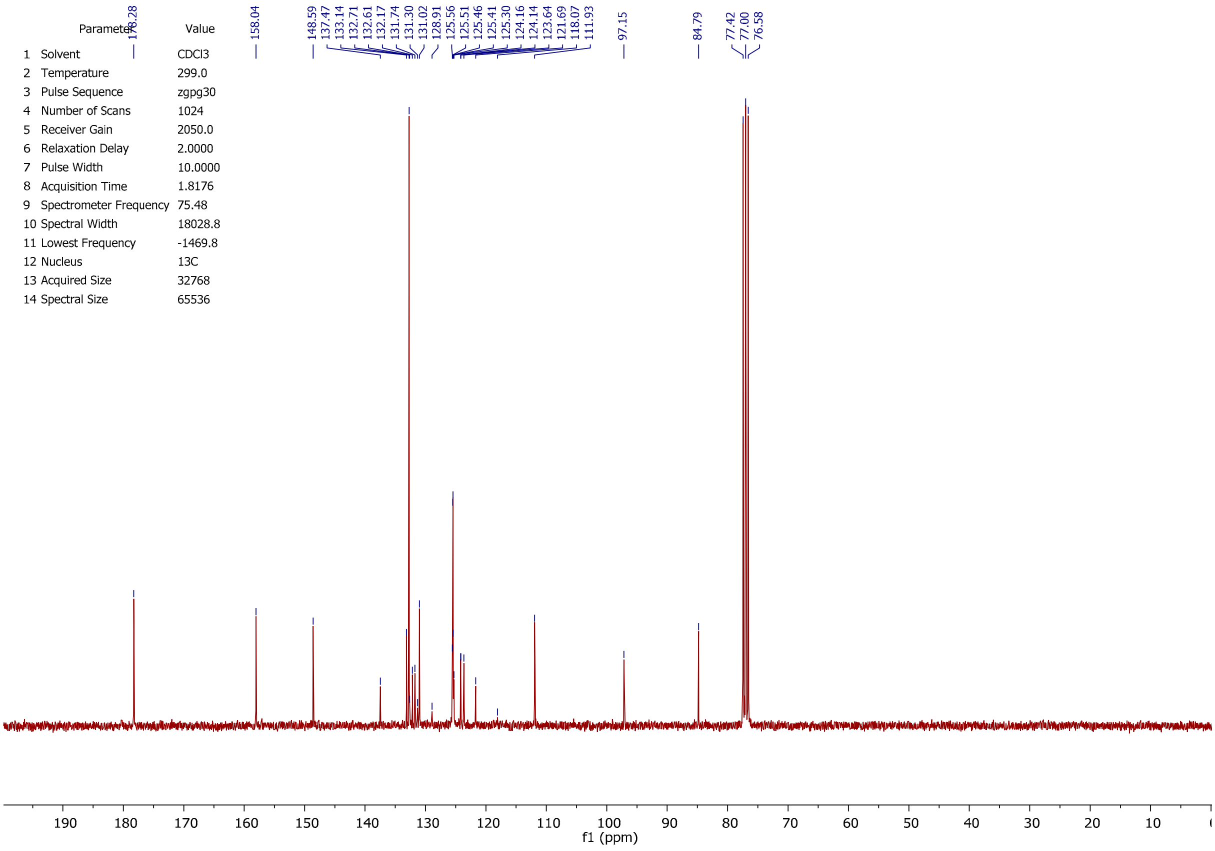

	Parameter	Value
1	Solvent	CDCl3
2	Temperature	299.0
3	Pulse Sequence	zgpg30
4	Number of Scans	1024
5	Receiver Gain	2050.0
6	Relaxation Delay	2.0000
7	Pulse Width	10.0000
8	Acquisition Time	1.8176
9	Spectrometer Frequency	75.48
10	Spectral Width	18028.8
11	Lowest Frequency	-1469.8
12	Nucleus	13C
13	Acquired Size	32768
14	Spectral Size	65536

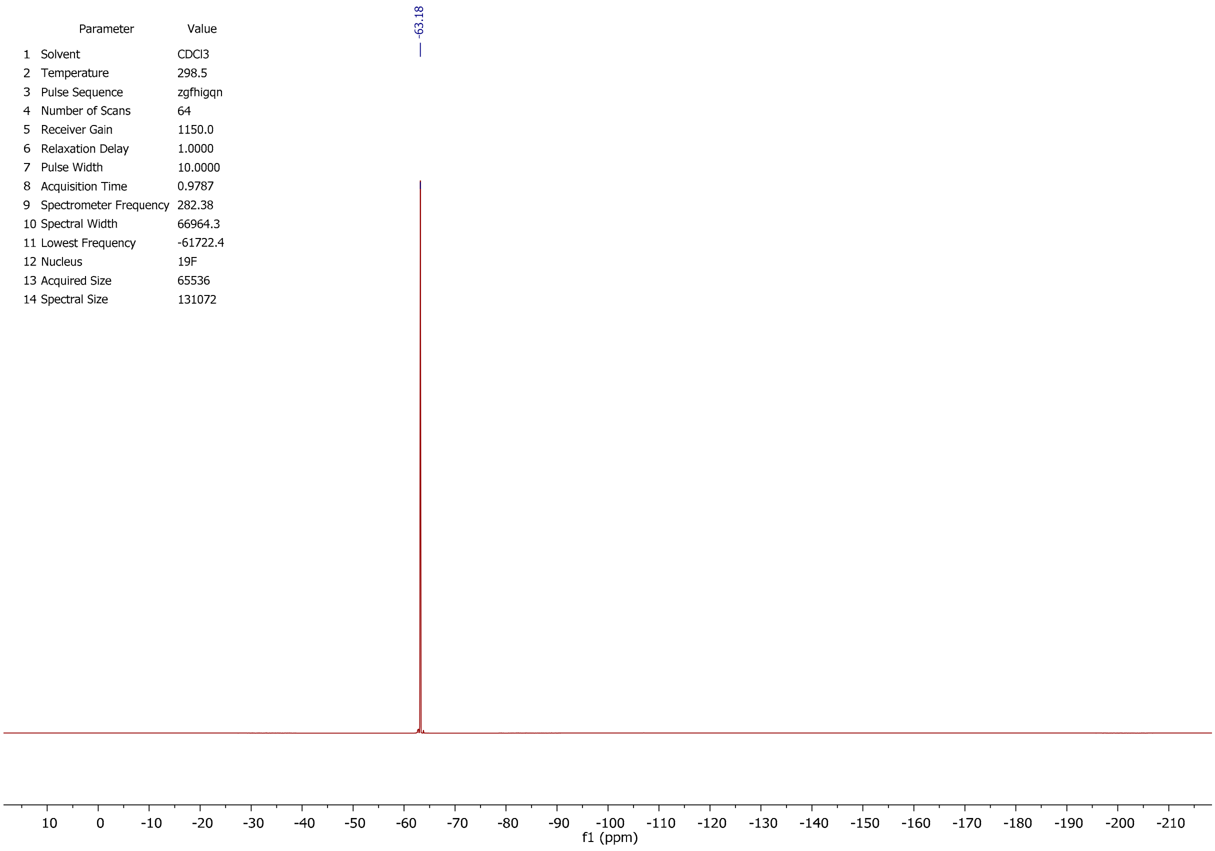

	Parameter	Value
1	Solvent	CDCl3
2	Temperature	298.5
3	Pulse Sequence	zgfhigqn
4	Number of Scans	64
5	Receiver Gain	1150.0
6	Relaxation Delay	1.0000
7	Pulse Width	10.0000
8	Acquisition Time	0.9787
9	Spectrometer Frequency	282.38
10	Spectral Width	66964.3
11	Lowest Frequency	-61722.4
12	Nucleus	19F
13	Acquired Size	65536
14	Spectral Size	131072

5,13-diphenylpyrimido[4',5',6':9,1]pyrrolo[2',1',5':4,5,6]quinolizino[3,2-*b*]quinoline (5a)

Parameter	Value
1 Solvent	CDCl3
2 Temperature	298.1
3 Pulse Sequence	zg30
4 Number of Scans	16
5 Receiver Gain	101.0
6 Relaxation Delay	1.0000
7 Pulse Width	8.0000
8 Acquisition Time	3.2768
9 Spectrometer Frequency	500.13
10 Spectral Width	10000.0
11 Lowest Frequency	-1924.1
12 Nucleus	1H
13 Acquired Size	32768
14 Spectral Size	65536

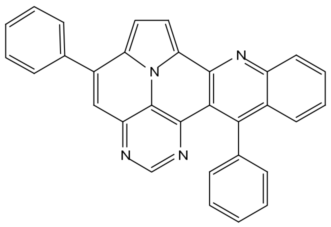

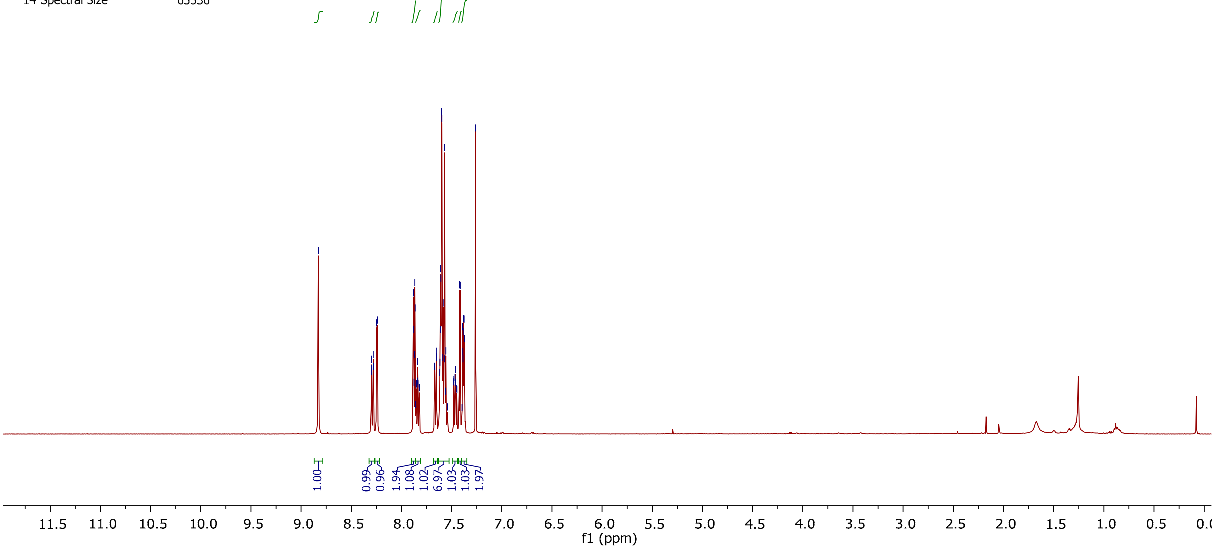

Parameter	Value
1 Solvent	CDCl3
2 Temperature	298.2
3 Pulse Sequence	zgpg30
4 Number of Scans	2048
5 Receiver Gain	101.0
6 Relaxation Delay	2.0000
7 Pulse Width	10.0000
8 Acquisition Time	1.0879
9 Spectrometer Frequency	125.77
10 Spectral Width	30120.5
11 Lowest Frequency	-2484.5
12 Nucleus	13C
13 Acquired Size	32768
14 Spectral Size	65536

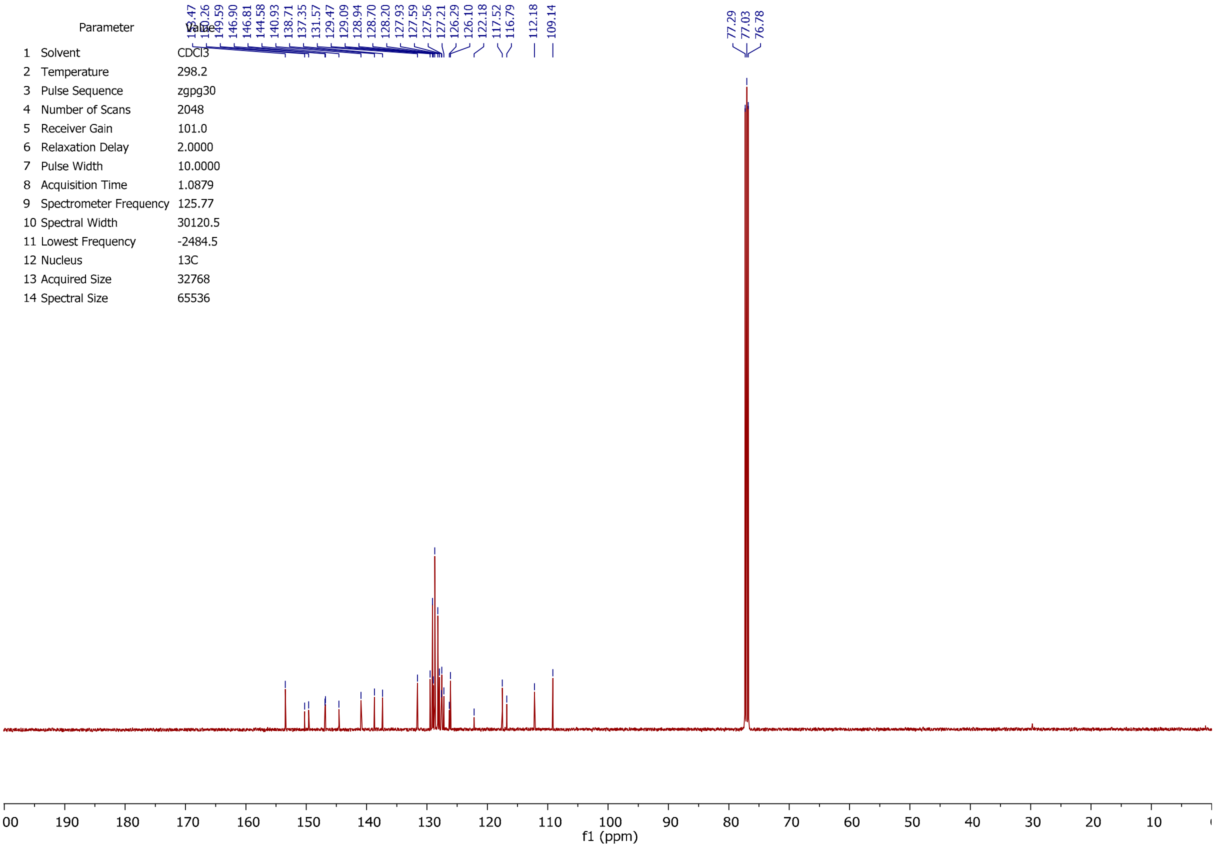

5,13-di-*p*-tolylpyrimido[4',5',6':9,1]pyrrolo[2',1',5':4,5,6]quinolizino[3,2-*b*]quinoline (5b)

	Parameter	Value
1	Solvent	CDCl3
2	Temperature	298.7
3	Pulse Sequence	zg30
4	Number of Scans	16
5	Receiver Gain	362.0
6	Relaxation Delay	1.0000
7	Pulse Width	10.0000
8	Acquisition Time	6.3439
9	Spectrometer Frequency	250.13
10	Spectral Width	5165.3
11	Lowest Frequency	-1038.6
12	Nucleus	1H
13	Acquired Size	32768
14	Spectral Size	65536

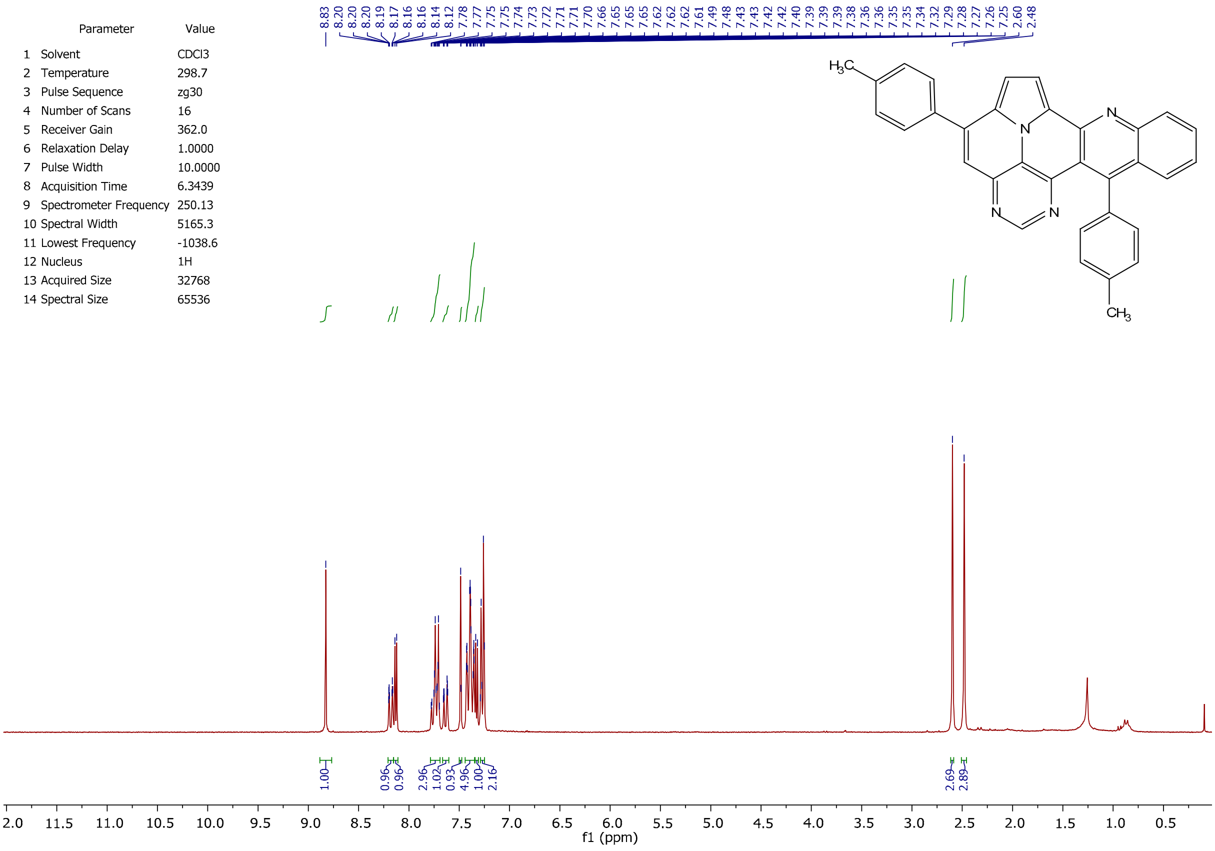

	Parameter	Value
1	Solvent	CDCl3
2	Temperature	299.0
3	Pulse Sequence	zgpg30
4	Number of Scans	2048
5	Receiver Gain	2050.0
6	Relaxation Delay	2.0000
7	Pulse Width	10.0000
8	Acquisition Time	2.1846
9	Spectrometer Frequency	62.90
10	Spectral Width	15000.0
11	Lowest Frequency	-1212.5
12	Nucleus	13C
13	Acquired Size	32768
14	Spectral Size	65536

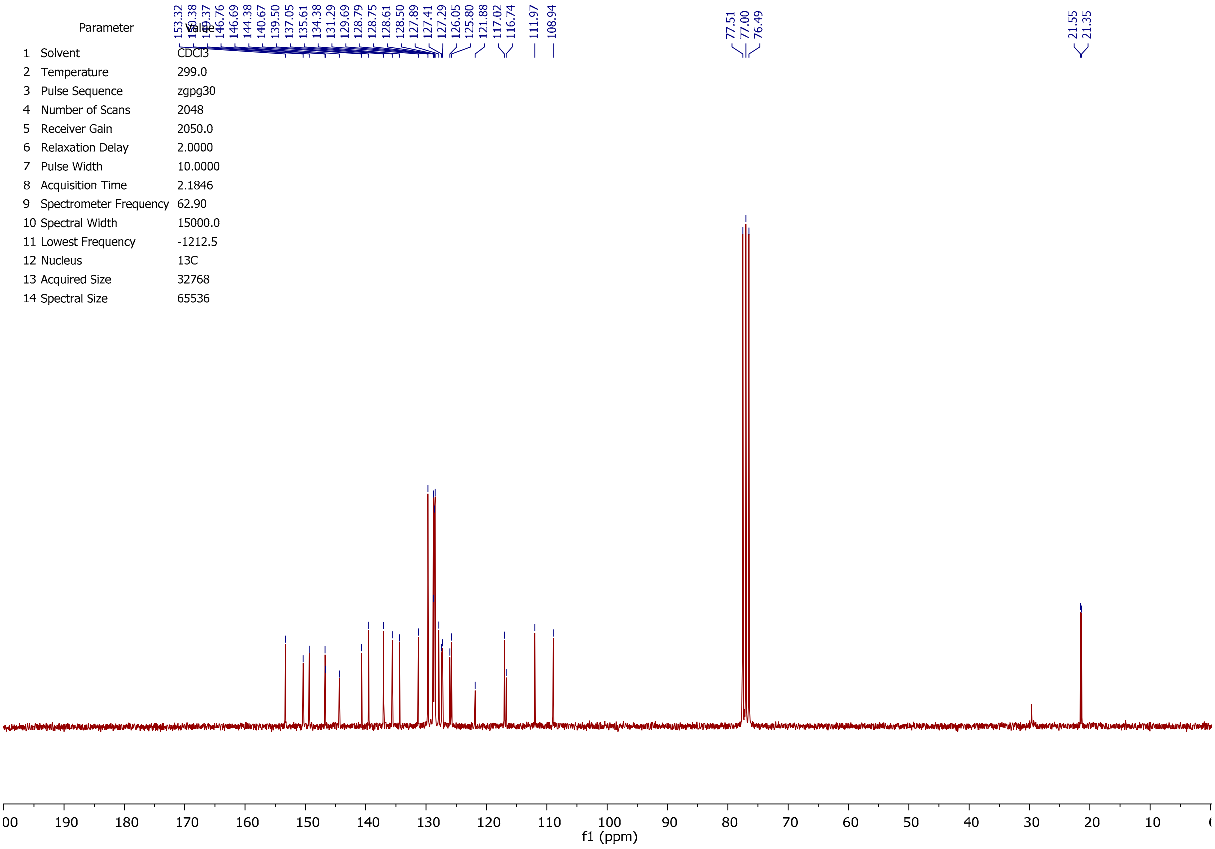

5,13-bis(4-fluorophenyl)pyrimido[4',5',6':9,1]pyrrolo[2',1',5':4,5,6]quinolizino[3,2-*b*]quinoline (5c)

	Parameter	Value
1	Solvent	CDCl3
2	Temperature	298.2
3	Pulse Sequence	zg30
4	Number of Scans	32
5	Receiver Gain	101.0
6	Relaxation Delay	1.0000
7	Pulse Width	8.0000
8	Acquisition Time	3.2768
9	Spectrometer Frequency	500.13
10	Spectral Width	10000.0
11	Lowest Frequency	-1923.2
12	Nucleus	1H
13	Acquired Size	32768
14	Spectral Size	65536

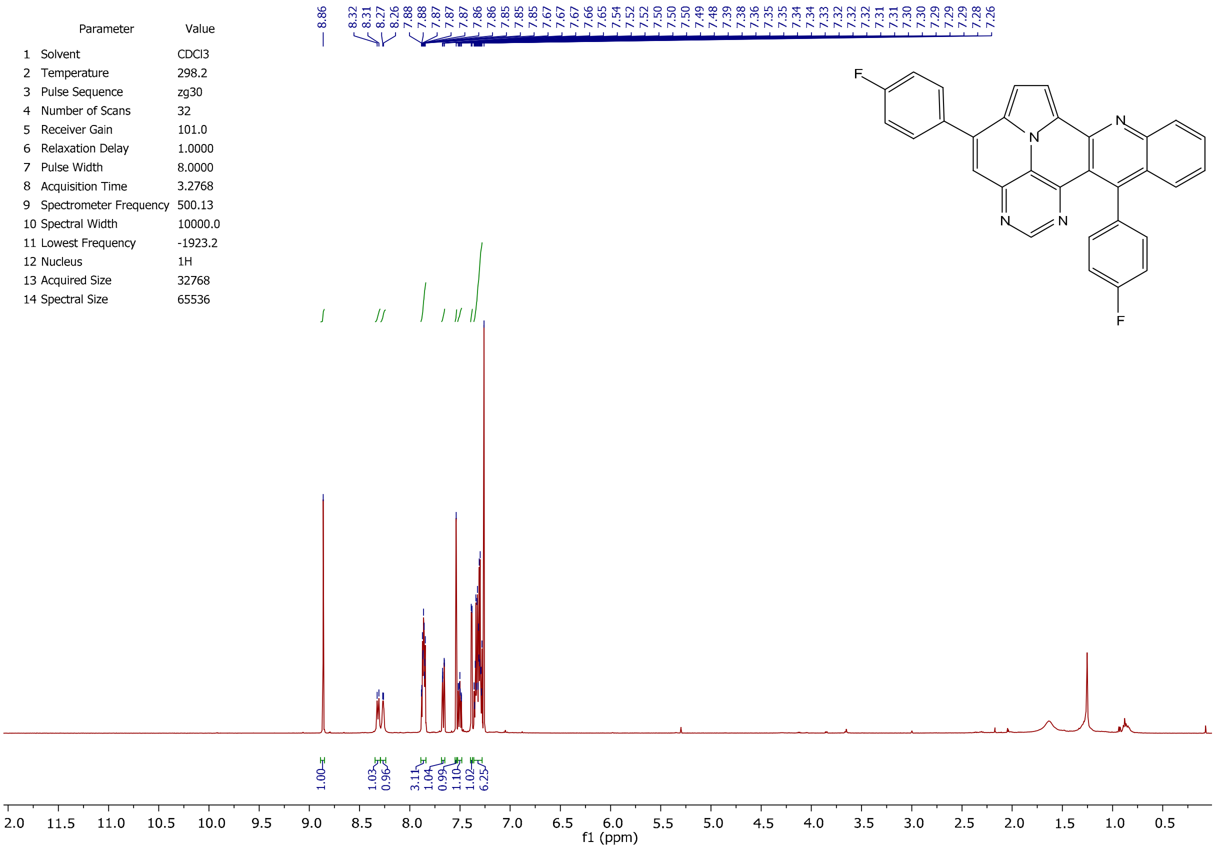

	Parameter	Value
1	Solvent	CDCl3
2	Temperature	298.1
3	Pulse Sequence	zgpg30
4	Number of Scans	4096
5	Receiver Gain	101.0
6	Relaxation Delay	2.0000
7	Pulse Width	10.0000
8	Acquisition Time	1.0879
9	Spectrometer Frequency	125.77
10	Spectral Width	30120.5
11	Lowest Frequency	-2487.5
12	Nucleus	13C
13	Acquired Size	32768
14	Spectral Size	65536

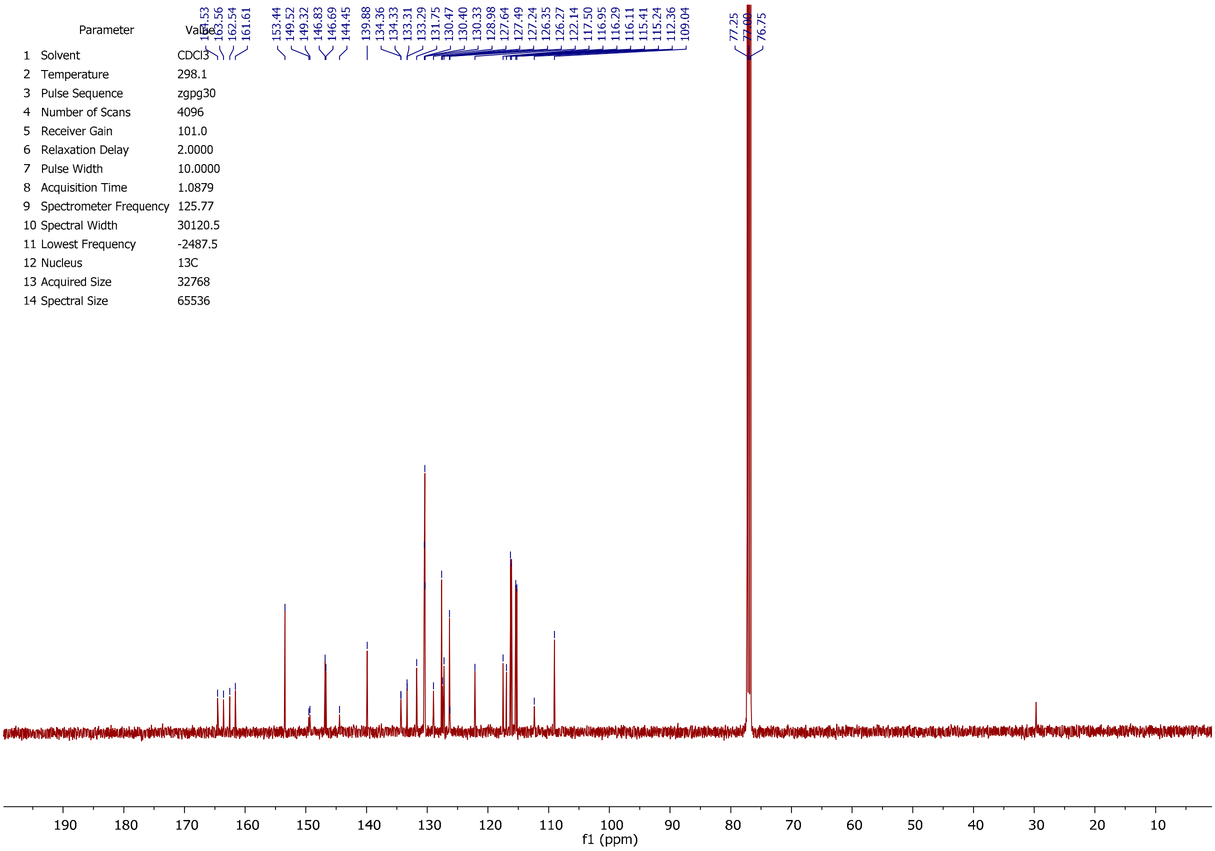

	Parameter	Value
1	Solvent	CDCl3
2	Temperature	298.2
3	Pulse Sequence	zgig
4	Number of Scans	16
5	Receiver Gain	101.0
6	Relaxation Delay	1.0000
7	Pulse Width	12.0000
8	Acquisition Time	0.5767
9	Spectrometer Frequency	470.55
10	Spectral Width	113636.4
11	Lowest Frequency	-103877.4
12	Nucleus	19F
13	Acquired Size	65536
14	Spectral Size	131072

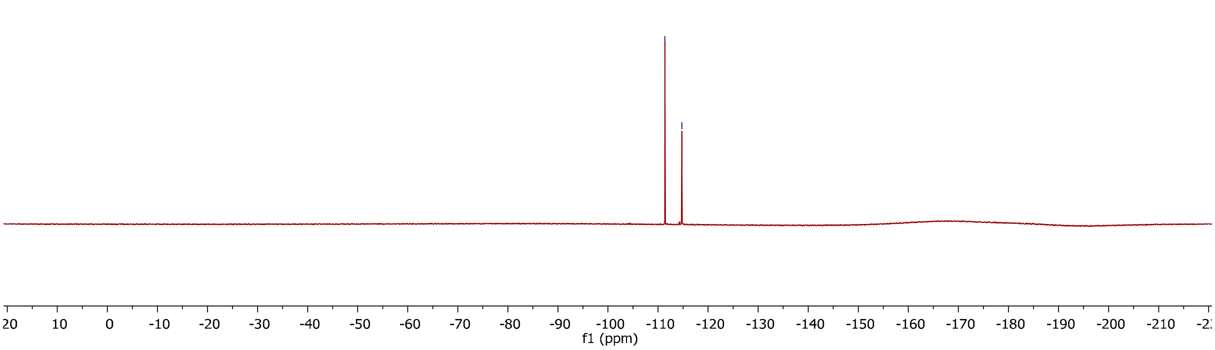

5,13-bis(4-methoxyphenyl)pyrimido[4',5',6':9,1]pyrrolo[2',1',5':4,5,6]quinolizino[3,2-*b*]quinoline (5e)

	Parameter	Value
1	Solvent	CDCl3/TFA
2	Temperature	298.2
3	Pulse Sequence	zg30
4	Number of Scans	16
5	Receiver Gain	362.0
6	Relaxation Delay	1.0000
7	Pulse Width	10.0000
8	Acquisition Time	6.3439
9	Spectrometer Frequency	250.13
10	Spectral Width	5165.3
11	Lowest Frequency	-1038.5
12	Nucleus	1H
13	Acquired Size	32768
14	Spectral Size	65536

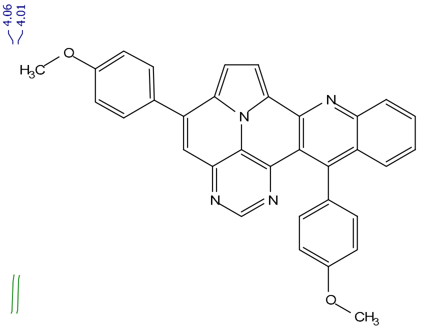

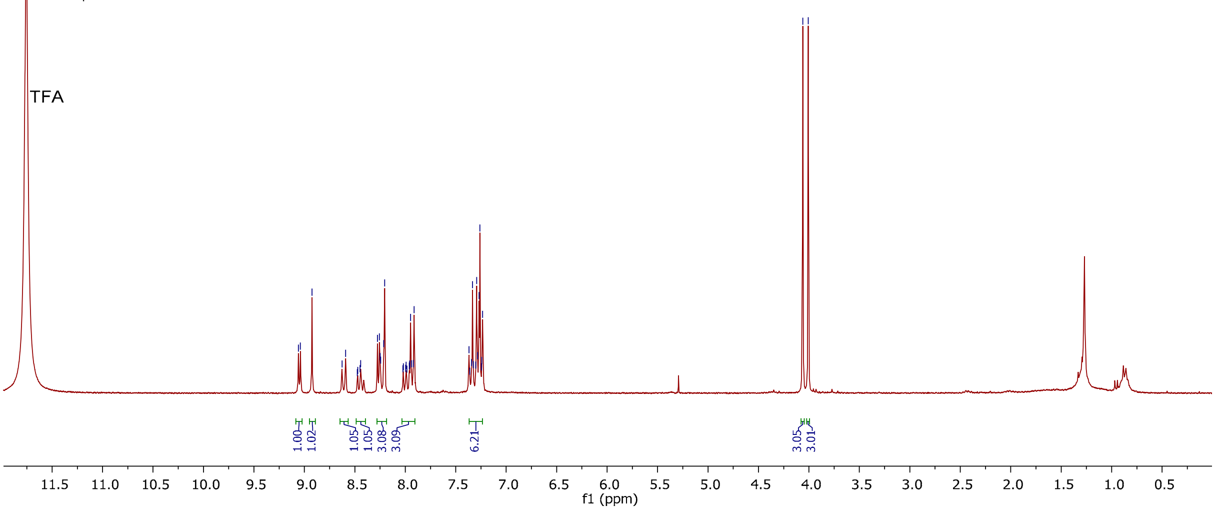

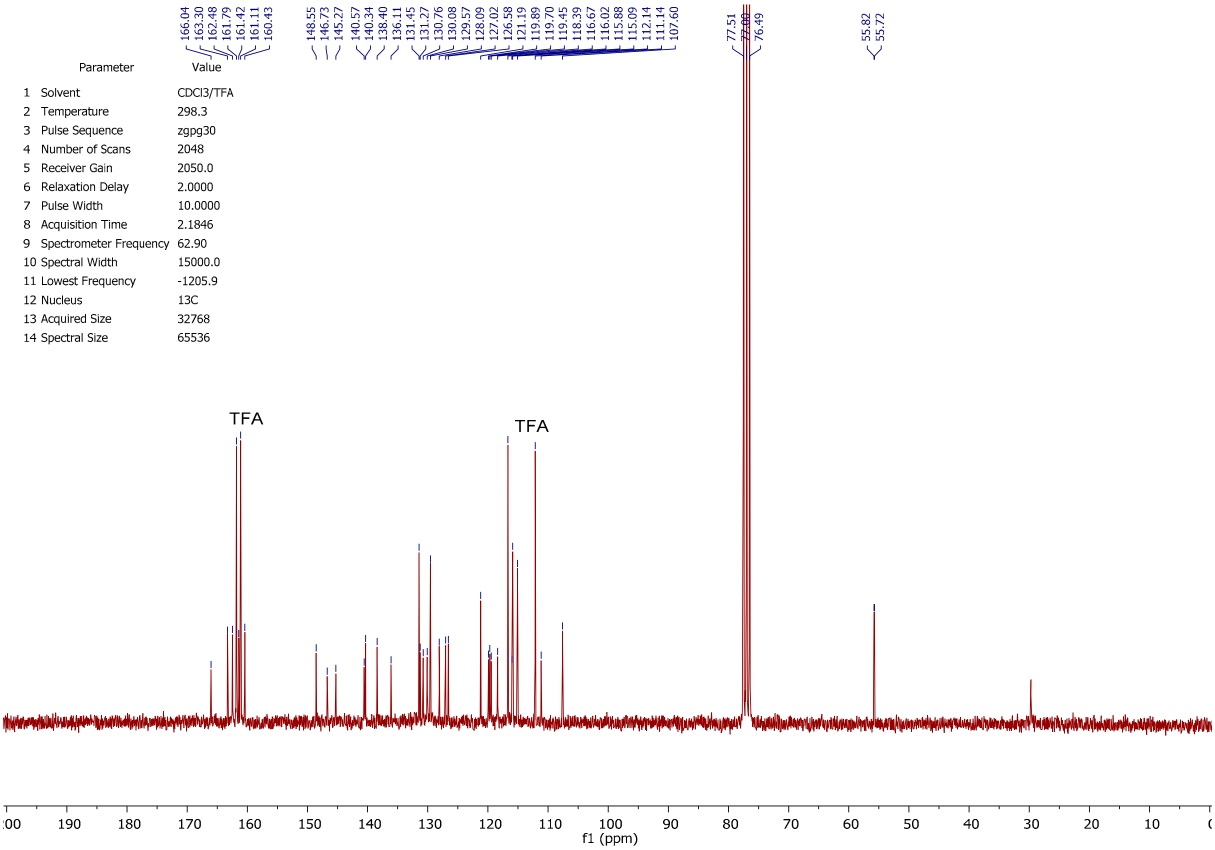

	Parameter	Value
1	Solvent	CDCl3/TFA
2	Temperature	298.3
3	Pulse Sequence	zgpg30
4	Number of Scans	2048
5	Receiver Gain	2050.0
6	Relaxation Delay	2.0000
7	Pulse Width	10.0000
8	Acquisition Time	2.1846
9	Spectrometer Frequency	62.90
10	Spectral Width	15000.0
11	Lowest Frequency	-1205.9
12	Nucleus	13C
13	Acquired Size	32768
14	Spectral Size	65536

11-methyl-5,13-diphenylpyrimido[4',5',6':9,1]pyrrolo[2',1',5':4,5,6]quinolizino[3,2-*b*]quinoline (5g)

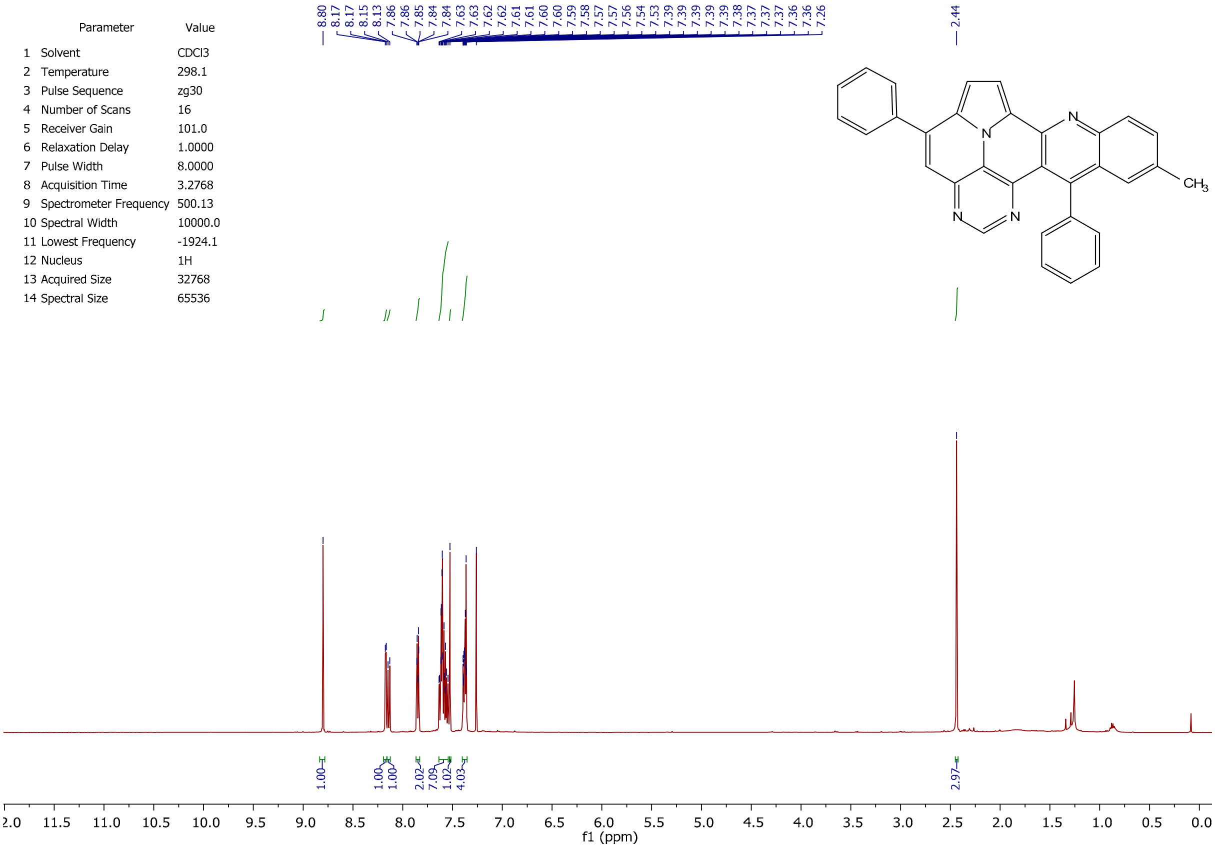

	Parameter	Value
1	Solvent	CDCl3
2	Temperature	298.1
3	Pulse Sequence	zg30
4	Number of Scans	16
5	Receiver Gain	101.0
6	Relaxation Delay	1.0000
7	Pulse Width	8.0000
8	Acquisition Time	3.2768
9	Spectrometer Frequency	500.13
10	Spectral Width	10000.0
11	Lowest Frequency	-1924.1
12	Nucleus	1H
13	Acquired Size	32768
14	Spectral Size	65536

Parameter	Value
1 Solvent	CDCl3
2 Temperature	298.2
3 Pulse Sequence	zgpg30
4 Number of Scans	1024
5 Receiver Gain	101.0
6 Relaxation Delay	2.0000
7 Pulse Width	10.0000
8 Acquisition Time	1.0879
9 Spectrometer Frequency	125.77
10 Spectral Width	30120.5
11 Lowest Frequency	-2490.1
12 Nucleus	13C
13 Acquired Size	32768
14 Spectral Size	65536

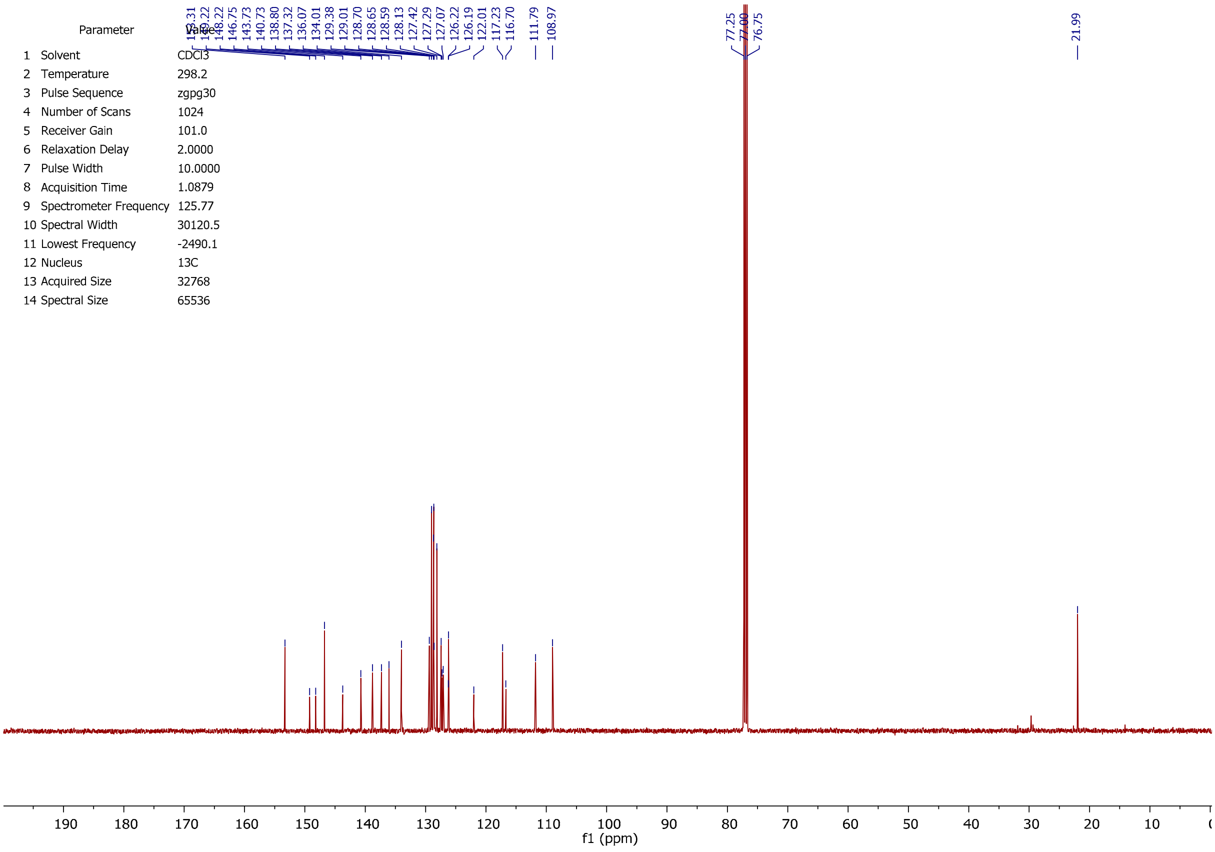

9-methyl-5,13-diphenylpyrimido[4',5',6':9,1]pyrrolo[2',1',5':4,5,6]quinolizino[3,2-*b*]quinoline (5h)

Parameter	Value
1 Solvent	CDCl3
2 Temperature	298.2
3 Pulse Sequence	zg30
4 Number of Scans	16
5 Receiver Gain	287.0
6 Relaxation Delay	2.0000
7 Pulse Width	10.0000
8 Acquisition Time	5.2954
9 Spectrometer Frequency	300.13
10 Spectral Width	6188.1
11 Lowest Frequency	-1246.5
12 Nucleus	1H
13 Acquired Size	32768
14 Spectral Size	65536

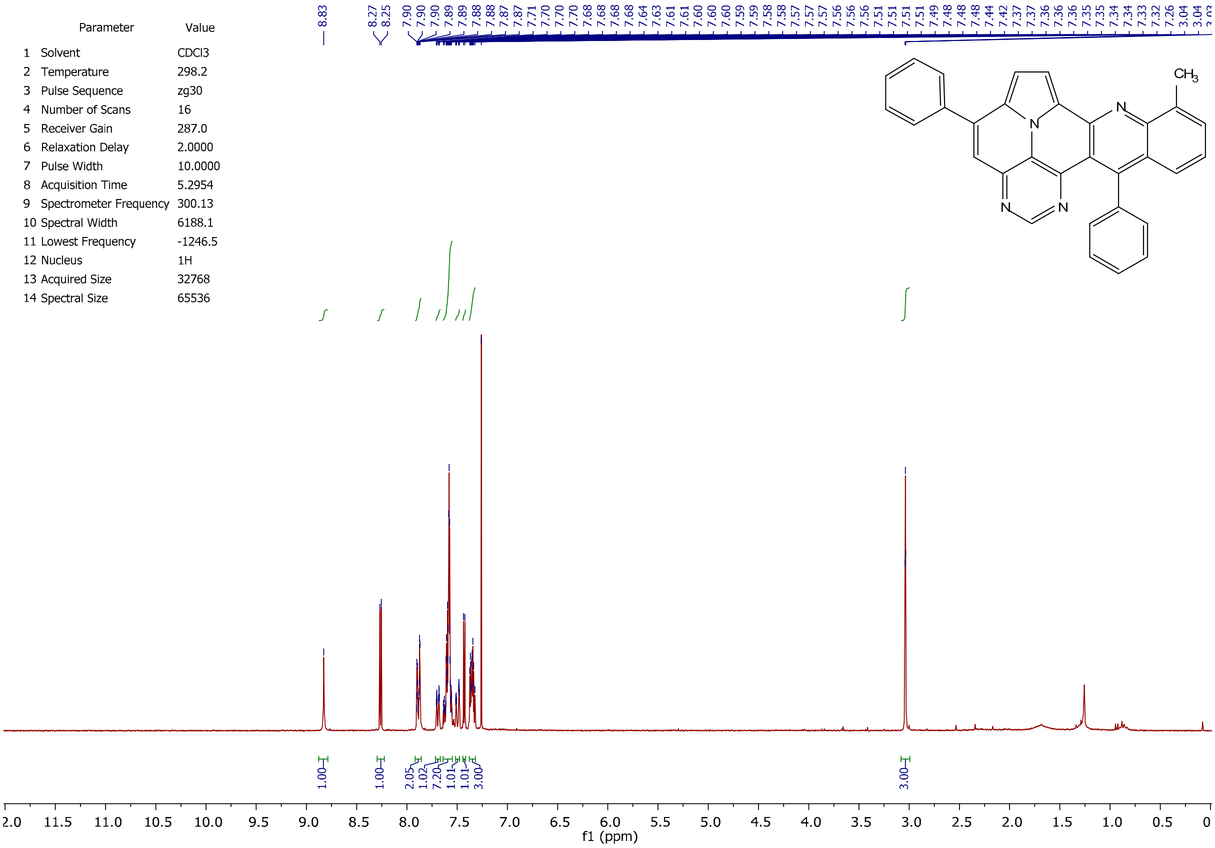

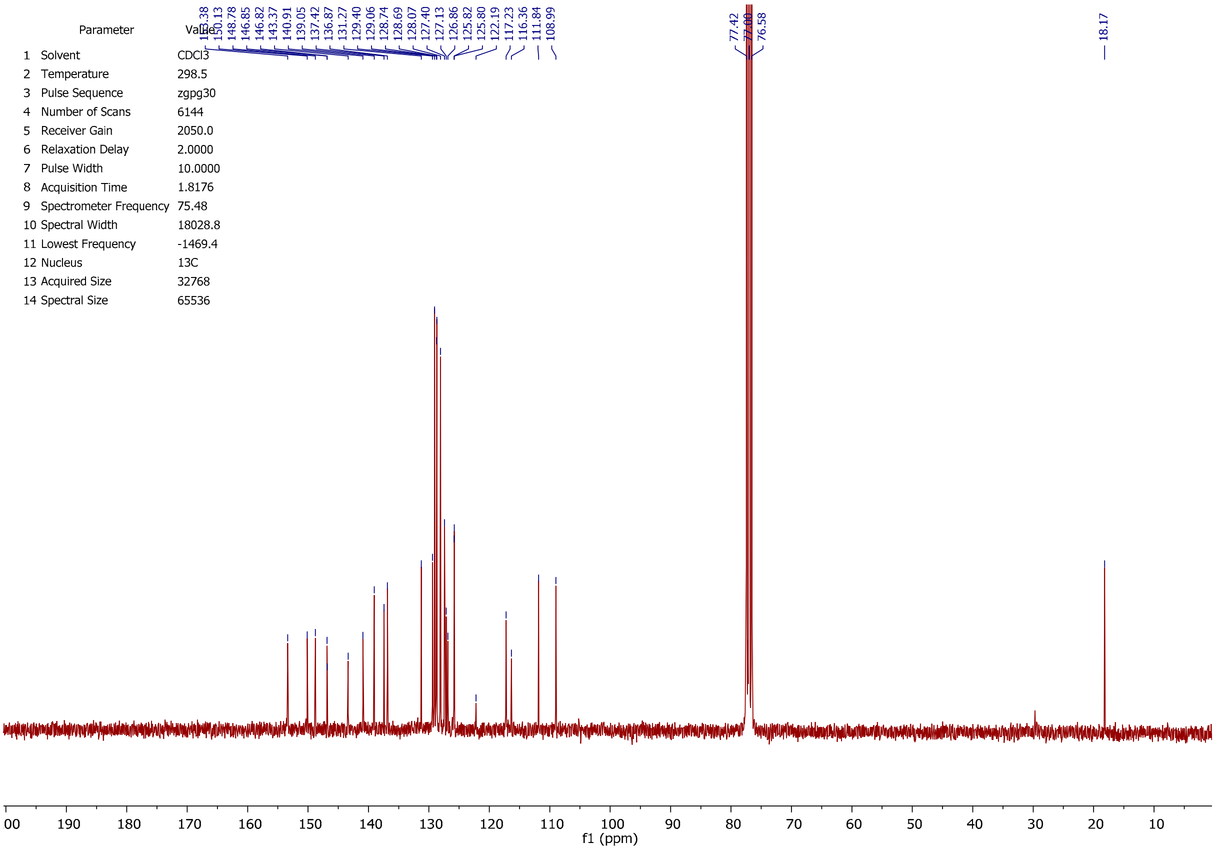

	Parameter	Value
1	Solvent	CDCl3
2	Temperature	298.5
3	Pulse Sequence	zgpg30
4	Number of Scans	6144
5	Receiver Gain	2050.0
6	Relaxation Delay	2.0000
7	Pulse Width	10.0000
8	Acquisition Time	1.8176
9	Spectrometer Frequency	75.48
10	Spectral Width	18028.8
11	Lowest Frequency	-1469.4
12	Nucleus	13C
13	Acquired Size	32768
14	Spectral Size	65536

10,12-dimethyl-5,13-diphenylpyrimido[4',5',6':9,1]pyrrolo[2',1',5':4,5,6]quinolizino[3,2-*b*]quinoline (5i)

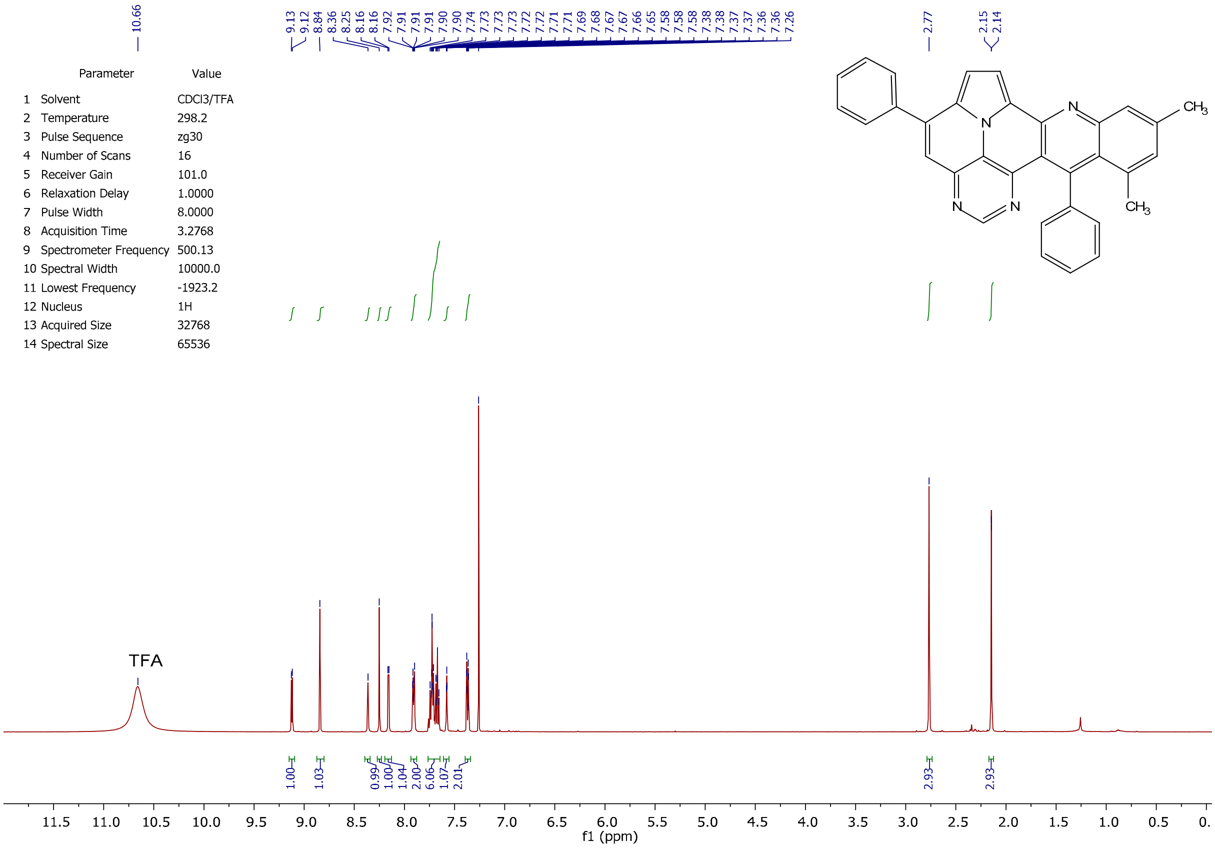

	Parameter	Value
1	Solvent	CDCl3/TFA
2	Temperature	298.2
3	Pulse Sequence	zg30
4	Number of Scans	16
5	Receiver Gain	101.0
6	Relaxation Delay	1.0000
7	Pulse Width	8.0000
8	Acquisition Time	3.2768
9	Spectrometer Frequency	500.13
10	Spectral Width	10000.0
11	Lowest Frequency	-1923.2
12	Nucleus	1H
13	Acquired Size	32768
14	Spectral Size	65536

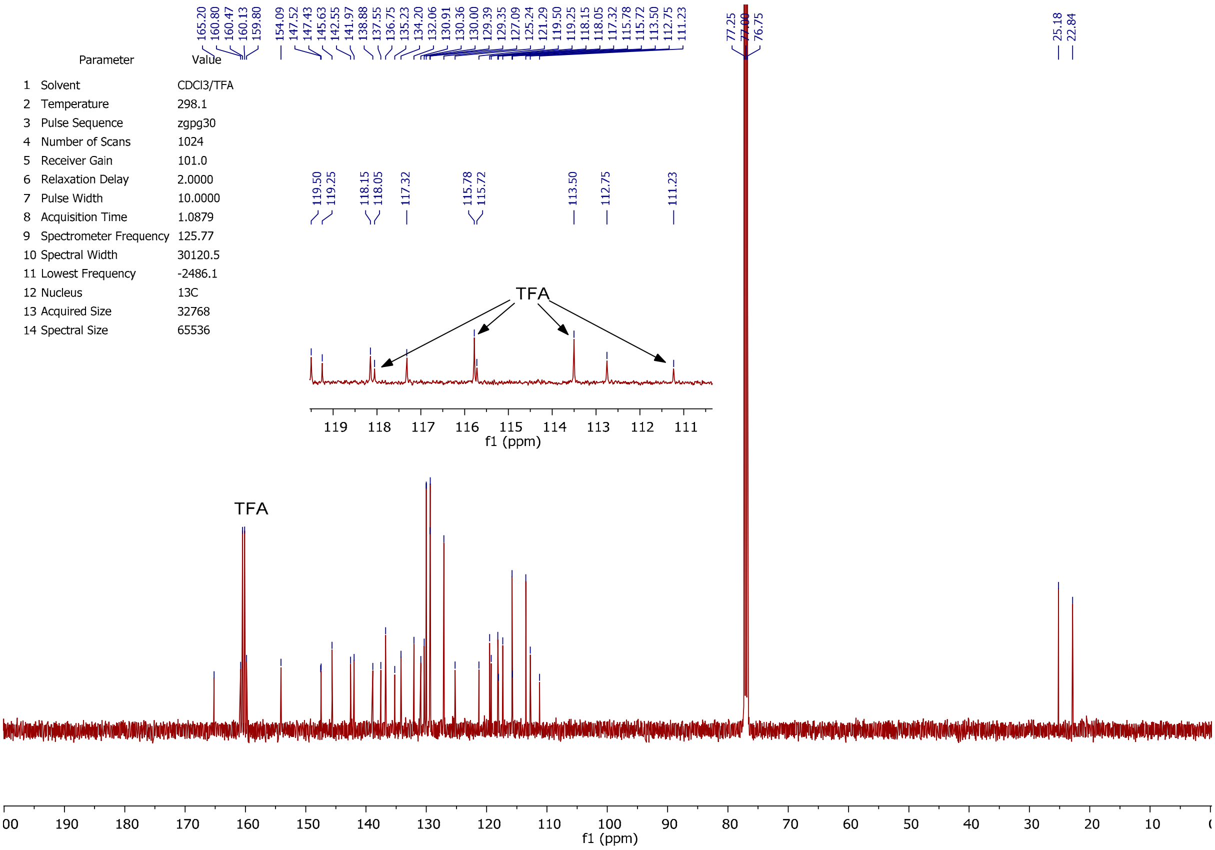

	Parameter	Value
1	Solvent	CDCl3/TFA
2	Temperature	298.1
3	Pulse Sequence	zgpg30
4	Number of Scans	1024
5	Receiver Gain	101.0
6	Relaxation Delay	2.0000
7	Pulse Width	10.0000
8	Acquisition Time	1.0879
9	Spectrometer Frequency	125.77
10	Spectral Width	30120.5
11	Lowest Frequency	-2486.1
12	Nucleus	13C
13	Acquired Size	32768
14	Spectral Size	65536

11-fluoro-5,13-diphenylpyrimido[4',5',6':9,1]pyrrolo[2',1',5':4,5,6]quinolizino[3,2-*b*]quinoline (5j)

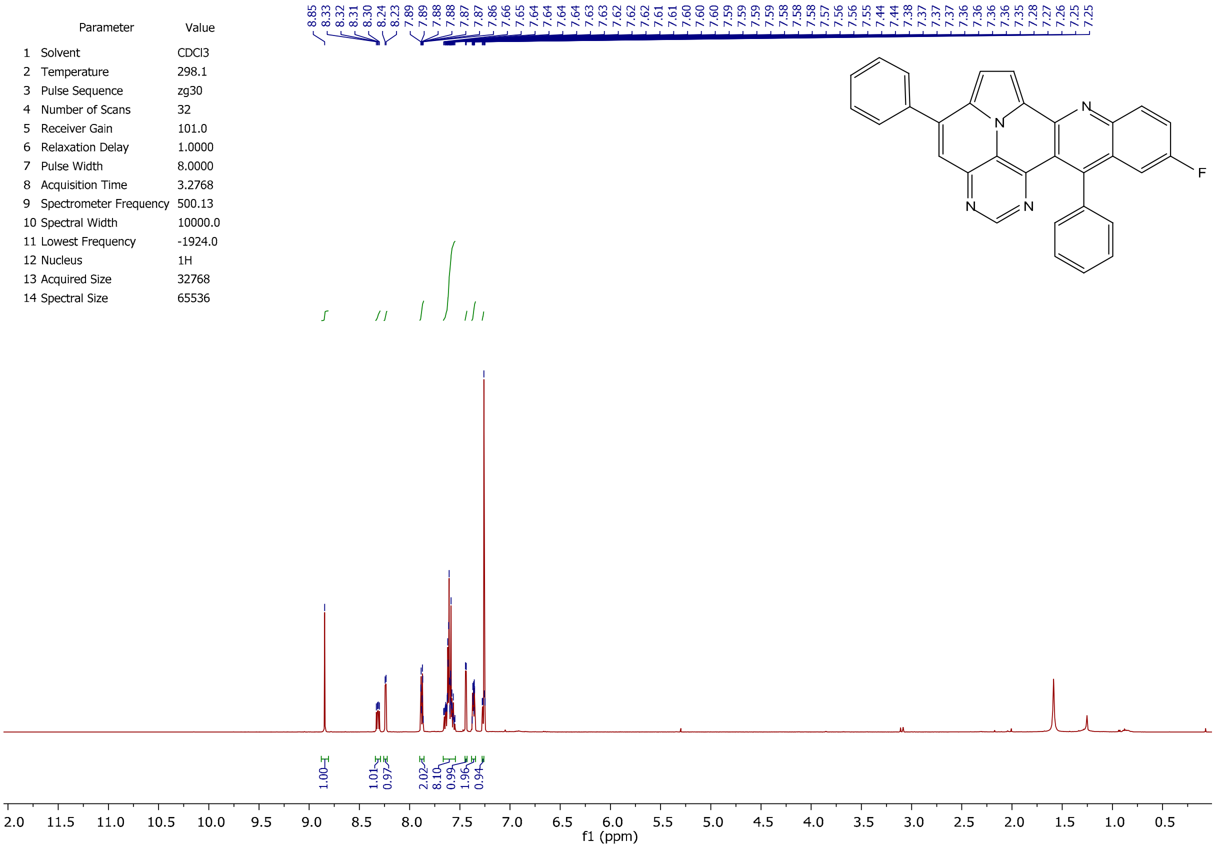

	Parameter	Value
1	Solvent	CDCl3
2	Temperature	298.1
3	Pulse Sequence	zg30
4	Number of Scans	32
5	Receiver Gain	101.0
6	Relaxation Delay	1.0000
7	Pulse Width	8.0000
8	Acquisition Time	3.2768
9	Spectrometer Frequency	500.13
10	Spectral Width	10000.0
11	Lowest Frequency	-1924.0
12	Nucleus	1H
13	Acquired Size	32768
14	Spectral Size	65536

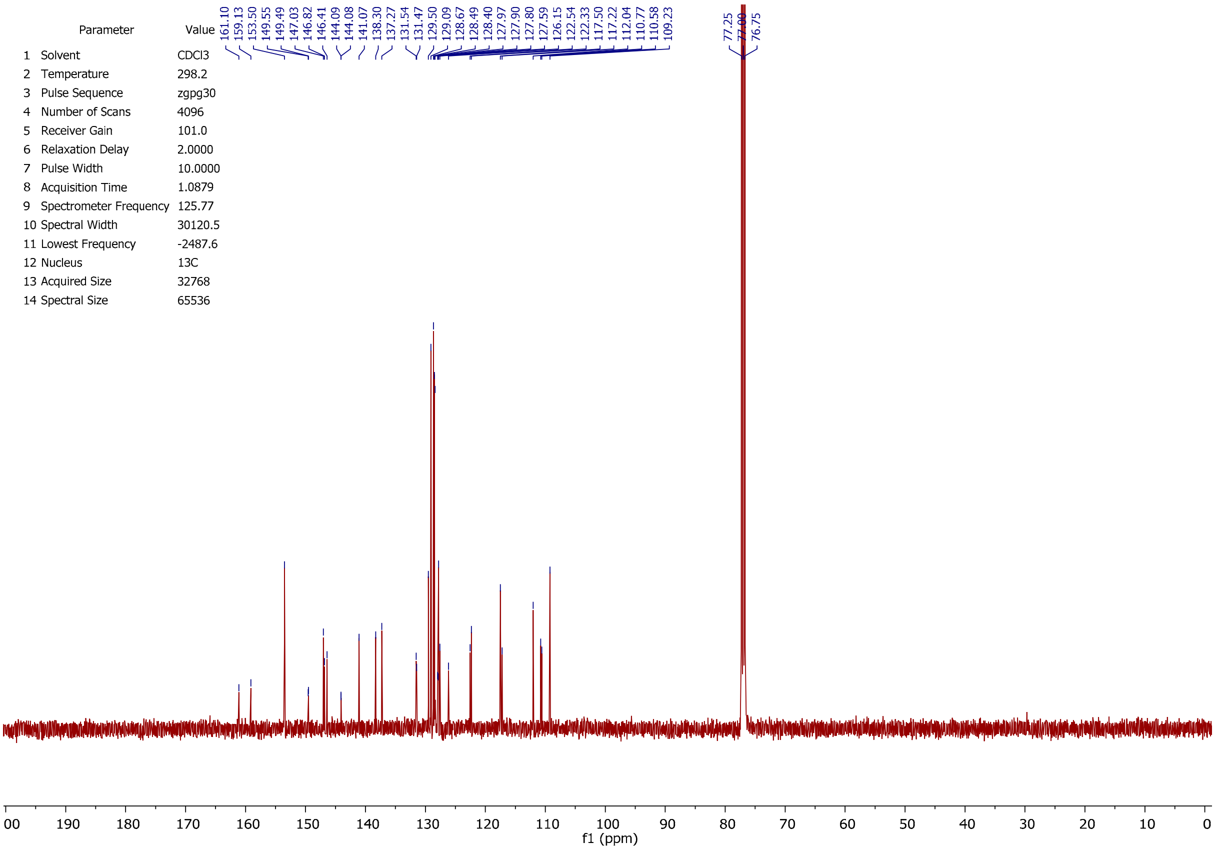

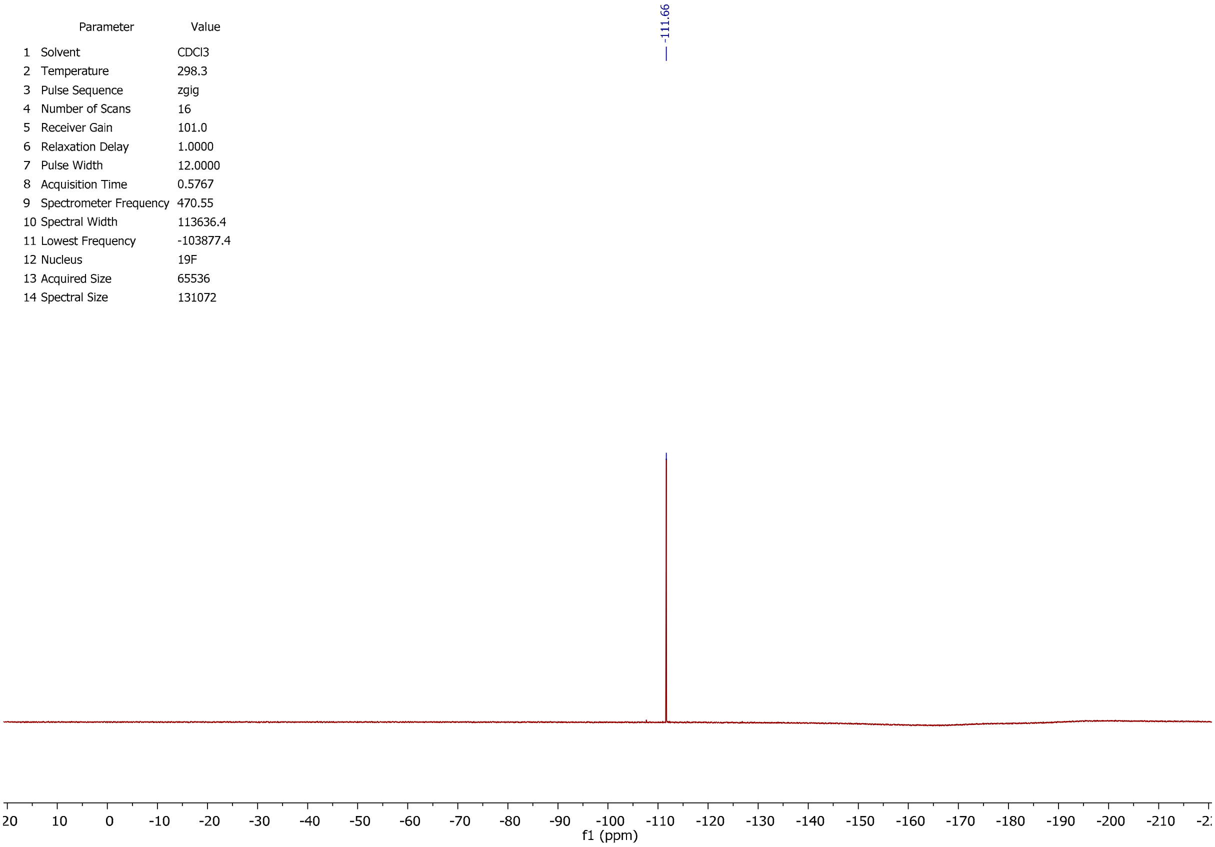

***N*,*N*-dimethyl-5,13-diphenylpyrimido[4',5',6':9,1]pyrrolo[2',1',5':4,5,6]quinolizino[3,2-*b*]quinolin-11-amine (5k)**

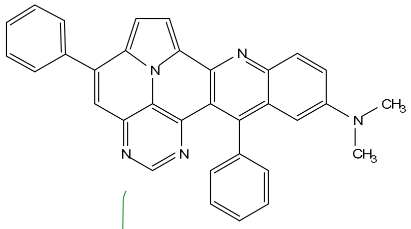

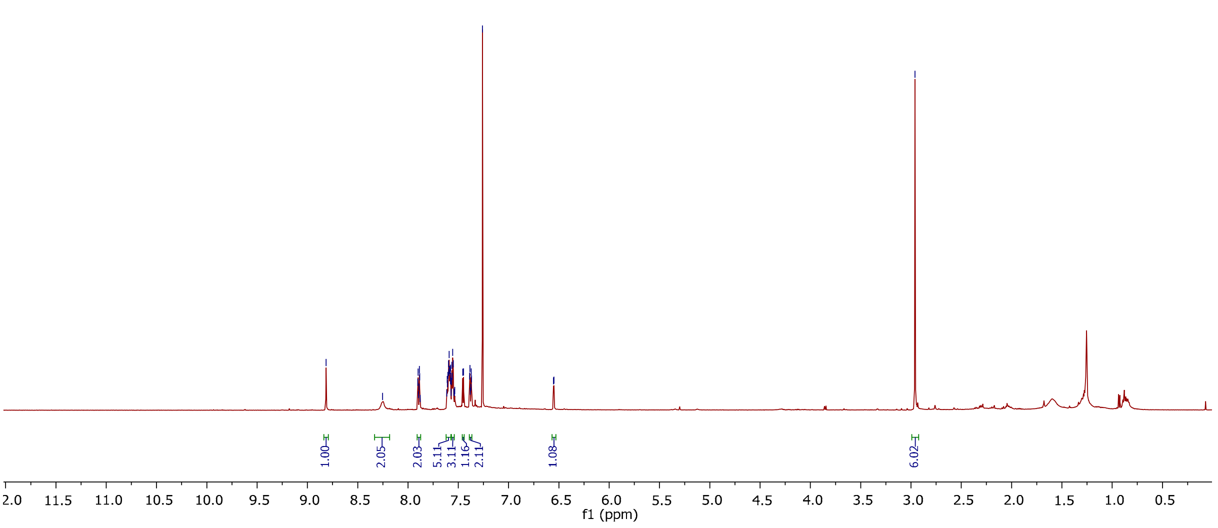

	Parameter	Value
1	Solvent	CDCl3
2	Temperature	298.2
3	Pulse Sequence	zg30
4	Number of Scans	16
5	Receiver Gain	101.0
6	Relaxation Delay	1.0000
7	Pulse Width	8.0000
8	Acquisition Time	3.2768
9	Spectrometer Frequency	500.13
10	Spectral Width	10000.0
11	Lowest Frequency	-1924.1
12	Nucleus	1H
13	Acquired Size	32768
14	Spectral Size	65536

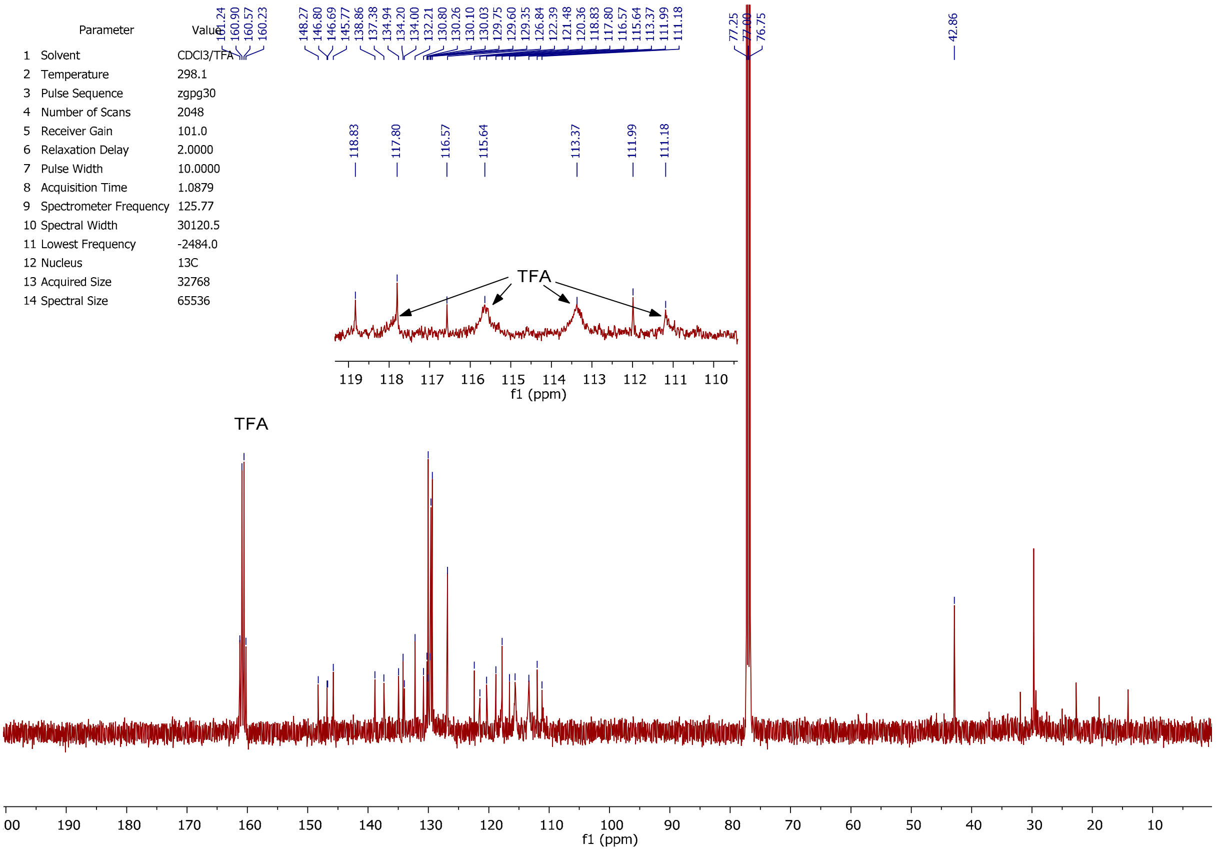

	Parameter	Value
1	Solvent	CDCl3/TFA
2	Temperature	298.1
3	Pulse Sequence	zgpg30
4	Number of Scans	2048
5	Receiver Gain	101.0
6	Relaxation Delay	2.0000
7	Pulse Width	10.0000
8	Acquisition Time	1.0879
9	Spectrometer Frequency	125.77
10	Spectral Width	30120.5
11	Lowest Frequency	-2484.0
12	Nucleus	13C
13	Acquired Size	32768
14	Spectral Size	65536

5,13-diphenyl-11-(trifluoromethyl)pyrimido[4',5',6':9,1]pyrrolo[2',1',5':4,5,6]quinolizino[3,2-*b*]quinoline (5l)

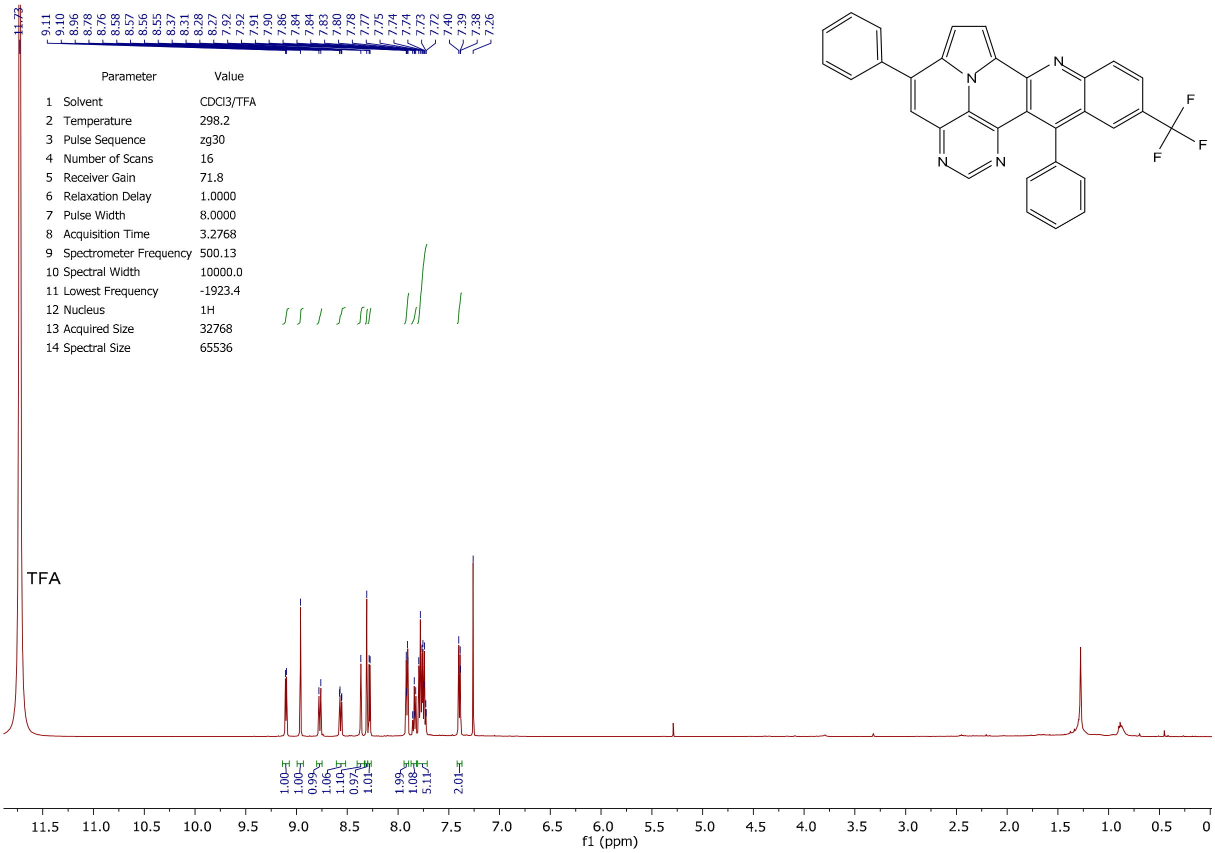
11.73
9.11 9.10 8.96 8.78 8.76 8.58 8.57 8.56 8.55 8.37 8.31 8.28 8.27 7.92 7.92 7.92 7.91 7.90 7.86 7.84 7.84 7.83 7.80 7.78 7.77 7.75 7.74 7.74 7.73 7.72 7.40 7.39 7.38 7.26
Parameter Value
1 Solvent CDCl3/TFA
2 Temperature 298.2
3 Pulse Sequence zg30
4 Number of Scans 16
5 Receiver Gain 71.8
6 Relaxation Delay 1.0000
7 Pulse Width 8.0000
8 Acquisition Time 3.2768
9 Spectrometer Frequency 500.13
10 Spectral Width 10000.0
11 Lowest Frequency -1923.4
12 Nucleus 1H
13 Acquired Size 32768
14 Spectral Size 65536
N
N
N
N
F
F
F
TFA
1.00 1.00 0.99 1.06 1.10 0.97 1.01 1.99 1.08 5.11 2.01
11.5 11.0 10.5 10.0 9.5 9.0 8.5 8.0 7.5 7.0 6.5 6.0 5.5 5.0 4.5 4.0 3.5 3.0 2.5 2.0 1.5 1.0 0.5 0
f1 (ppm)

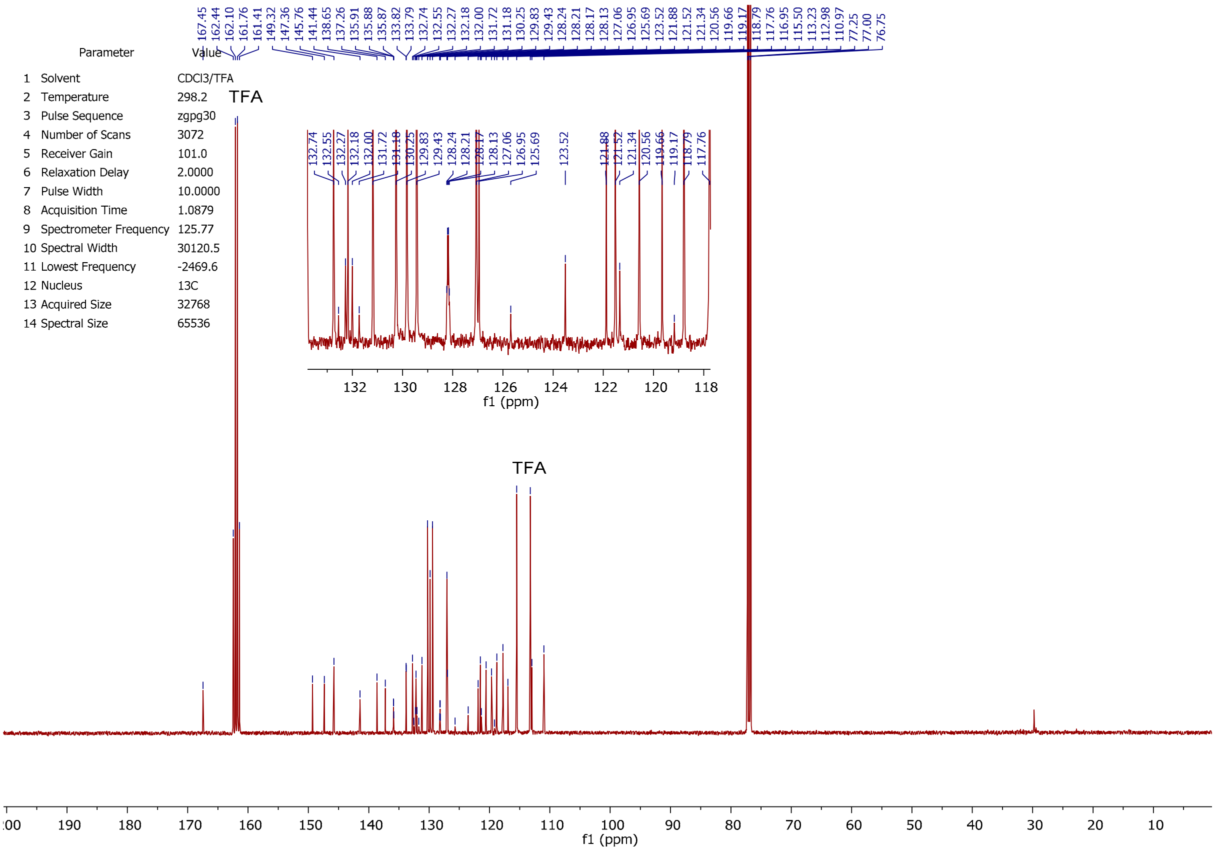
167.45 162.44 162.10 161.76 161.41 149.32 147.36 145.76 141.44 138.65 137.26 135.91 135.88 133.82 133.79 132.74 132.55 132.27 132.18 132.00 131.72 131.18 130.25 129.83 129.43 128.24 128.21 128.17 128.13 127.06 126.95 125.69 123.52 121.88 121.52 121.34 120.56 119.66 119.17 118.79 117.76 116.95 115.50 113.23 112.98 110.97 77.25 77.00 76.75
Parameter Value
1 Solvent CDCl3/TFA
2 Temperature 298.2
3 Pulse Sequence zgpg30
4 Number of Scans 3072
5 Receiver Gain 101.0
6 Relaxation Delay 2.0000
7 Pulse Width 10.0000
8 Acquisition Time 1.0879
9 Spectrometer Frequency 125.77
10 Spectral Width 30120.5
11 Lowest Frequency -2469.6
12 Nucleus 13C
13 Acquired Size 32768
14 Spectral Size 65536
TFA
132.74 132.55 132.27 132.18 132.00 131.72 131.18 130.25 129.83 129.43 128.24 128.21 128.17 128.13 127.06 126.95 125.69 123.52 121.88 121.52 121.34 120.56 119.66 119.17 118.79 117.76
132 130 128 126 124 122 120 118
f1 (ppm)
TFA
200 190 180 170 160 150 140 130 120 110 100 90 80 70 60 50 40 30 20 10
f1 (ppm)

	Parameter	Value
1	Solvent	CDCl3/TFA
2	Temperature	298.2
3	Pulse Sequence	zgig
4	Number of Scans	16
5	Receiver Gain	101.0
6	Relaxation Delay	1.0000
7	Pulse Width	12.0000
8	Acquisition Time	0.5767
9	Spectrometer Frequency	470.55
10	Spectral Width	113636.4
11	Lowest Frequency	-103877.4
12	Nucleus	19F
13	Acquired Size	65536
14	Spectral Size	131072

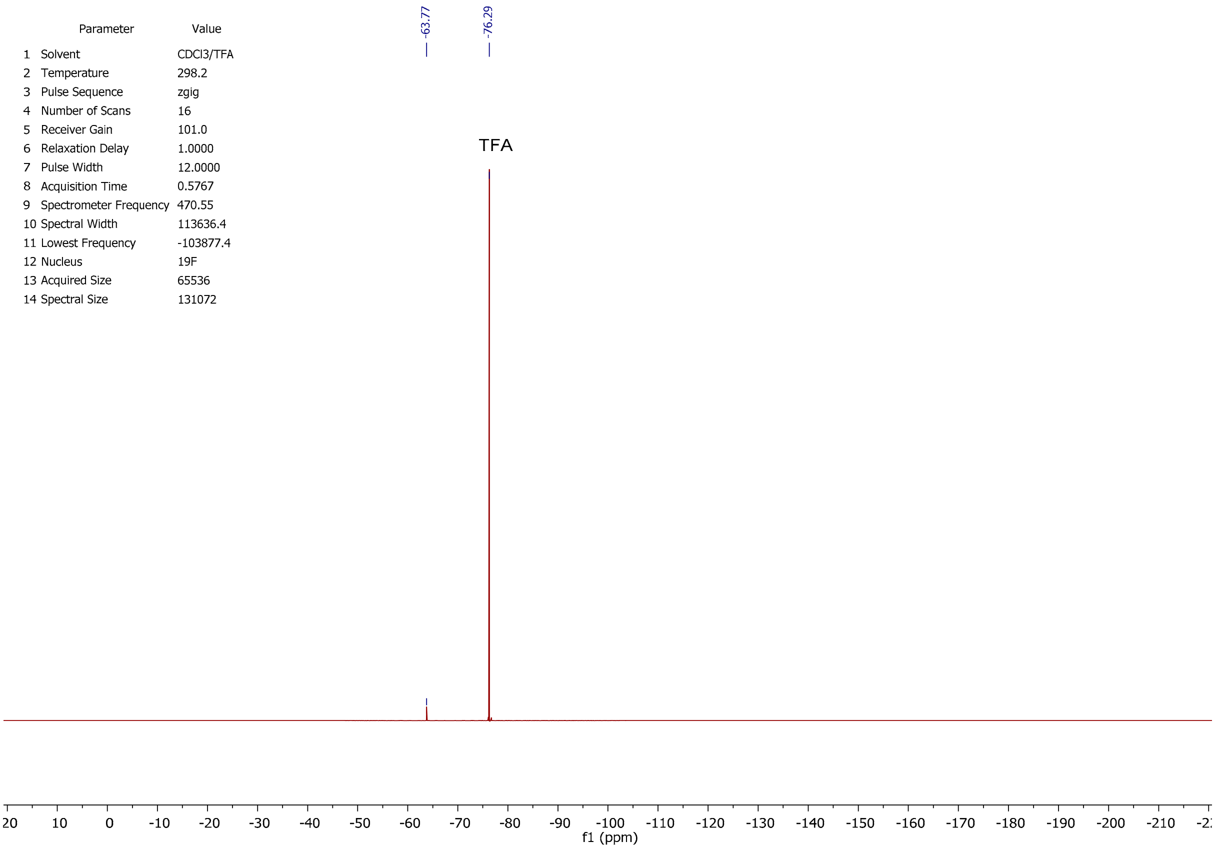

phenyl(8-phenylpyrimido[4,5,6-*ij*]pyrrolo[2,1,5-*de*]quinolizin-4-yl)methanone (6a)

	Parameter	Value
1	Solvent	CDCl3
2	Temperature	298.2
3	Pulse Sequence	zg30
4	Number of Scans	16
5	Receiver Gain	724.0
6	Relaxation Delay	1.0000
7	Pulse Width	10.0000
8	Acquisition Time	6.3439
9	Spectrometer Frequency	250.13
10	Spectral Width	5165.3
11	Lowest Frequency	-1038.7
12	Nucleus	1H
13	Acquired Size	32768
14	Spectral Size	65536

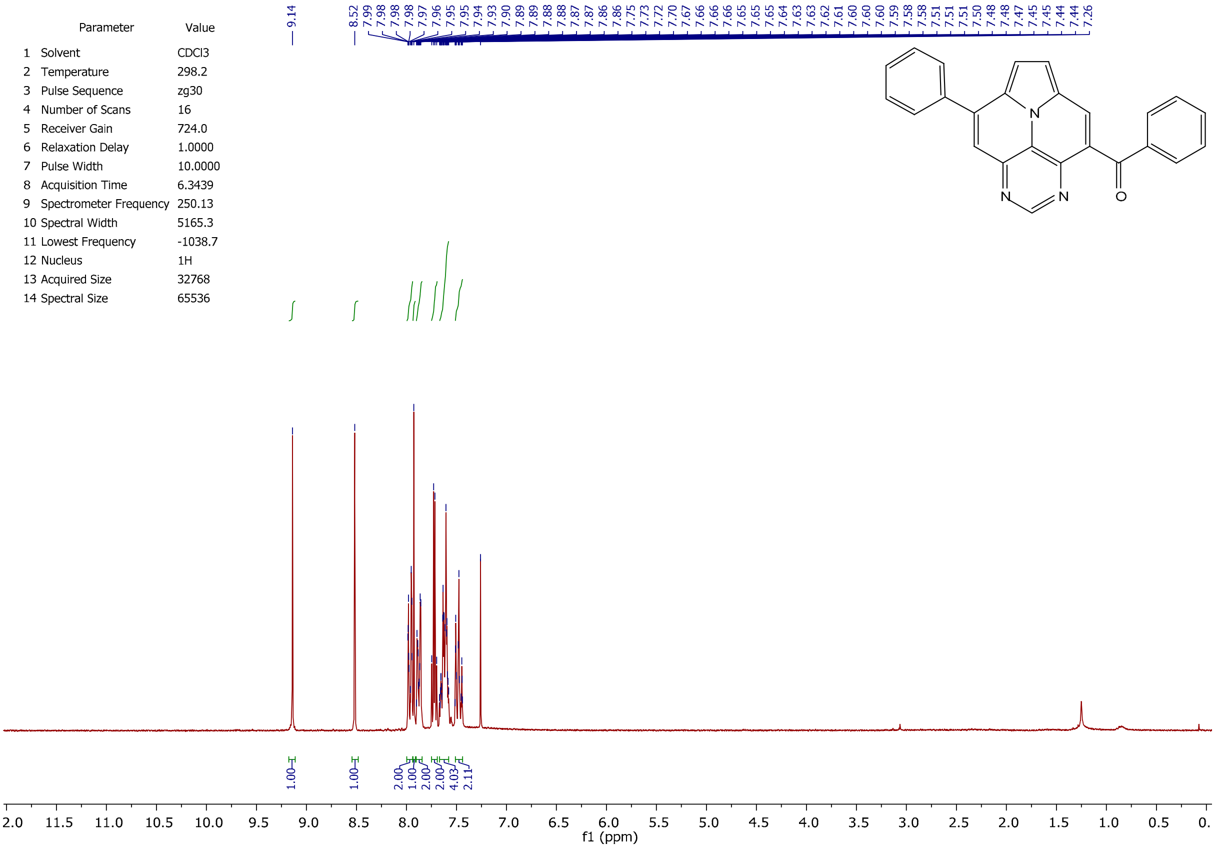

	Parameter	Value
1	Solvent	CDCl3
2	Temperature	298.1
3	Pulse Sequence	zgpg30
4	Number of Scans	1024
5	Receiver Gain	1030.0
6	Relaxation Delay	2.0000
7	Pulse Width	10.0000
8	Acquisition Time	2.1846
9	Spectrometer Frequency	62.90
10	Spectral Width	15000.0
11	Lowest Frequency	-1212.0
12	Nucleus	13C
13	Acquired Size	32768
14	Spectral Size	65536

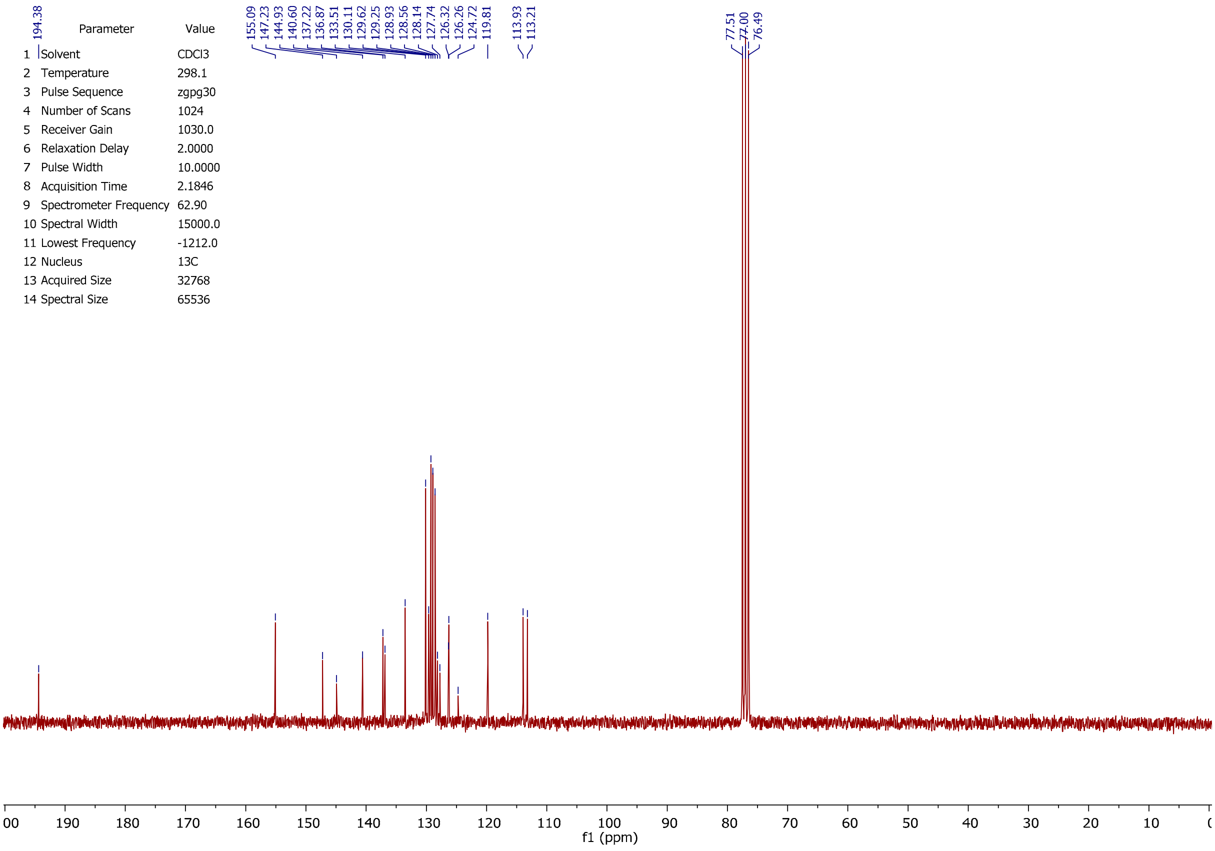

p-tolyl(8-(*p*-tolyl)pyrimido[4,5,6-*ij*]pyrrolo[2,1,5-*de*]quinolizin-4-yl)methanone (6b)

	Parameter	Value
1	Solvent	CDCl3
2	Temperature	298.2
3	Pulse Sequence	zg30
4	Number of Scans	16
5	Receiver Gain	128.0
6	Relaxation Delay	2.0000
7	Pulse Width	10.0000
8	Acquisition Time	5.2954
9	Spectrometer Frequency	300.13
10	Spectral Width	6188.1
11	Lowest Frequency	-1246.4
12	Nucleus	1H
13	Acquired Size	32768
14	Spectral Size	65536

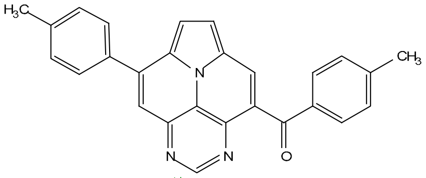

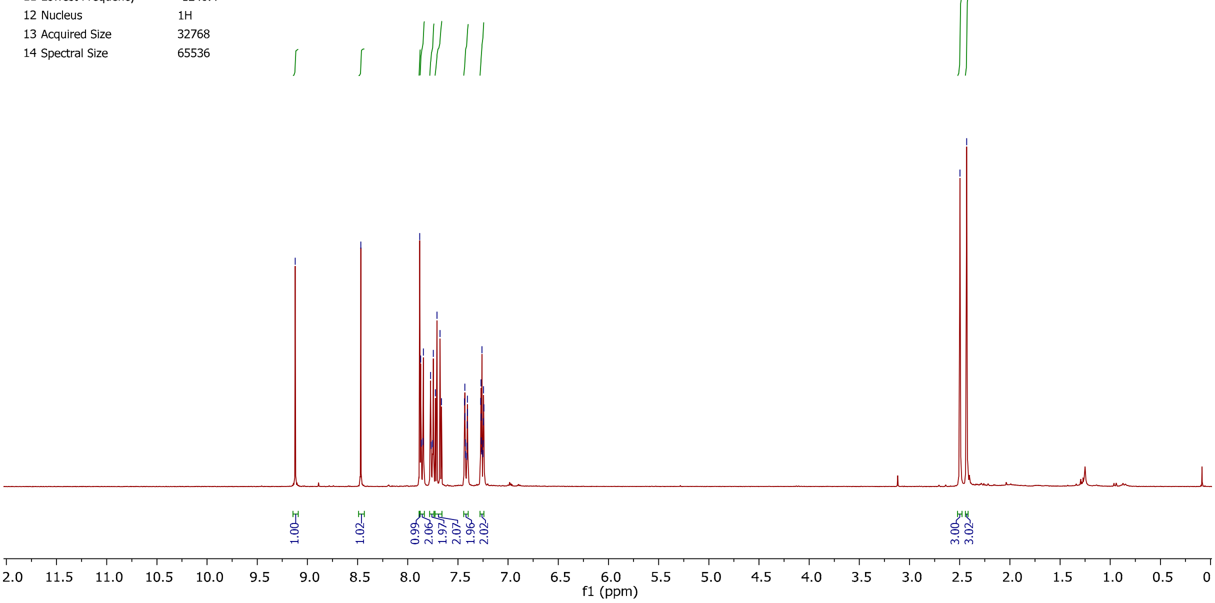

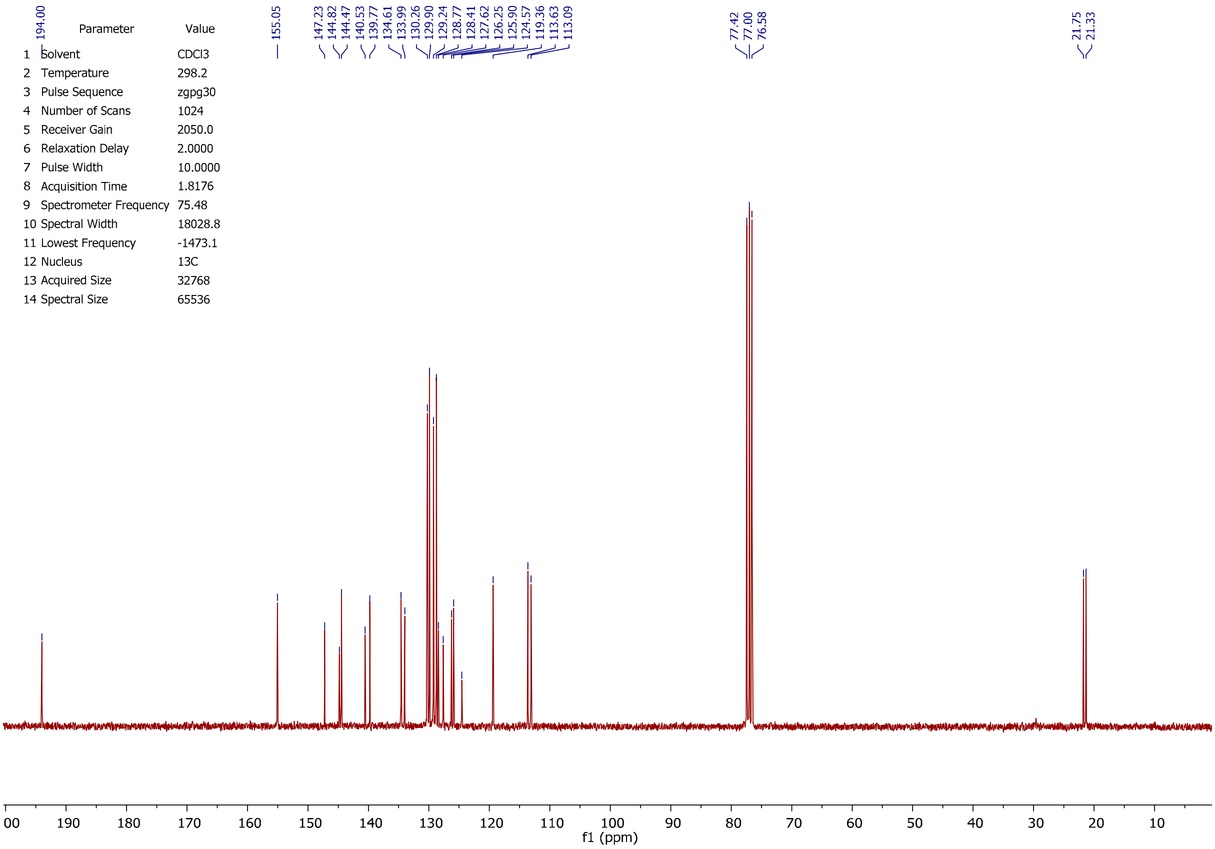

	Parameter	Value
1	Solvent	CDCl3
2	Temperature	298.2
3	Pulse Sequence	zgpg30
4	Number of Scans	1024
5	Receiver Gain	2050.0
6	Relaxation Delay	2.0000
7	Pulse Width	10.0000
8	Acquisition Time	1.8176
9	Spectrometer Frequency	75.48
10	Spectral Width	18028.8
11	Lowest Frequency	-1473.1
12	Nucleus	13C
13	Acquired Size	32768
14	Spectral Size	65536

(4-fluorophenyl)(8-(4-fluorophenyl)pyrimido[4,5,6-*ij*]pyrrolo[2,1,5-*de*]quinolizin-4-yl)methanone (6c)

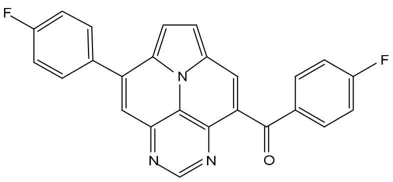

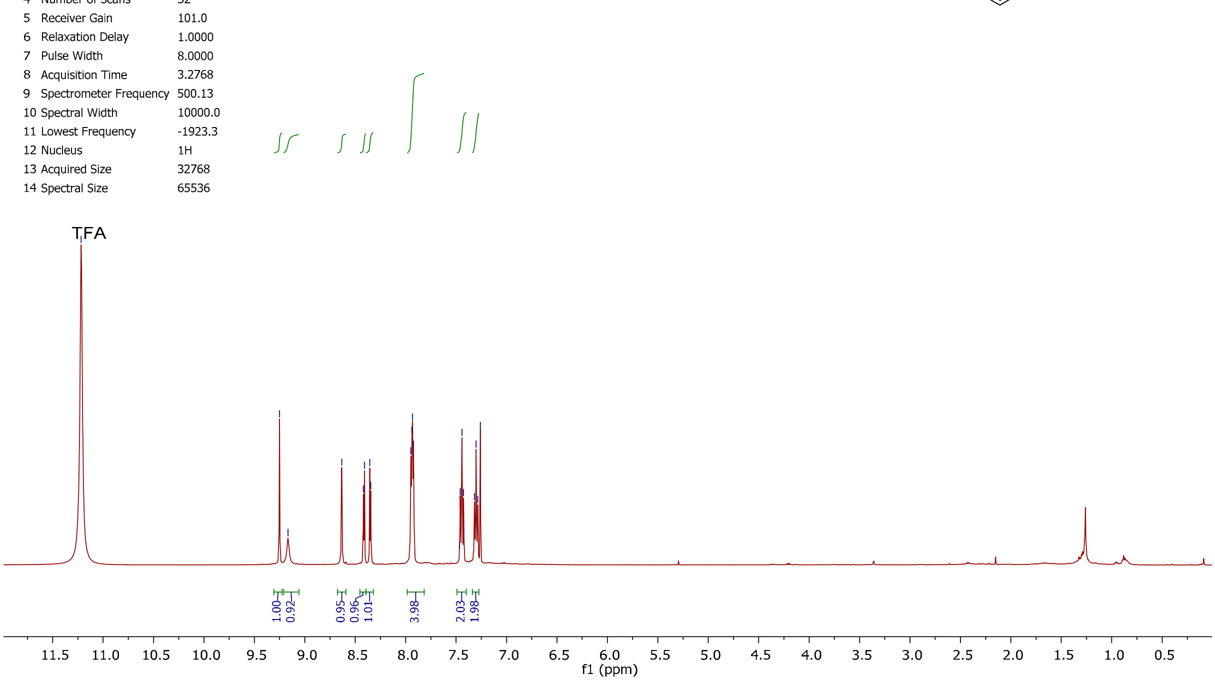

	Parameter	Value
1	Solvent	CDCl3/TFA
2	Temperature	298.1
3	Pulse Sequence	zg30
4	Number of Scans	32
5	Receiver Gain	101.0
6	Relaxation Delay	1.0000
7	Pulse Width	8.0000
8	Acquisition Time	3.2768
9	Spectrometer Frequency	500.13
10	Spectral Width	10000.0
11	Lowest Frequency	-1923.3
12	Nucleus	1H
13	Acquired Size	32768
14	Spectral Size	65536

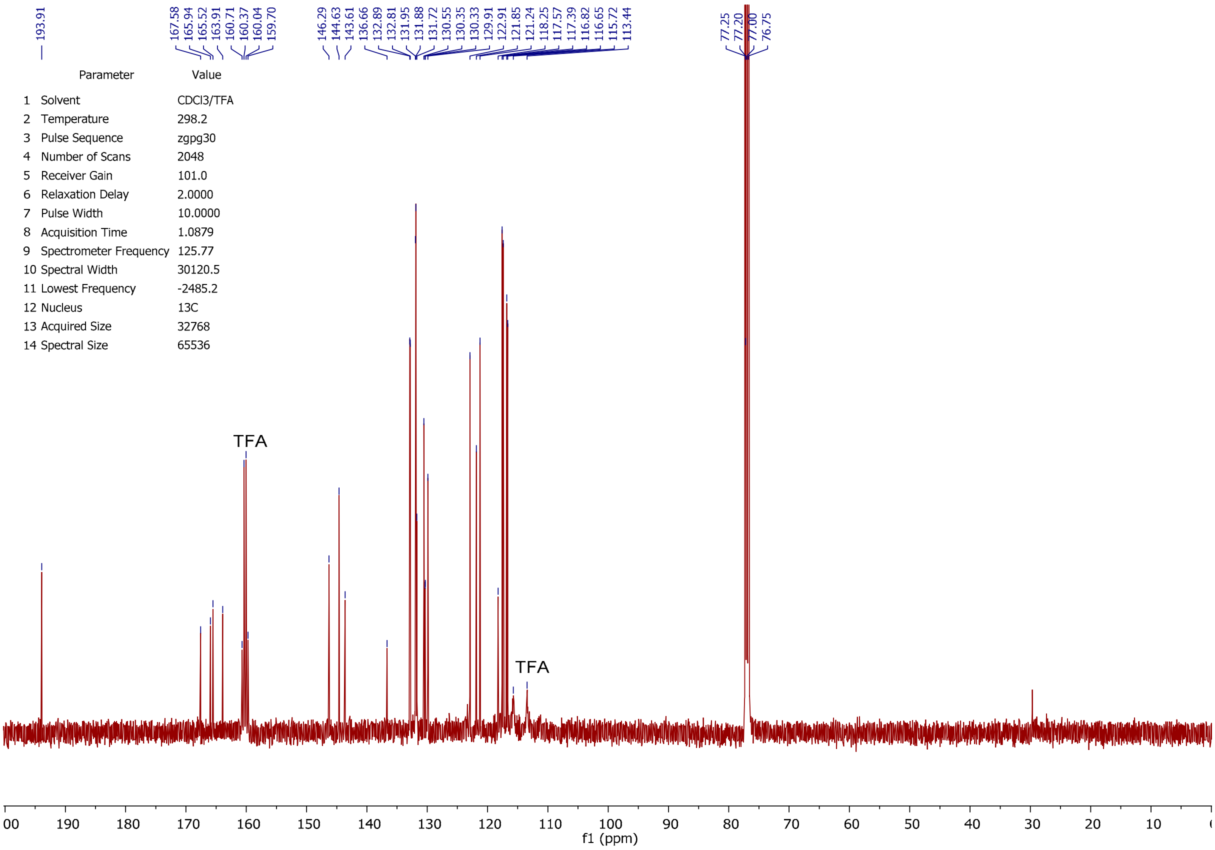

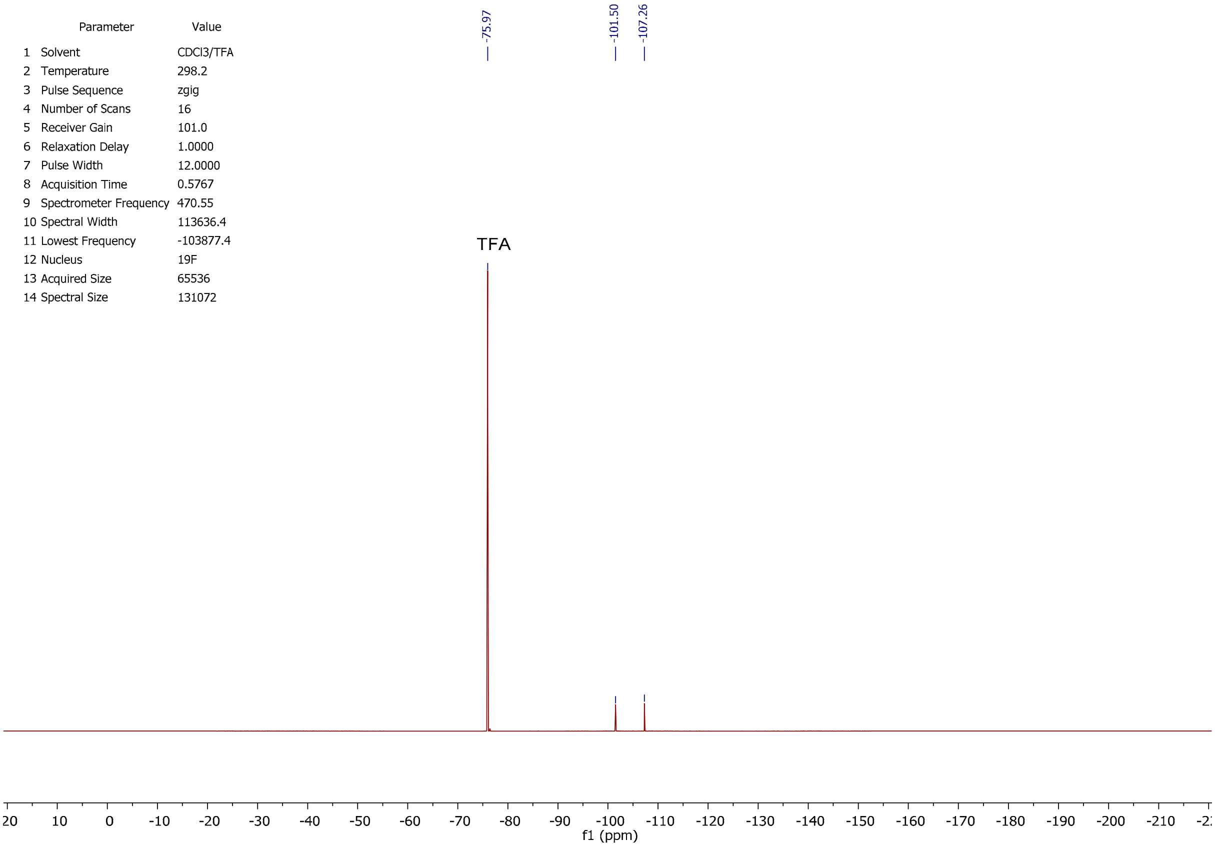

(4-(dimethylamino)phenyl)(8-(4-(dimethylamino)phenyl)pyrimido[4,5,6-*ij*]pyrrolo[2,1,5-*de*]quinolizin-4-yl)methanone (6d)

	Parameter	Value
1	Solvent	CDCl3
2	Temperature	298.1
3	Pulse Sequence	zg30
4	Number of Scans	32
5	Receiver Gain	101.0
6	Relaxation Delay	1.0000
7	Pulse Width	8.0000
8	Acquisition Time	3.2768
9	Spectrometer Frequency	500.13
10	Spectral Width	10000.0
11	Lowest Frequency	-1924.1
12	Nucleus	1H
13	Acquired Size	32768
14	Spectral Size	65536

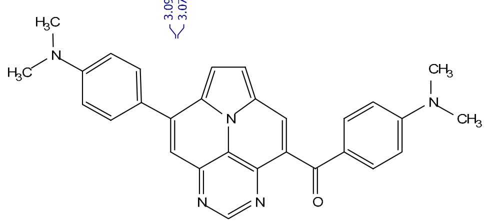

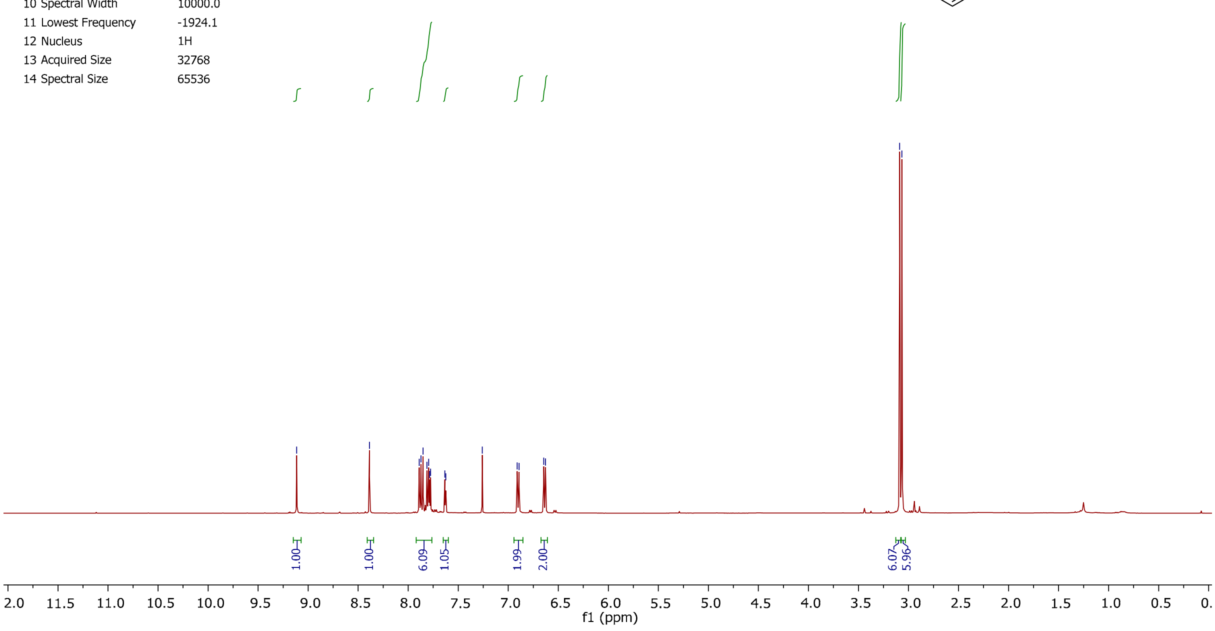

	Parameter	Value
1	Solvent	CDCl3
2	Temperature	298.1
3	Pulse Sequence	zgpg30
4	Number of Scans	2048
5	Receiver Gain	101.0
6	Relaxation Delay	2.0000
7	Pulse Width	10.0000
8	Acquisition Time	1.0879
9	Spectrometer Frequency	125.77
10	Spectral Width	30120.5
11	Lowest Frequency	-2492.0
12	Nucleus	13C
13	Acquired Size	32768
14	Spectral Size	65536

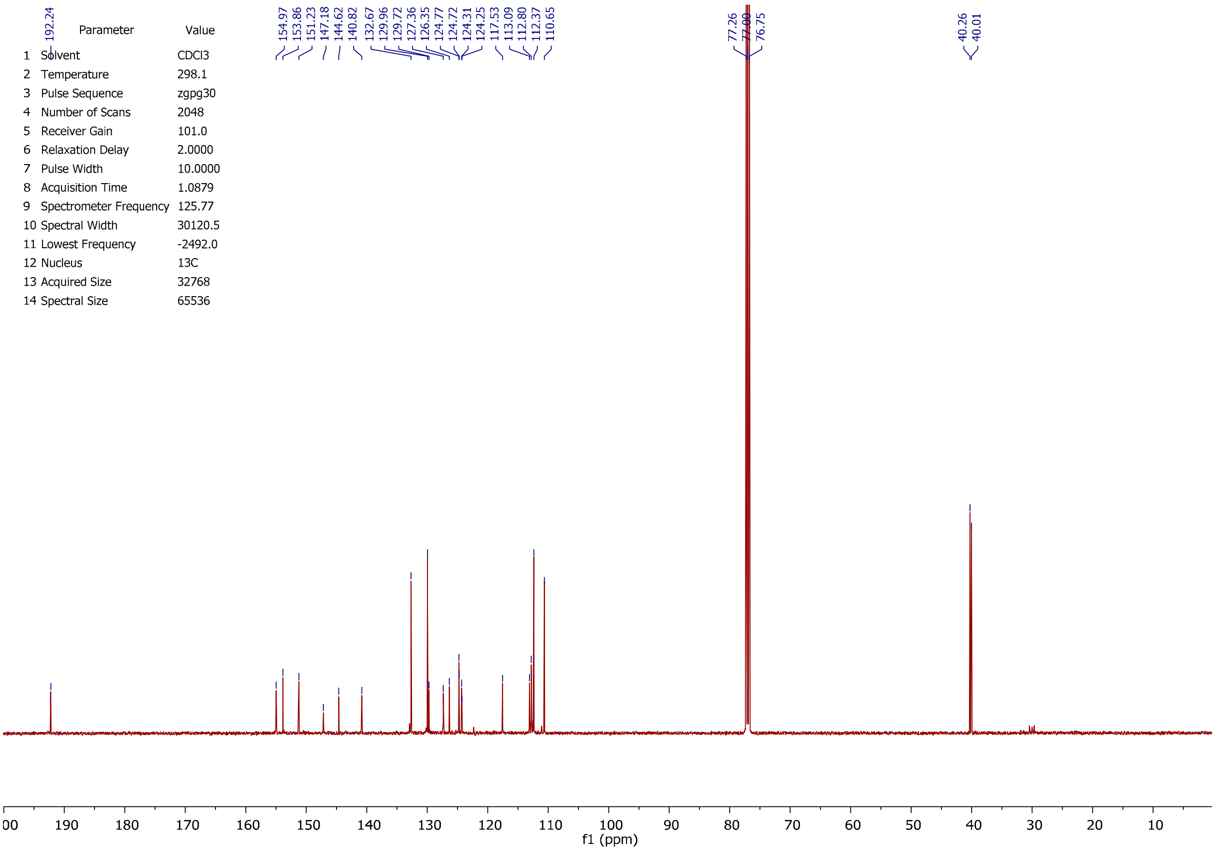

(4-methoxyphenyl)(8-(4-methoxyphenyl)pyrimido[4,5,6-*ij*]pyrrolo[2,1,5-*de*]quinolizin-4-yl)methanone (6e)

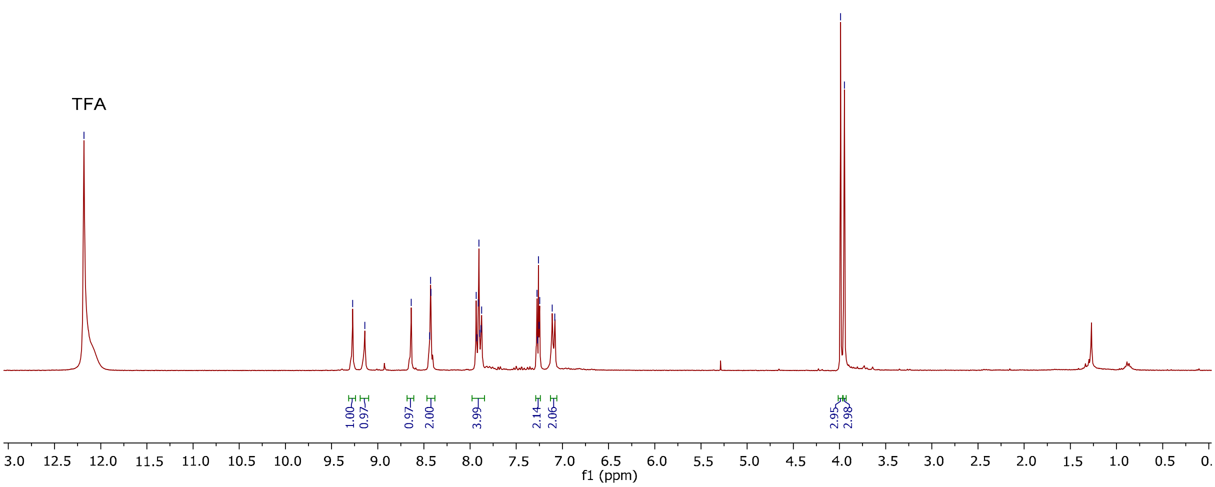

	Parameter	Value
1	Solvent	CDCl3/TFA
2	Temperature	298.2
3	Pulse Sequence	zg30
4	Number of Scans	16
5	Receiver Gain	90.5
6	Relaxation Delay	2.0000
7	Pulse Width	10.0000
8	Acquisition Time	5.2954
9	Spectrometer Frequency	300.13
10	Spectral Width	6188.1
11	Lowest Frequency	-1246.5
12	Nucleus	1H
13	Acquired Size	32768
14	Spectral Size	65536

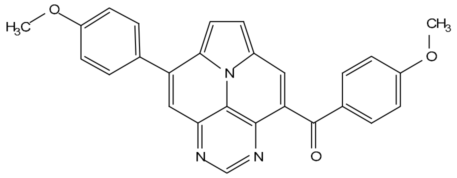

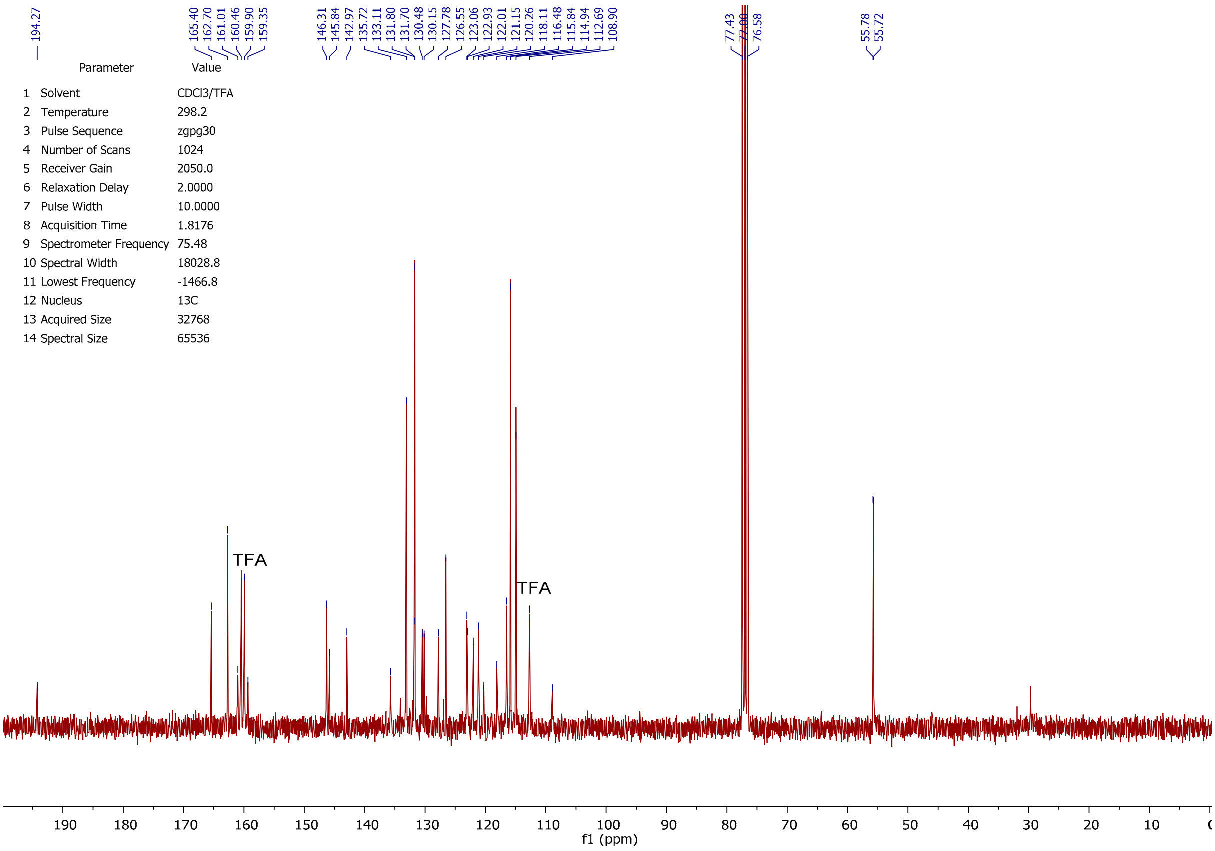

	Parameter	Value
1	Solvent	CDCl3/TFA
2	Temperature	298.2
3	Pulse Sequence	zgpg30
4	Number of Scans	1024
5	Receiver Gain	2050.0
6	Relaxation Delay	2.0000
7	Pulse Width	10.0000
8	Acquisition Time	1.8176
9	Spectrometer Frequency	75.48
10	Spectral Width	18028.8
11	Lowest Frequency	-1466.8
12	Nucleus	13C
13	Acquired Size	32768
14	Spectral Size	65536

(4-(trifluoromethyl)phenyl)(8-(4-(trifluoromethyl)phenyl)pyrimido[4,5,6-*ij*]pyrrolo[2,1,5-*de*]quinolizin-4-yl)methanone (6f)

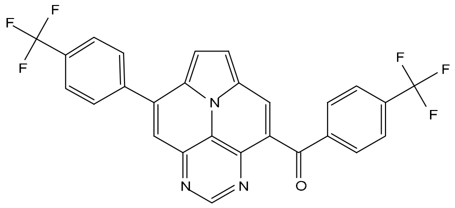

	Parameter	Value
1	Solvent	CDCl3/TFA
2	Temperature	298.1
3	Pulse Sequence	zg30
4	Number of Scans	16
5	Receiver Gain	101.0
6	Relaxation Delay	1.0000
7	Pulse Width	8.0000
8	Acquisition Time	3.2768
9	Spectrometer Frequency	500.13
10	Spectral Width	10000.0
11	Lowest Frequency	-1922.8
12	Nucleus	1H
13	Acquired Size	32768
14	Spectral Size	65536

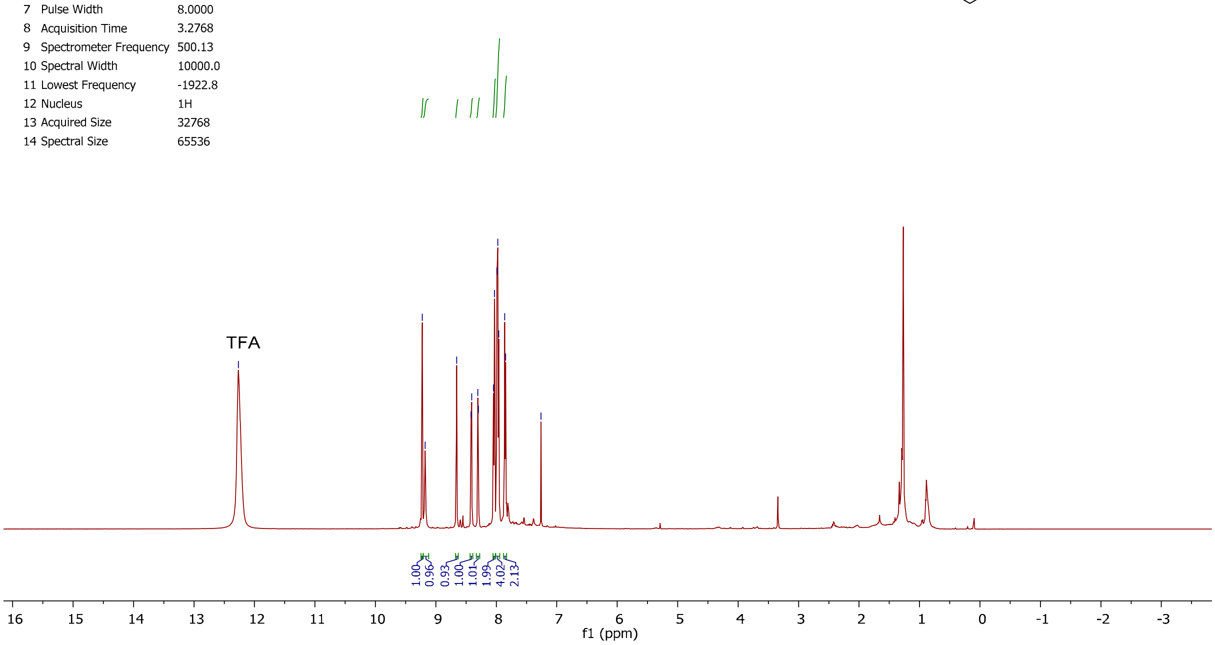

	Parameter	Value
1	Solvent	CDCl3/TFA
2	Temperature	298.2
3	Pulse Sequence	zgpg30
4	Number of Scans	2048
5	Receiver Gain	101.0
6	Relaxation Delay	2.0000
7	Pulse Width	10.0000
8	Acquisition Time	1.0879
9	Spectrometer Frequency	125.77
10	Spectral Width	30120.5
11	Lowest Frequency	-2486.0
12	Nucleus	13C
13	Acquired Size	32768
14	Spectral Size	65536

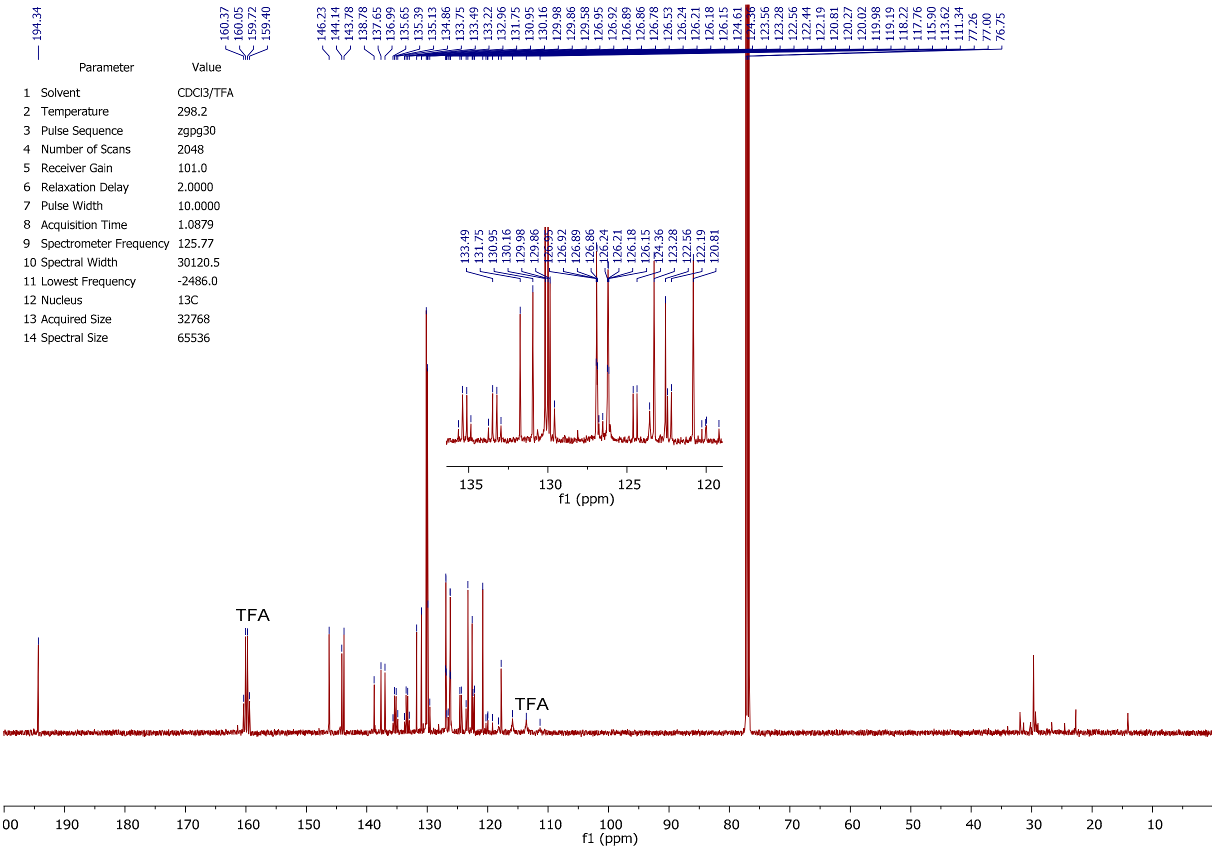

	Parameter	Value
1	Solvent	CDCl3/TFA
2	Temperature	298.2
3	Pulse Sequence	zgig
4	Number of Scans	16
5	Receiver Gain	101.0
6	Relaxation Delay	1.0000
7	Pulse Width	12.0000
8	Acquisition Time	0.5767
9	Spectrometer Frequency	470.55
10	Spectral Width	113636.4
11	Lowest Frequency	-103877.4
12	Nucleus	19F
13	Acquired Size	65536
14	Spectral Size	131072

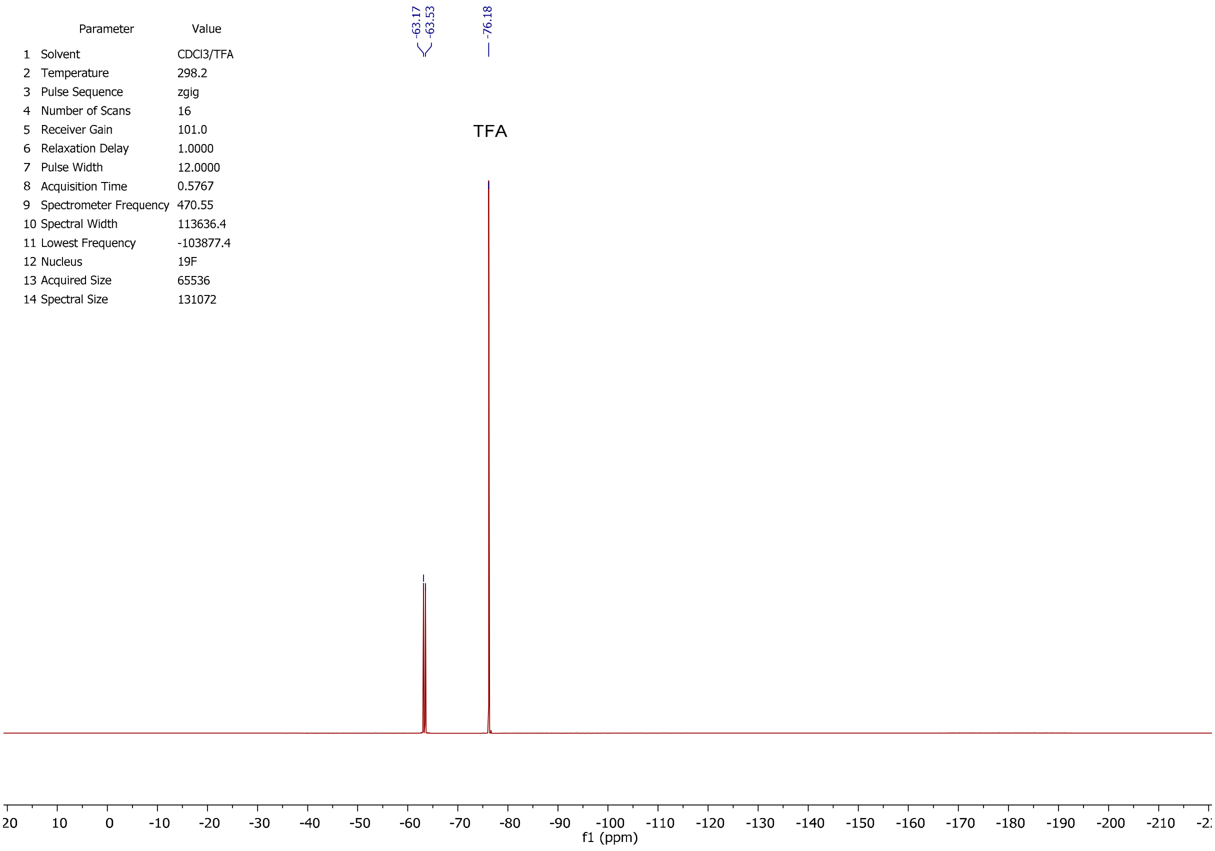

REFERENCES

1. Xiang, J.; Zheng, L.; Chen, F.; Dang, Q.; Bai, X. A cascade reaction consisting of Pictet-Spengler-type cyclization and Smiles rearrangement: application to the synthesis of novel pyrrole-fused dihydropteridines. *Org. Lett.*, **2007**, *9,* 765–767.
2. Brouwer, A.M. Standards for photoluminescence quantum yield measurements in solution (IUPAC Technical Report). *Pure Appl. Chem.*, **2011**, *83,* 2213–2228.
3. Frisch, M.J.; Trucks, G.W.; Schlegel, H.B.; Scuseria, G.E.; Robb, M.A.; Cheeseman, J.R.; Scalmani, G.; Barone, V.; Petersson, G.A.; Nakatsuji, H.; Li, X.; Caricato, M.; Marenich, A.V.; Bloino, J.; Janesko, B.G.; Gomperts, R.; Mennucci, B.; Hratchian, H.P.; Ortiz, J.V.; Izmaylov, A.F.; Sonnenberg, J.L.; Williams; Ding, F.; Lipparini, F.; Egidi, F.; Goings, J.; Peng, B.; Petrone, A.; Henderson, T.; Ranasinghe, D.; Zakrzewski, V.G.; Gao, J.; Rega, N.; Zheng, G.; Liang, W.; Hada, M.; Ehara, M.; Toyota, K.; Fukuda, R.; Hasegawa, J.; Ishida, M.; Nakajima, T.; Honda, Y.; Kitao, O.; Nakai, H.; Vreven, T.; Throssell, K.; Montgomery Jr., J.A.; Peralta, J.E.; Ogliaro, F.; Bearpark, M.J.; Heyd, J.J.; Brothers, E.N.; Kudin, K.N.; Staroverov, V.N.; Keith, T.A.; Kobayashi, R.; Normand, J.; Raghavachari, K.; Rendell, A.P.; Burant, J.C.; Iyengar, S.S.; Tomasi, J.; Cossi, M.; Millam, J.M.; Klene, M.; Adamo, C.; Cammi, R.; Ochterski, J.W.; Martin, R.L.; Morokuma, K.; Farkas, O.; Foresman, J.B.; Fox, D.J. *Gaussian 16 Rev. C.01:* Wallingford, CT, **2016**.
4. Paenurk, E.; Gershoni-Poranne, R. Simple and efficient visualization of aromaticity: bond currents calculated from NICS values. *Phys. Chem. Chem. Phys.*, **2022**, *24,* 8631–8644.
